# Erqember Mitigates Neurotoxic Effects of Aluminum Chloride in Mice: Phytochemical Insights With Neurobehavioral and In Silico Approaches

**DOI:** 10.1155/jt/3997995

**Published:** 2025-04-02

**Authors:** Habiba Khan, Sana Javaid, Waseem Ashraf, Farhan Siddique, Mehvish Bibi, Tanveer Ahmad, Muhammad Shoaib Ali Gill, Asad Abrar, Faleh Alqahtani, Imran Imran

**Affiliations:** ^1^Department of Pharmacology, Faculty of Pharmacy, Bahauddin Zakariya University, Multan 60800, Pakistan; ^2^Department of Pharmacy, The Women University, Multan, Pakistan; ^3^Department of Pharmaceutical Chemistry, Faculty of Pharmacy, Bahauddin Zakariya University, Multan 60800, Pakistan; ^4^Institut pour l'Avancée des Biosciences, Centre de Recherche UGA, INSERM U1209, CNRS 5309, Université Grenoble Alpes, Grenoble, France; ^5^The Institute of Pharmaceutical Sciences, University of Veterinary & Animal Sciences, Lahore 75270, Pakistan; ^6^Drug Testing Laboratory, Bahawalpur, Punjab, Pakistan; ^7^Department of Pharmacology and Toxicology, College of Pharmacy, King Saud University, Riyadh 11451, Saudi Arabia

**Keywords:** aluminum chloride, amnesia, behavior studies, Erqember, herbal preparations

## Abstract

The increasing popularity of herbal preparations has prompted people around the world to incorporate herbal products into their balanced diet, aiming to improve brain health and protect against neurological disorders. Erqember(Erq-Em) possesses a blend of various neuroprotective phytocompounds. The present study aimed to phytochemically analyze this polyherbal product and scientifically validate its neurological benefits. After chemical characterization through UHPLC-MS, in vivo studies involved the supplementation of mice with 10 and 20 mL/kg doses of Erq-Em in an AlCl_3_-induced amnesic mice model followed by behavioral assessment for anxiety and cognition in a battery of behavioral tests. Subsequently, whole brains were dissected for biochemical and histopathological analysis. Further, the study also included in silico studies to understand the interaction of detected phytocompounds with acetylcholinesterase protein. The outcomes revealed that mice treated with Eqr-Em were protected from anxiety-like behavior as they dose-dependently prefer innately frightening central, lightened, and elevated zones in OFT, L/D, and EPM tests. Moreover, the Erq-Em supplementation caused improved spontaneous learning in Y-maze and NOR tests, while their memory in passive avoidance and water maze tests was evident from longer step-through and shorter escape latencies, respectively. The biochemical analysis of brain homogenates showed a reduction in AchE and MDA while elevation in SOD and GPx levels in mice receiving Erq-Em. Moreover, the healthy and intact neuronal counts were markedly high in CA1 and DG regions of Nissl's-stained hippocampi of Erq-Em-treated mice. The compounds detected by UPLC-MS showed favorable BBB permeability and interacted well with acetylcholinesterase protein through in silico studies. Overall, the neurological benefits of Erqember might result from enhanced cholinergic neurotransmission and antioxidative activity of its phytocompounds, which together function as multimodal strategies against AlCl_3_-induced neurotoxicity.

## 1. Introduction

Neurological ailments affect people worldwide as one in every three people may experience a neurological condition according to the World Health Organization. People of any age and gender might be affected by neurological disorders, but Alzheimer's disease is a developing neurological condition characterized by a gradual buildup of tau tangles and beta-amyloid plaques leading to neurodegeneration in the medial temporal lobe and cortical structures. The role of cholinergic neurotransmission is well known in the learning and memory process and literature reports that 75% of cholinergic neurons are damaged in the late stages of AD [1]. The treatment of AD mainly relies on retrieving the cholinergic function by using anticholinesterases [2]. The risk of adverse effects associated with the use of anticholinesterases makes it important to look for a substitute to ameliorate the development and progression of AD. [3]. Neurological disorders cause a huge burden of life-long disabilities and deaths, particularly in low–middle-income countries, and this menace is expected to increase in the next decades [4]. People around the globe are progressively displaying their interest in commercially available herbal products to treat neurological illnesses due to the cost-effectiveness and better safety profiles of such natural products [5].

Aluminum chloride (AlCl_3_) is inevitably abundant in the environment and can easily cross the blood–brain barrier (BBB), resulting in elevation of the brain oxidative stress and impaired cholinergic neurotransmission [6]. It has been disclosed by numerous studies that exposure to heavy metals is associated with the development of neurological ailments including AD. Aluminum is one of the heavy metals and its exposure can change the BBB leading to its accumulation and alteration in the antioxidant enzyme activity and neurochemical balance in the brain [7]. It has been validated through experimentation that long-term exposure to aluminum triggers the changes in brain mimicking the human AD, thus AlCl_3_-induced AD model is a widely used laboratory method to study the neuroprotective potential of natural and/or synthetic constituents against AD [8, 9].

As the availability and affordability of medicines is challenging for people residing in various countries, they are increasingly trusting on herbal medications due to their safety, easy approachability, and cost-effectiveness [10]. One of the commercially available polyherbal preparations is “Erqember” prepared by Hamdard Laboratories Pakistan. This polyherbal preparation has been claimed to be beneficial for vital organs including the brain as its use encompasses the enhancement of mental health. The commercially available packing of 175 mL contains *Punica granatum* (0.375 mL), *Elettaria cardamomum* (0.0312 gm), *Malus sylvestris* (0.1875 mL), *Myrtus caryophyllus* (0.0312 gm), *Aquilaria agallocha* (0.0312 mg), *Ocimum sanctum* (0.0312 gm), *Amomum subulatum* (0.0312 gm), *Onosma bracteatum* (0.0312 gm), *Nepeta ruderalis* (0.0312 gm), *Parmelia perlata* (0.0312 gm), *Pimpinella anisum* (0.0312 gm), *Asparagus racemosus* (0.0312 gm), *Pistacia vera* (0.0312 gm), *Bambusa arundinacea* (0.0312 gm), *Pyrethrum indicum* (0.0312 gm), *Bombyx mori* (0.0312 gm), *Rosa damascene* (0.0312 gm), *Cinnamomum malabathrum* (0.0312 gm), *Salvia haematodes* (0.0312 gm), *Cinnamomum tamala* (0.0312 gm), *Santalum album* (0.0312 gm), *Cinnamomum zeylanicum* (0.0312 gm), *Valeriana officinalis* (0.0312 gm), *Citrus medica* (0.0312 gm), *Zingiber zerumbet* (0.0312 gm), *Coriandrum sativum* (0.0312 gm), *Ambra grasea* compound (0.02 gm), *Cymbopogon jwarancusa* (0.0312 gm), *Crocus sativus* (0.000625 gm), and *Cyperus rotundus* (0.0312 gm). Among these herbal components, *Onosma bracteatum*, *Doronicum hookeri*, and *Ambra grasea* compounds have been claimed to possess neuroprotective effects.

The current study aimed to validate the professed neurological benefits of broadly used polyherbal preparation “Erqember”. The preparation was initially analyzed for its phytochemical composition while the in vivo experimentation was carried out using aluminum chloride–induced mice model of amnesia. The mice were supplemented with Erqember for few weeks followed by their testing for anxiety and cognition in a series of behavioral experiments. Later, whole brains were isolated for biochemical and histopathological studies, and in silico studies were performed to understand the nature of the interaction of potential compounds with the target protein.

## 2. Materials and Methods

### 2.1. Chemicals and Drugs

The commercially available Erqember (Erq-Em) was procured from Hamdard Laboratories, Pakistan. Aluminum chloride was purchased from Merck, and its 200 mg/kg dilution was made using tap water [11]. Donepezil was purchased from Across Organics and dissolved in distilled water to be intraperitoneally (i.p.) injected at a dose of 5 mg/kg [12].

### 2.2. Chemical Characterization by UHPLC-MS

The chemical composition of Erq-Em was examined by ultrahigh-performance liquid chromatography-mass spectrometry (UHPLC-MS) analysis. The Agilent 1290 Infinity LC system coupled with Agilent 6520 Accurate-Mass Q-TOF mass spectrometer with dual ESI source (Agilent technology, USA) was employed for the analysis by using column Agilent Zorbax Eclipse XDB-C18, narrow-bore 2.1 150 mm, 3.5 μm. The temperature of the column was maintained at 25°C, while auto-sampler was regulated at 4°C. The 1.0 μL of the sample was injected followed by maintaining the flow at 0.5 mL/minute. The sample run time was 25 min, and the post-run time was 5 min. The mobile phases used in the analysis were formic acid (0.1%) (A) and acetonitrile (B). Full-scan MS analysis was done using negative electrospray ionization mode, over a range of m/z 100–1000. Nitrogen was supplied at flow rates of 25 and 600 L/h as nebulizing and drying gas, respectively and the drying gas temperature was kept at 350 C. The capillary voltage was set to 3500 V, while the fragmentation voltage was optimized to 125 V. Obtained data was examined through Agilent Mass Hunter software (B.05.00) for qualitative analysis (Method: Metabolomics-2019.m), and the detected compounds were identified through Search Database (METLIN_AM_PCDL-N- 170502.cdb).

### 2.3. Animals

6–8 –week-old BALB/C mice weighing 25–30 g were used in this study. The mice were bred and housed in the animal house situated at the Faculty of Pharmacy, Bahauddin Zakariya University, Multan. The animal housing conditions were maintained at 23°C ± 2°C and 30%–70% humidity, while a 12-h light/dark cycle was provided to all animals. The mice were housed in clear Plexiglas cages provided with sawdust bedding which were renewed weekly by skilled caretakers. All animal studies were conducted after obtaining the permission from Departmental Ethical Committee (13-PHL-S21), and all studies were in accordance to the guidelines provided by Institute of Laboratory Animal Resources (ILAR) and the National Research Council (NRC, 1996).

#### 2.3.1. Animal Grouping and Treatment

A total of 40 animals were randomly divided into five groups (*n* = 8) as follows:

Healthy group comprised healthy mice that received normal feed and tap water.

AlCl_3_-treated control group comprised amnesic mice that received normal feed and water bottles comprising AlCl_3_ (200 mg/kg).

Standard group comprised mice that received normal feed and water bottles comprising AlCl_3_ (200 mg/kg) with once daily dose of donepezil (5 mg/kg; i.p.).

Erq-Em 10 group comprised mice that received Erq-Em (10 mL/kg) added to normal feed and water bottles comprising AlCl_3_ (200 mg/kg).

Erq-Em 20 group comprised mice that received Erq-Em (20 mL/kg) added to normal feed and water bottles comprising AlCl_3_ (200 mg/kg).

**Figure 1:**
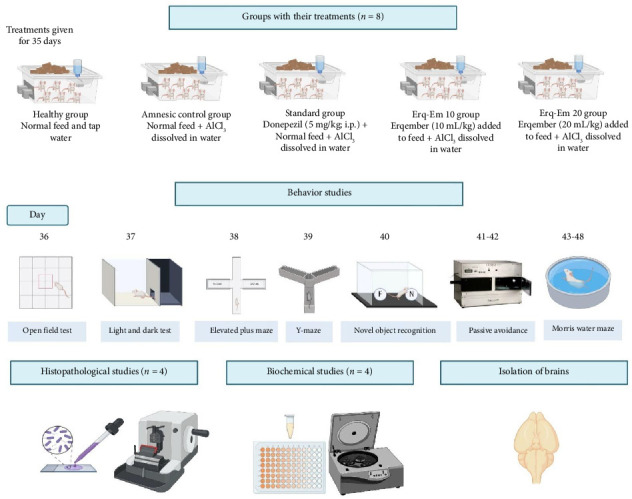
The outline depicts animal grouping and respective treatments for in vivo experimentation to evaluate the neuroprotective potential of a commercially available herbal formulation named “Erqember” (Erq-Em). The study included five groups of BALB/c mice receiving group-wise treatments for 35 days followed by neurobehavioral evaluation through a battery of behavioral experiments. Subsequently, the brains were dissected for biochemical and histopathological evaluation. The illustration of the experimental scheme has been created through Biorender.com (CY277HFPBJ; dated August, 21, 2024).

Except for healthy mice, animals of all other groups were provided with aluminum chloride dissolved in tap water by placing the water bottles in cages. Erq-Em at 10 and 20 mL/kg was mixed with rodent feed and provided to the mice of respective groups [13]. The animals received particular treatments for 35 days followed by behavioral assessment for anxiety and cognition. Before each test, the mice were shifted to the behavior room and permitted to acclimate to the environment for at least 1 h. The series of behavior studies were arranged from least to most aversive test (depicted in Figure 1) and all testing was conducted from 8:00 a.m. to 6:00 p.m. by an experimenter who was kept unaware of group-wise treatments to avoid the possibility of bias in outcomes. The animal's activity during behavior tests was video-recorded through the camera (Logitech) and assessed by ANYmaze software (Full License Version).

### 2.4. Tests for Anxiety

#### 2.4.1. Open Field Test (OFT)

This test was performed to evaluate the effects of Erq-Em on a mouse's anxiety-like activity. The test was performed in a square maze comprising an open area of 45 × 45 cm surrounded by walls to avoid animal escape during the test. On the 36th day of study, the mice were placed in this maze and allowed to explore it for 5 min while their activity was video-recorded. The recorded videos were later evaluated for mice's preference for peripheral and central zones of the maze [14].

#### 2.4.2. Light and Dark (L/D) Box Test

On the 37th day, the mice were introduced to an apparatus comprising two interconnected chambers; one chamber was lit (21 × 21 × 25 cm), while the other was dark (20 × 40 × 40 cm). The individual mouse was placed in dark zone and permitted to investigate both chambers for 5 min [15]. The animal's activity in the apparatus was monitored and anxiety-like behavior was estimated as literature reports that increased exploration of illuminated areas depicts reduced fear.

#### 2.4.3. Elevated Plus Maze (EPM) Test

On the 38th day, the mice were tested in a plus-shaped maze comprising four arms each 15 × 5.5 cm in dimensions which were elevated at a height of 45 cm. Out of four arms, two were open and exposed while two were closed and surrounded by walls. The mice were introduced in the center of four arms, and their preference for open and closed areas of the maze was noted for 5 min [16]. This activity was evaluated to estimate the animal's anxiety-like behavior as the inclination towards open arms was portraying reduced anxiety.

### 2.5. Tests for Learning and Memory

#### 2.5.1. Y-Maze

On the 39th day, the mice were tested for their spatial learning by allowing them to explore a Y-shaped maze comprising three arms (A, B, and C) each having dimensions of 40 × 8 × 15 cm. The Y-maze test utilizes rodents' innate tendency to investigate new areas and reluctance to visit recently explored zones. Every mouse was placed in the middle of the maze and given 5 min to investigate all arms and their sequences of arm visits were monitored. Their alteration behavior was noted as ABC, BCA, CAB, etc. [17], and % spontaneous alterations (%SAP) were computed by using the following formula:

(1) $$\begin{array}{c} \%\text{SAP}=\left[\frac{\left(\text{no}.\text{ of alterations}\right)}{\left(\text{Total arm entries}-2\right)}\right]. \end{array}$$

#### 2.5.2. Novel Object Recognition (NOR) Test

The NOR test was conducted on the 40th day to assess the cognitive abilities of mice. The test comprised two phases and was conducted in a square arena of open field (45 × 45 cm). The first phase included the placement of two identical objects in the apparatus and allowing the individual mouse to explore both objects for 5 min. Immediately, one object was replaced with a new object with a distinct color and shape and the mouse was allowed to interact with these two objects for 5 min. The mice's interaction with novel and familiar objects was monitored to estimate their remembrance of familiarized objects, and a discrimination index was noted which reflects the mice's ability to recognize the novel object based on the difference in exploration time between the familiar and novel objects [18].

(2) $$\begin{array}{c} \text{Discrimination index}=\frac{\left(\text{time spent with novel object}-\text{time spent with familiar object}\right)}{\left(\text{time spent with novel object}+\text{time spent with familiar object}\right)}. \end{array}$$

#### 2.5.3. Passive Avoidance (Step-Through) Test

On Day 41 of the study, the mice were tested for their memory of aversive stimuli through the Gemini passive avoidance system (San Diego Instruments, USA). The avoidance system comprised two chambers each 9.5 × 8 × 8 inches in dimensions. One chamber was illuminated and comprised a bulb as a source of light, while the other chamber was dark including a grid floor made of stainless-steel rods to deliver the electric foot shock of 0.5 mA. Both chambers were interconnected through an automated door. The test had three phases designated as the training phase, the post–1 h test phase carried out 1 h after training (test trial 1), and the post–24 h test phase conducted 24 h after training phase (test trial 2). During the training phase on Day 41, each mouse was introduced to an illuminated chamber and permitted to acclimate for 30 s during which the door was kept closed. After 30 s, the door was opened and the mouse was allowed to enter the dark zone and explore it for 150 s. Due to innate curiosity, rodents tend to immediately enter the dark zone. On their entrance into the dark chamber, the door was closed and an electric shock was delivered [19].

After 1 h (test trial 1), the mouse was again introduced into the apparatus while the door was kept open and the exploratory activity was monitored for 5 min. The mice with cognitive impairment had a reduced ability to recall that the dark zone was linked with aversive stimuli of electric shock and showed reduced latency to enter the dark chamber. Similarly, mice were tested after 24 h (test trial 2) and latency to enter the dark chamber was noted with the cutoff time of 5 min. The increased latency to enter the innately preferred dark chamber showed the remembrance of shock stimuli and better memory of mice.

#### 2.5.4. Morris Water Maze

The mice were tested for long-term spatial memory by allowing them to locate the disguised platform in the Morris water maze. The test apparatus comprised a round water tub (100 × 60 cm) accompanied by proximal and distal geometrical-shaped cues to offer navigational aid to mice. The round maze was divided into four equal quarters (NE, NW, SW, and SE), and the platform was positioned in SW quarter throughout the experiment [20]. The MWM was completed in 6 days (days 43–49) divided into two training days, three test days and one probe day. The mice were trained to reach the visible platform in four trials/day during two initial training days. In subsequent test days, the platform was disguised by adding a nontoxic white tint in water and every mouse was tested once every day. In test days, the time taken by each mouse to escape from the water and reach platform was noted with a cutoff time of 2 min [21]. On probe day, the platform was removed from maze and every mouse was allowed to swim for 2 min to observe the swimming pattern in terms of SW quarter visits and time spent there as these parameters are indicators of their good remembrance of the platform position.

### 2.6. Biochemical Analysis

On Day 50, the mice (*n* = 4) from all groups were euthanized by cervical dislocation and whole brains were isolated. Each brain was individually weighed and homogenized at 4°C for 10 min at 12,000 rcf. using 10 times volume of 0.1 M phosphate buffer saline followed by collection of supernatants for further analysis [22].

#### 2.6.1. Acetylcholinesterase (AchE) Assay

The AchE activity was noted in prepared brain homogenates by using the method in [23], and results were expressed as μmol/min/mg of protein.

#### 2.6.2. Malondialdehyde (MDA) Assay

The levels of MDA were noted by adopting the previously reported method [24], and outcomes were expressed as nmol/mg of protein.

#### 2.6.3. Superoxide Dismutase (SOD) Assay

The SOD activity was noted by adopting the method described earlier [25] and expressed as milliunits/mg of protein.

#### 2.6.4. Glutathione Peroxidase (GPx) Assay

GPX activity was noted by using the previously reported method [26], and results were expressed as nmol/min/mg of protein.

### 2.7. Histopathological Studies

The mice (*n* = 4) from each group were decapitated by cervical dislocation for histopathological analysis of dissected brains. The isolated brains were instantly fixed with 4% formalin in PBS for 3 days at 4°C followed by preparation of paraffin blocks. Subsequently, sections of 10 μm thickness were sliced between −0.90 and −2.30 mm from bregma [27]. The sliced sections after processing were stained using 0.1% cresyl violet as mentioned previously [28]. The sections were examined under an Olympus microscope, and ImageJ software was used to evaluate the impact of AlCl_3_ and Erqember on histopathological changes in cornu ammonis (CA1) and dentate gyrus (DG) regions of the hippocampus while healthy neurons were enumerated at 10×.

### 2.8. Assessment of BBB Permeability of Compounds and Their Molecular Docking Studies

The goal of this study is to focus on assessing the BBB permeability of the ligands that depicted the favorable binding interactions with the target protein in glide molecular docking analysis. The BBB is a notable factor in the development of CNS drugs, establishing it crucial to quickly estimate the BBB permeability of compounds in the early stage of the drug discovery process by using in silico techniques. The capability of ligands to cross the BBB was analyzed by the BBB prediction server. The support vector machine (SVM) [29] and Ligand Classification by Evidence Distance Surface (LiCABEDS) [30] algorithms with MACCS molecular fingerprint were used to predict the BBB permeability of ligands.

Molecular docking provides valuable insight for the assessment of binding affinity and interactions of ligands towards the specific receptor. This method is significantly used to evaluate how the ligands or drug molecules bind to the targeted receptor site, with their optimized orientation [31].

All the docking calculations and visualization of results were performed by Maestro 12.8 [32]. The ligands and proteins were prepared to perform the docking analysis. The ligands used in this context were downloaded from the PubChem database. The ligands were imported to glid software in 3d-sdf format and prepared by using the Lig-prep module [33]. The crystal structure of the protein (PDB ID 4EY7) was retrieved from PDB site. The protein preparation was carried out through preprocessing, optimization, and minimization by Protein Preparation Wizard module [34]. In the preprocessing stage, hydrogen atoms were added, water molecules were deleted, missing side chains and loops were filled, and bond orders were assigned. After optimization of protein orientation, the OPLS3 force field was selected for minimization [35, 36].

### 2.9. Statistical Analysis

All statistical evaluation was carried out using GraphPad Prism version 8.0 software. The data was checked for normality using Kolmogorov–Smirnov and Shapiro–Wilk tests. Parametric one-way ANOVA followed by Dunnet's multiple comparison test was used to analyze all outcomes except step-through latencies, and escape latencies were analyzed by using two-way ANOVA. All data were expressed as mean ± SD, and *p* < 0.05 was considered statistically significant.

## 3. Results

### 3.1. Chemical Characterization of Erqember by UHPLC-MS

When Erq-Em was analyzed by UHPLC-MS, the detailed profile of chemical compounds owned by polyherbal preparation was revealed showing the presence of various compounds. The details of identified compounds including compound names, structures, retention time, and m/z ratio are provided in Supporting File 1.

### 3.2. Effects of Erqember on Anxiety-Like Activity in OFT, L/D, and EPM Tests

**Figure 2:**
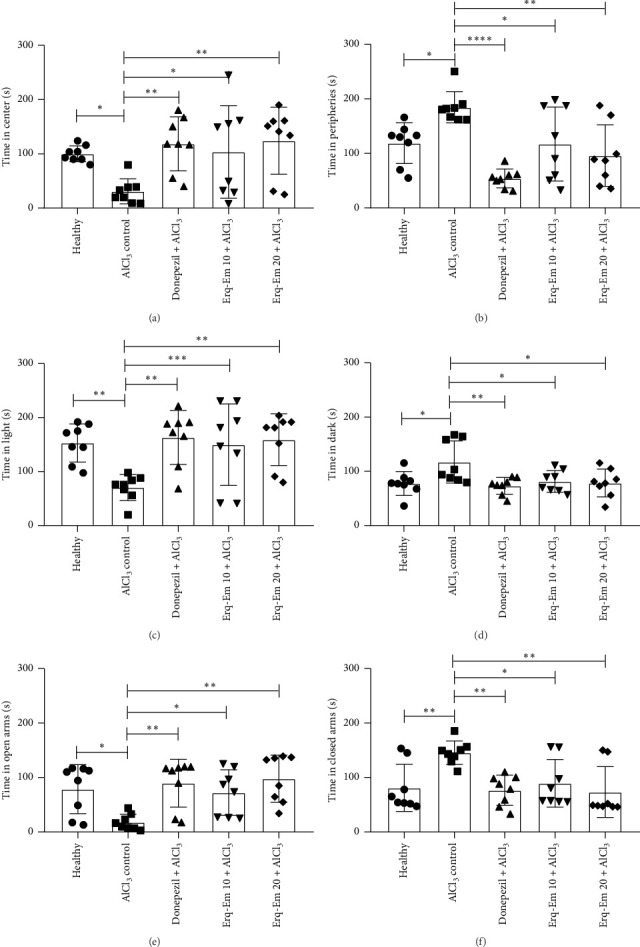
Mice administered with Erqember (Erq-Em) were tested for anxiety-like behavior in OFT, L/D, and EPM tests on Days 36, 37, and 38, respectively. In each test, after allowing to explore the mazes for 5 min, the mice were noted for (a) time spent in the central zone, (b) time spent in the peripheral zone in OFT, (c) time spent in light zone, (d) time spent in dark zone in L/D test, (e) time spent in open arms, and (f) time spent in closed arms of EPM. The outcomes have been presented as mean ± SD (*n* = 8), and ^∗^*p* < 0.05 was considered statistically significant.

When mice were tested in the arena of open field, a notable difference was noted among differently treated mice for time spent in central and peripheral zones with [*F* (4, 35) = 3.91; *p* = 0.009] and [*F* (6, 35) = 8.69; *p* < 0.0001], respectively. The AlCl_3_-treated control mice showed more anxiousness towards the central area and remained confined to the peripheries of the maze for significantly longer (*p* < 0.05), in comparison to healthy mice. The mice treated with Erq-Em showed a dose-dependent improvement in anxiety-like behavior as mice receiving Erq-Em 10 preferred central area more (Figure 2(a)) and avoided peripheral zone (*p* < 0.05) (Figure 2(b)). These outcomes were improved with Erq-Em 20 as preference for central area was markedly enhanced (*p* < 0.01) while partiality to remain confined to peripheries of maze was notably reduced (*p* < 0.01), in comparison to AlCl_3_-treated control mice.

The statistical analysis of outcomes noted in differently treated groups showed a noteworthy difference in time spent in the lightened [*F* (4, 35) = 4.86; *p* = 0.003] and darkened [*F* (4, 35) = 3.92; *p* = 0.009] parts of the apparatus. The detailed analysis indicated that in comparison to healthy mice, AlCl_3_-treated control mice showed a reduced inclination towards the bright part of the maze with *p* < 0.01 and spent more time in the unlit area (*p* < 0.05), indicating their anxiety-like behavior. This anxiety-like behavior was markedly reduced in mice when they were chronically administered with Erq-Em. The mice receiving Erq-Em 10 spent less time in the dark zone (*p* < 0.05) and more time in exploring the lit compartment (*p* < 0.05). These anxiolytic effects were further evident in mice receiving Erq-Em 20 as anxiousness towards dark zone was less (Figure 2(c)) and preference for the illuminated zone was noticeable (*p* < 0.01) (Figure 2(d)), as compared to AlCl_3_-treated control mice.

When mice were tested for their preference for exposed and hidden zones of EPM, a notable inter-group difference was noted for time spent in open arms [*F* (4, 35) = 5.07; *p* = 0.0025] and closed [*F* (4, 35) = 4.97; *p* = 0.0028]. The healthy mice spent more time exploring the open arms while an anxiety-like reluctancy towards open arenas in AlCl_3_-treated mice was evident (*p* < 0.05) as shown in Figure 2(e). Similarly, these AlCl_3_-treated mice preferred to explore hidden zone of maze as they spent more time in closed arms (*p* < 0.01) than healthy mice (Figure 2(f)). These anxiety-like behavior noted after chronic intake of AlCl_3_ was markedly protected in mice supplemented with Erqember in a dose-dependent manner. The mice taking Erq-Em 10 explored open arms for a significantly longer duration (*p* < 0.05) and spent less time in closed arms (*p* < 0.05), as compared to AlCl_3_-treated mice. These outcomes were further intensified in mice consuming Erq-Em 20 as the anxiety-like behavior was notably less (*p* < 0.01) in terms of time spent in open and closed arms, as compared to the AlCl_3_ control group.

### 3.3. Effects of Erqember on Spontaneous Learning and Memory in Y-Maze, NOR, and Passive Avoidance Tests

**Figure 3:**
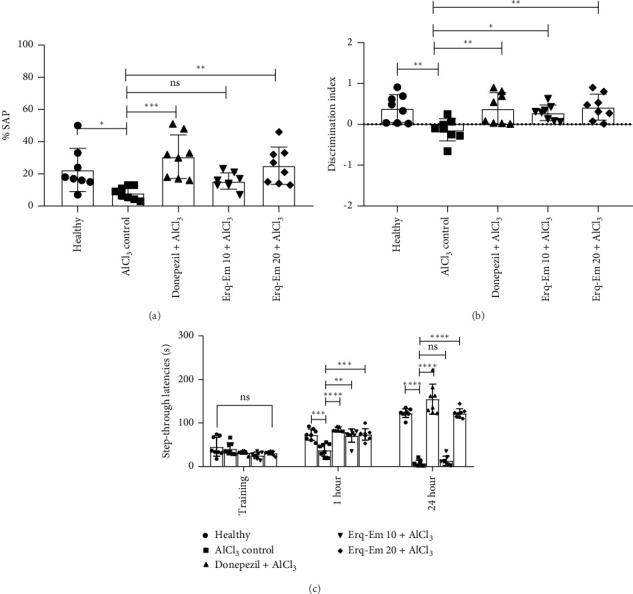
To evaluate the impact of Erqember (Erq-Em) on AlCl_3_-induced amnesia, mice were tested in Y-maze on Day 39, and their exploratory behavior was noted in terms of sequences of arm visits to calculate their (a) % spontaneous alteration. On Day 40, mice were familiarized with two identical objects followed by a test session in which one novel object replaced the previous object to note their (b) discrimination index. On Day 41, the mice were trained to remember the dark compartment of the GEMINI avoidance system by subjecting them to shock stimuli. The recalling ability was tested in two trials carried out after 1 and 24 h of the training phase, and their (c) step-through latencies were monitored. The outcomes have been presented as mean ± SD (*n* = 8), and ^∗^*p* < 0.05 was considered statistically significant while ns presents nonsignificant effects.

The differently treated mice varied significantly for %SAP [*F* (4, 35) = 5.67; *p* = 0.0013]. In detail, the healthy mice remembered the recently visited arm and tended to enter the other adjacently located arms instead of re-entering the previous one. This preference for arm visits resulted in SAP which indicated their good cognitive performance. However, prolonged administration of AlCl_3_ caused a disturbance in the cognitive abilities of mice as their SAP score was notably reduced (*p* < 0.05), as compared to healthy mice. The mice supplemented with Erqember at a dose of 20 mL/kg significantly protected the deteriorative effects of AlCl_3_ as these mice had the ability to recall the immediately visited arm and preferred the other arms to explore resulting in higher SAP behavior (*p* < 0.01), as compared to AlCl_3_-treated amnesic group (Figure 3(a)).

When mice were tested for their ability to discriminate the novel object from the familiarized one, a significant intergroup difference was noted for the discrimination index [*F* (4, 35) = 4.52; *p* = 0.0048]. A thorough analysis of outcomes showed that healthy mice had excellent potential to recall familiar object as they explored novel object more resulting in their good discrimination index score. The consumption of AlCl_3_ caused a loss of commemorating abilities in mice as they were noted to be incapable of differentiating the novel and familiar objects which was evident from their reduced discrimination index (*p* < 0.01). The mice supplemented with Erqember resulted in a dose-dependent protection from AlCl_3_-induced cognitive deficit as mice taking Erq-Em 10 had better discrimination index (*p* < 0.05) than amnesic control mice while these outcomes were further improved in mice taking Erq-Em 20 (*p* < 0.01) as shown in Figure 3(b).

When mice were tested for their memory of aversive stimuli in a passive avoidance test, the two-way ANOVA revealed a noteworthy intergroup difference for step-through latencies [*F* (4, 70) = 104.6; *p* < 0.0001]. During the training session, animals of all groups innately tended to enter the dark compartment of the avoidance system as they were not aware of shock stimuli to be supplied in dark compartment which resulted in a nonsignificant difference in step-through latencies during the training phase [*F* (4, 35) = 3.37; *p* = 0.0196]. However, in post–shock testing carried out after 1 h, the statistical analysis revealed a significant impact of treatment on animal's cognition as healthy mice remembered the shock stimuli well and avoided the dark zone which resulted in longer latencies to step into the shock zone. The chronic intake of AlCl_3_ caused impaired recollection in the amnesic control group as these mice had poor memory of shock and re-entered the dark zone significantly earlier (*p* < 0.001) than healthy mice. The mice supplemented with Erqember were dose-dependently protected from AlCl_3_-induced amnesia as Erq-Em 10 caused mice to remember the shock stimuli after 1 h as they avoided the dark compartment and had noticeably longer step-through latencies (*p* < 0.01). These beneficial outcomes were further intensified in mice supplemented with Erq-Em 20 as the step-through latencies were longer (*p* < 0.001), in comparison to AlCl_3_-treated amnesic mice. Moreover, the mice were again tested after 24 h of training phase to evaluate the impact of treatments on mice's recollection abilities. The ANOVA revealed a significant intergroup difference for step-through latencies [*F* (4, 35) = 118.8; *p* < 0.0001]. The AlCl_3_-treated amnesic mice had compromised remembrance of shock stimuli which was clearly depicted from their reduced avoidance of shock zone and shorter step-through latencies (*p* < 0.0001), in comparison to healthy mice. However, the Erq-Em 10–treated mice varied nonsignificantly for step-through latencies when compared to amnesic mice in a post-shock trial conducted after 24 h. But, the memory-deteriorating effects of AlCl_3_ were noticeably prevented in mice consuming Erq-Em 20 as they had remarkable remembrance of shock zone and stayed in illuminated compartment of avoidance system resulting in their longer step-through latencies (*p* < 0.0001), as compared to amnesic mice as presented in Figure 3(c).

### 3.4. Effects of Erqember on Long-Term Memory in MWM Test

**Figure 4:**
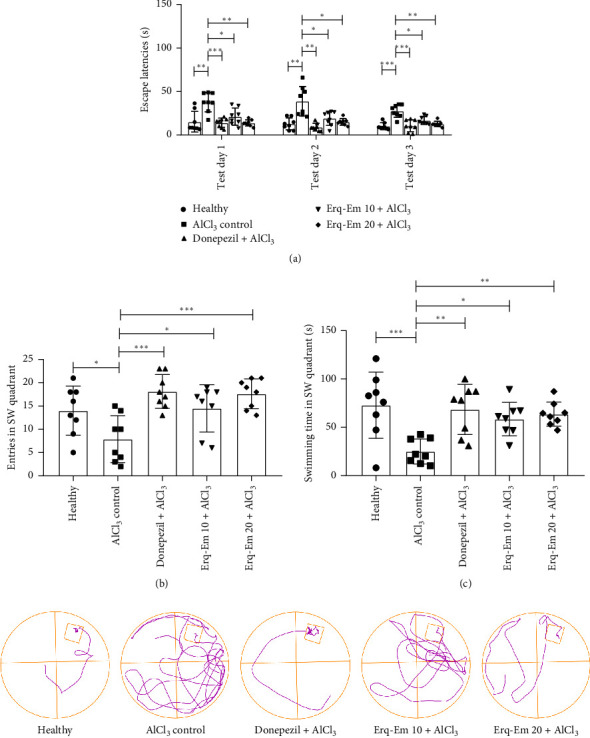
On Days 45–47, the impact of Erqember (Erq-Em) was evaluated on AlCl_3_-induced amnesia by testing the mice in the Morris water maze. Mice were allowed to swim the entire water maze for one trial/day with a cutoff time of 120 s, their ability to locate the disguised platform was monitored, and the time to reach the platform was expressed as (a) escape latencies with representative track plots obtained from ANYMAZE software. On Day 48, the platform was taken out and mice were allowed to swim for 120 s to monitor their cognitive abilities by noticing the (b) entries in SW quadrant and (c) swimming time in SW quadrant. The outcomes have been presented as mean ± SD (*n* = 8), and ^∗^*p* < 0.05 was considered statistically significant.

When mice were tested for their ability to recall the location of the platform and reach it during three test days, a notable intergroup difference in escape latencies was revealed by two-way ANOVA [*F* (4, 70) = 28.43; *p* < 0.0001]. In detail, on test Day 1, healthy mice took significantly less time to locate the disguised platform positioned in SW quadrant of water maze while amnesic mice kept on searching it throughout the maze and rescued themselves noticeably late (*p* < 0.001) as depicted in Figure 4(a). The behavior continued on test Day 2 and test Day 3 as AlCl_3_-induced amnesia was evident from the thigmotaxic behavior as kept on wandering in entire maze in search of rescue platform revealing compromised memory with *p* < 0.01 and *p* < 0.001, in comparison to healthy mice. The mice supplemented with Erqember showed protection from AlCl_3_-induced cognitive deficit in mice dose-dependently. On test Day 1, Erq-Em 10 group located the platform earlier (*p* < 0.05) than the AlCl_3_-treated amnesic group. Likewise, these mice showed appreciable cognitive abilities on test Days 2 and 3 as they escaped themselves in a shorter time and reached the platform notably earlier with *p* < 0.05, in comparison to amnesic mice. The observed outcomes were more beneficial in mice administered with Erq-Em 20 as Erqmeber caused dose-dependent improvement in remembrance of platform position in mice as they found the platform in less time on test days (*p* < 0.01), as compared to amnesic mice.

On probe day, the mice were tested for a trial of 120-s duration in which the platform was taken out from the water maze. The analysis of outcomes through one-way ANOVA showed a noticeable difference for the number of entries [*F* (4, 35) = 6.50; *p* = 0.0005] and swimming time in SW quadrant [*F* (4, 70) = 6.06; *p* = 0.0008]. In detail, the healthy mice remembered the quadrant where the platform was positioned during training and test phases which was depicted from their frequent visits (Figure 4(b)) and swimming time in SW quadrant (Figure 4(c)). Conversely, the AlCl_3_-induced cognitive inabilities in the amnesic control mice were observable as they traveled in all quadrants resulting in their reduced entries (*p* < 0.05) and duration of swimming (*p* < 0.001) in SW quadrant. The supplementation with Erqember protected the mice from AlCl_3_-induced amnesia as Erq-EM 10 mice had better recalling ability which was evident from increased entries (*p* < 0.05) and duration spent (*p* < 0.05) in SW quadrant. Moreover, the neuroprotection was improved in Erq-Em 20 mice as they entered the SW zone more often (*p* < 0.001) and spent more time there (*p* < 0.01) revealing their memory of platform location.

### 3.5. Biochemical Studies

**Figure 5:**
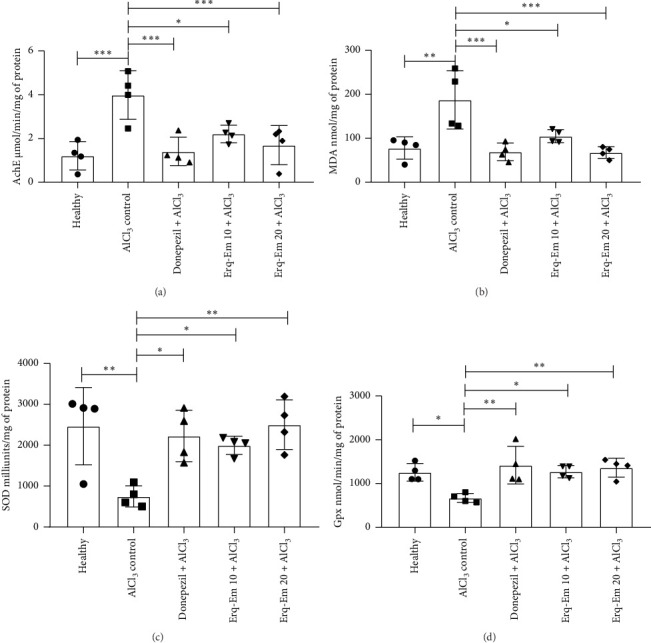
To evaluate the effects of Erqember (Erq-Em) on brain AchE activity and markers of oxidative stress, whole brains were isolated after completion of MWM test and prepared brain homogenates were biochemically analyzed for (a) AchE, (b) MDA, (c) SOD, and (d) GPx. The outcomes have been presented as mean ± SD (*n* = 4), and ^∗^*p* < 0.05 was considered statistically significant.

The outcomes of one-way ANOVA showed that differently treated mice had variation in brain AchE activity [*F* (4, 15) = 8.20; *p* = 0.001]. The mice exposed to AlCl_3_ had markedly increased activity of this enzyme (*p* < 0.001), as compared to healthy brains. However, the consumption of Erqember prevented the AlCl_3_-induced elevation in AchE activity in a dose-dependent manner as Erq-Em 10 had reduced (*p* < 0.05) levels while the outcomes were more significant in mice receiving Erq-Em 20 (*p* < 0.01), in comparison to amnesic group (Figure 5(a)).

Moreover, the brain homogenates analyzed for MDA levels varied prominently depending on treatments [*F* (4, 15) = 8.73; *p* = 0.0008]. The healthy brains had low MDA levels showing reduced lipid peroxidation which was significantly increased by neurotoxic AlCl_3_ (*p* < 0.001) as depicted in Figure 5(b). The mice supplemented with Erq-Em 10 showed reduced levels of MDA (*p* < 0.05), while mice treated with Erq-Em 20 had more protection against AlCl_3_-induced lipid peroxidation revealing dose-dependent neuroprotective benefits of Erqember.

The ANOVA revealed a noticeable intergroup difference for enzyme activity of SOD [*F* (4, 15) = 8.20; *p* = 0.001] and GPx [*F* (4, 15) = 8.20; *p* = 0.001]. In comparison to healthy mice, the AlCl_3_ exposure caused a marked reduction in these endogenously present antioxidative enzymes in amnesic control with *p* < 0.01 and *p* < 0.05, respectively. These deteriorative effects were prevented by Erqember supplementation in mice. The Erq-Em 10 caused the notably higher activity of both enzymes (*p* < 0.05), while in mice provided with Erq-Em 20, these outcomes became more noteworthy (*p* < 0.01) for both enzymes, as compared to amnesic mice as shown in Figures 5(c) and 5(d).

### 3.6. Histopathological Examination

**Figure 6:**
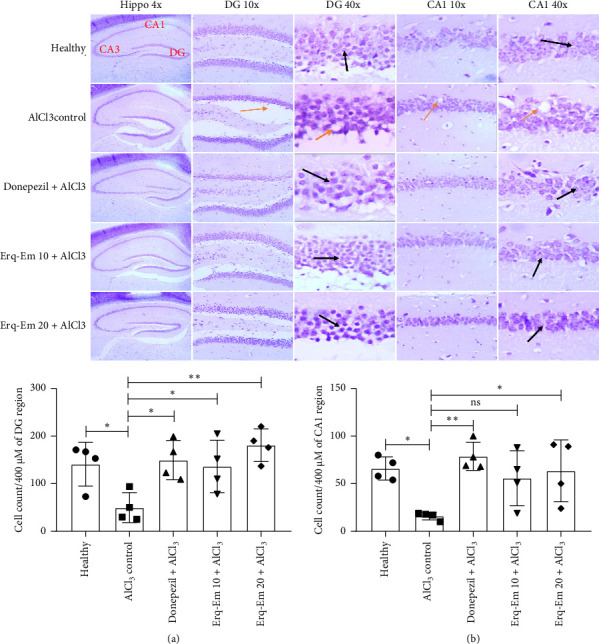
The healthy brains demonstrated intact closely packed cells in DG and CA1 regions of hippocampi. Exposure to AlCl_3_ caused cellular necrosis and derangements in both regions when magnified at 10x and 40x, while Erqember protected the brains from AlCl_3_-induced neurotoxic effects (as black arrows showing healthy neurons and orange arrows pointing the damage). The impact of Erqember on neuronal protection was further evaluated by quantification of healthy neurons in (a) DG region and (b) CA1 region of hippocampus. The outcomes have been presented as mean ± SD (*n* = 4), and ^∗^*p* < 0.05 was considered statistically significant.

When analyzed after Nissl's staining, the hippocampi of healthy brains had well-organized and tightly packed neuronal arrangements comprising defined cellular structures. The prolonged intake of AlCl_3_ resulted in damaged hippocampus as studied sections comprised dispersed and infiltrated neurons with morphological alteration including karyopyknosis which resulted in notable reduction (*p* < 0.05) in neuronal counts in DG and CA1 regions of hippocampi, as compared to healthy brains. The mice supplemented with Erqember showed protection against AlCl_3_-induced histopathological changes in a dose-dependent manner and neuronal integrity was noted to be preserved by tested polyherbal preparation. The mice consuming Erq-Em 10 had increased neuronal count in DG region (*p* < 0.05) while Erq-Em 20 caused neuronal protection as granular cell layer of DG (*p* < 0.01) (Figure 6(a)) and healthy pyramidal cells in CA1 (*p* < 0.01) (Figure 6(b)) regions were noticeably increased, as compared to amnesic control mice.

### 3.7. BBB Permeability and Molecular Docking Studies

**Figure 7:**
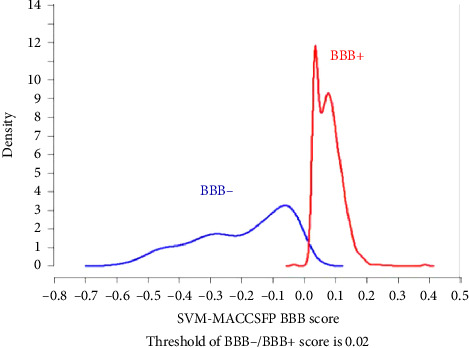
Graphical representation of standard threshold value for BBB− and BBB+.

Figure 7 graphically represents the threshold value of BBB+ (permeability) and BBB− (not permeable) based on SVM and LiCABEDS algorithms. The 0.02 is the threshold value for the BBB+ and BBB−. The ligands with SVM_MACCSFB score less than 0.02 were not able to cross BBB, while the ligands with SVM_MACCSFB score greater than 0.02 were considered to have good BBB permeability. The 2-D diagrams with the SVM_MACCSFB score of all ligands are depicted in Table 1. The Lathyrine, Scandenin, and Pyropheophorbide were not supposed to cross the BBB with SVM_MACCSFB scores of −0.029, −0.092, and 0.018, respectively, whereas Dictyoquinazol C and Chlorovulone III were considered to have the capability to cross the BBB with SVM_MACCSFB scores of 0.051 and 0.088, respectively.

**Figure 8:**
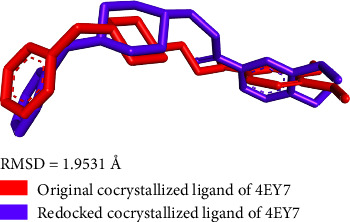
RMSD value of cocrystallized ligand.

The co-crystallized ligand was redocked via AutoDockTools-1.5.7 software to compute the value of the root-mean-square deviation (RMSD). RMSD is a commonly used parameter to determine the accuracy and validity molecular docking predicted values. RMSD value of **< 2 Å i**ndicates that the docked ligand closely resembles the co-crystallized ligand. It highlights the validated docking prediction. The calculated RMSD value (1.9531 Å) as presented in Figure 8 is well aligned with the accepted literature values (< 2 Å), indicating that the structure of the docked co-crystallized ligand is overlapped to such a large extent with the structure of the original co-crystallized ligand, thus the docking procedure highly simulates the ligand–target protein interaction.

In this study, all ligands were docked by using the coordinates identified from the structure of the protein that contains the co-crystallized ligand. The outcomes of the docking analysis were evaluated based on the glide score listed in Table 2. The docking poses and binding interaction of ligands towards the binding pocket of the receptor were visually inspected by using 3D and 2D interaction diagrams of all ligands. The docking scores are exhibited in negative values. The more negative value of the docking score and glide energy, the greater the binding affinity of the ligand to the receptor, and vice versa.

**Figure 9:**
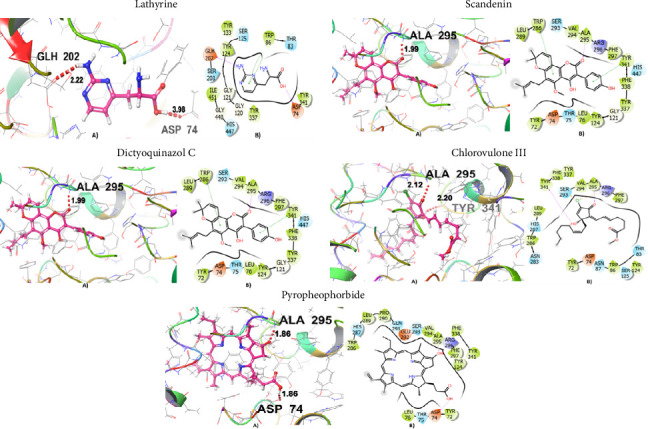
3D and 2D visualization of the interaction of compounds with target protein.

The Lathyrine has glide score −5.427 with polar residues THR83, SER125, SER204, and HIS447; hydrophobic residues TRP86, TRP124, TRP133, TYR314, TYR337, and ILE451; while the ASP74 and GLH202 were hydrogen bond interacting residues with distance 3.98 and 2.22 Å, respectively. The Scandenin was considered the 2nd highest-ranked compound with a glide score of 8.319. The ALA295 was hydrogen bond interacting residue; THR75, SER293, and HIS 447 showed polar interaction; and TYR72, LEU76, TYR124, TRP286, LEU289, VAL294, ALA295, PHE297, TYR341, TYR337, and TYR338 showed hydrophobic interaction to the Scandenin. The Dictyoquinazol C displayed the highest binding affinity among all the ligands with a glide score of −8.612 among all the examined compounds. The ALA295 and TYR318 were the amino acid residues involved in hydrogen bonding with a distance of 2.63 Å. SER293 and HIS447 were the polar interacting amino acid residues, while TYR72, TYR124, TRP286, LEU289, VAL294, ALA295, PHE297, THR318, TYR337, and TYR338 were the hydrophobic interacting residues for the Dictyoquinazol C ligand. The Chlorovulone III have glide score −7.364 with hydrogen interacting amino acid residues ALA95 and TYR341 through distance 1.86 Å; polar interacting residues THR83, ASA87, SER125, ASN283, HIS287, and SER293; and hydrophobic interacting residues TYR72, TRP86, TRP124, TRP286, LEU289, VAL294, ALA295, PHE297, TYR341, TYR337, and PHE338. The Pyropheophorbide also represented good binding affinity with a glide score of −7.55. The ASP74 and ALA294 amino acid residues showed hydrogen bond interaction through a distance of 1.86 Å. The residues THR75, HIS287, GLN291, and SER293 showed polar interaction, while the residues TYR72, LEU76, TYR124, TRP286, LEU289, PRO290, VAL294, ALA295, PHE297, TYR341, and PHE338 showed hydrophobic interaction represented in Figure 9 and Table 2.

### 3.8. ADMET Study

The goal of this study is to focus on assessing the ADMET properties of the ligands that depicted the favorable binding interactions with the target protein in glide molecular docking analysis. The ADMET study was performed by the admetSAR 3.0 web tool.  Table 3 displays the ADMET of all investigated ligands.

It was reported that all ligands exhibited good absorption when the value of total polar surface area (TPSA) was lower than 140 Å^2^. All ligands in this study showed good absorption by compliance with this standard value. All ligands showed moderate to low capability to cross the BBB except Pyropheophorbide according to the optimum range. According to admetSAR, the optimum range for the drug to cross the BBB is reported into three categories: high, moderate, and low falling in the ranges of 0–0.3, 0.3–0.7, and 0.7–1, respectively. It is reported that the ligand with a value greater than 1 should not cross the BBB. Table 2 shows that Lathyrine has good water solubility and significantly inhibits the permeability glycoprotein (P-gp), correspondingly its effectiveness does not interact.

ADMET studies of all compounds were also carried out by ADMET boost https://ai-druglab.smu.edu/admetresult. The ADMET parameters obtained from this web tool are listed in Table 4. The ligands Scandenin and Pyropheophorbide represented a high ability to cross the BBB due to values less than 30%, while all other ligands showed moderate ability to cross the BBB having values in the range between 30% and 70%. The ligand with a value in the range between 70% and 100% showed poor ability to cross BBB. Moreover, all the ligands have no more than 5 hydrogen bond donors and no more than 10 hydrogen bond acceptors, and their corresponding molecular weight is less than 600 Da depicting them as suitable drug candidates.

## 4. Discussion

The true therapeutic cure is deficient and clinically used medicinal options prove insufficient to halt the development and progression of AD. The complications associated with drug development and clinical trials for AD are challenging. The factors including imprecision, partiality, absence of operational protocols, and lack of homogeneous sampling during clinical trials have led to poor advancement in drug development [37]. Moreover, AD causes psychological changes such as anxiety and compromised cognitive abilities suggesting some disruption to subcortical structures [38]. The therapeutic remedies employed for AD are anticholinesterases which inhibit acetylcholinesterase enzymes and increase cholinergic neurotransmission to bring symptomatic relief in patients without curing the disease progression [39].

Neuroscientists have been attempting to search the multimodal approaches capable of countering pathological events to reduce the suffering of patients with AD. [40]. Hence, in recent years, the demand to look for alternative treatments is rising, particularly the polyherbal preparations are gaining momentum. The polyherbal preparations possessing phytochemicals capable of crossing the BBB work through multifaceted mechanisms to slow the disease progression and manage its symptoms [41]. Keeping these points in view, the present study aimed to scientifically evaluate the potential of the commercially available and widely used polyherbal product Erqember to counter the neurotoxic effects of AlCl_3_ in experimental mice model.

Anxiety is a neurobehavioral state that results from distress of perceived intimidations. In the current study, AlCl_3_ exposure caused anxiety-like behavior in mice and they spent more time in peripheral, dark, and hidden zones of experimental mazes. These findings are supported by a previous study in which AlCl_3_-exposed mice showed anxiety in OFT, L/D, and EPM tests, and authors reported that exposure of mice to AlCl_3_ promotes neurodegenerative factors associated with anxiety-like behaviors as mice showed [42]. In another study, Buraimoh et al. aimed to evaluate the impact of AlCl_3_ on anxiety in rats and noted negative effects of AlCl_3_ on anxiety-like behavior as rats spent more time in the hidden arms of EPM [43]. The mice supplemented with Erqember showed dose-dependent protection from the anxiogenic effect of AlCl_3_ and they notably explored anxiogenic exposed areas of the maze in all tests.

The BBB permeability is a crucial parameter to be considered in the development of drugs affecting the brain. Hence, the current study included the in silico approaches to predict the BBB permeability and ADMET studies revealed that all compounds had the capability to cross BBB except Pyropheophorbide. The literature reports that exposure to AlCl_3_ is associated with impaired cholinergic transmission in the brain which leads to cognitive incapacities [6]. To predict the interaction of detected compounds with acetylcholinesterase protein, all compounds were docked and the glide score revealed that Dictyoquinazol C had the highest binding affinity. Further, Scandenin, Pyropheophorbide, and Chlorovulone III also showed appreciable binding affinities while Lathyrine had the least among all. Thus, the improved cognition in mice consuming Erqember during Y-maze, NOR, passive avoidance, and water maze tests might be attributed to the detected compounds of Erqember as they might be interacting with and inhibiting acetylcholinesterase leading to elevated levels of synaptic acetylcholine.

The brain is a potential target for aluminum toxicity, after readily crossing the BBB it accumulates in frontal cortex and hippocampus of brain [44]. The evidence from preclinical and epidemiological studies reveals that the deposition of aluminum in these regions of the brain precipitates neuroinflammation and neuronal necrosis [45]. Since neuroinflammation is one of the major factors playing a crucial role in the pathogenesis of neurodegenerative disorders, aluminum accretion causes impairment in learning and memory [46]. Scandenin, present in Erqember, is an anti-inflammatory isoflavonoid that has been reported to inhibit the enzymes involved in the synthesis of eicosanoids [47]. The UHPLC-MS analysis of Erqember revealed the presence of Chlorovulone III which belongs to the eicosanoids class of compounds. In a previous study, *Funaria hygrometrica* extract reduced inflammation in carrageenan-induced paw edema and these outcomes were attributed to the phytochemicals present in plants including Chlorovulone III [48]. Pyropheophorbide was also detected in Erqember, and a previous study attributed the anti-inflammatory potential of *Saccharina japonica* to its compounds pheophorbide a and pheophytin which showed the potential to inhibit the lipopolysaccharide-induced production of nitric oxide by inhibiting the iNOS protein expression [49].

The higher demands of oxygen, abundant polyunsaturated fatty acids content, and insufficient antioxidative defenses make the brain highly vulnerable to oxidative damage [50]. In our study, the exposure of mice to aluminum-induced oxidative stress in mice and these findings are supported by Rui and Yongjian who administered AlCl_3_ and noted elevated levels of MDA and SOD in the hippocampus and cortex of sacrificed mice [51]. This elevated oxidative stress also contributes to deteriorating cognitive abilities [52], and this might be the reason behind impaired cognition in AlCl_3_-treated control mice. The mice supplemented with Erqember had reduced levels of MDA and elevated activity of antioxidative SOD and GPx enzymes. Scandenin has been previously reported to own radical scavenging potential as phytochemical analysis of *Derris scandens* led to the isolation of Scandenin A and Scandenin B, which showed radical scavenging properties in DPPH assay [53]. In a study by Lee et al., the neuroprotective effects of Dictyoquinazol C were established by claiming that Dictyoquinazol C holds the potential to protect cortical neurons of mice from excitotoxicity and cell death induced by glutamate and NMDA [54]. Metabolic disturbances resulting from neuronal excitotoxicity may increase the generation of reactive oxygen species leading to a pro-oxidant condition [55]. Dictyoquinazol C might regulate neuronal homeostasis through its anti-excitatory and antioxidant potential.

## 5. Conclusion

The present study provides scientific insights into the neurological benefits of Erqember. The nutritional supplementation of mice with this polyherbal formulation protected them from AlCl_3_-induced neurobehavioral changes in terms of anxiety-like behavior and cognition. Further, the neurotoxic effects of AlCl_3_ leading to oxidative stress in the brain and histopathological alterations in of hippocampus were markedly protected by Erqember. The reduced acetylcholinesterase activity and elevated antioxidant levels noted in isolated brains suggested that phytocompounds owned by Erqember might be playing the observed beneficial effects on brain health by improving cholinergic neurotransmission and reducing oxidative stress.

## Figures and Tables

**Table 1:** 2-D representation with their SVM_MACCSFB BBB score retrieved from https://www.cbligand.org/BBB/predictor.php.

Ligands	2-D structures of ligands	SVM_MACCSFB BBB score	Interpretation (SVM_MACCSFB)
Lathyrine	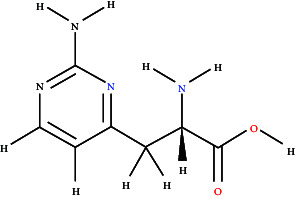	−0.029	BBB−

Scandenin	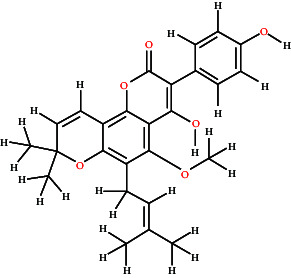	−0.092	BBB−

Dictyoquinazol C	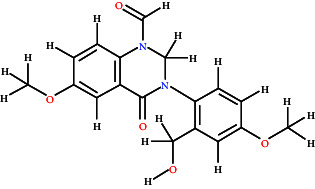	0.051	BBB+

Chlorovulone III	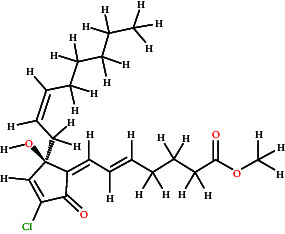	0.088	BBB+

Pyropheophorbide	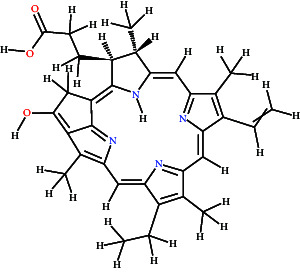	0.018	BBB−

**Table 2:** Glide score, emodel score, hydrogen, polar, and hydrophobic bond interacting residues of all ligands.

Ligands	Docking score	Glide score	Glide emodel	Hydrogen bond interacting residues	Polar bond interacting residues	Hydrophobic bond interacting residues
Lathyrine	−5.427	−5.427	−44.743	ASP74 GLH202	THR83 SER125 SER204 HIS447	TRP86, TRP124, TRP133, TYR314, TYR337, ILE451

Scandenin	−8.319	−8.319	−69.026	ALA295	THR75 SER293 HIS 447	TYR72, LEU76, TYR124, TRP286, LEU289, VAL294, ALA295, PHE297, TYR341, TYR337, TYR338

Dictyoquinazol C	−8.612	−8.612	−66.454	ALA295 TYR318	SER293 HIS447	TYR72, TYR124, TRP286, LEU289, VAL294, ALA295, PHE297, THR318, TYR337, TYR338

Chlorovulone III	−7.364	−7.364	−62.195	ALA95 TYR341	THR83 ASA87 SER125 ASN283 HIS287 SER293	TYR72, TRP86, TRP124, TRP286, LEU289, VAL294, ALA295, PHE297, TYR341, TYR337, PHE338

Pyropheophorbide	−7.552	−7.552	−52.19	ASP74 ALA294	THR75 HIS287 GLN291 SER293	TYR72, LEU76, TYR124, TRP286, LEU289, PRO290, VAL294, ALA295, PHE297, TYR341, PHE338

**Table 3:** ADMET analysis (TPSA, solubility, BBB, P-gp inhibitor, half-life, and cytochromes p450 inhibitors).

Ligands	TPSA	logS	BBB	P-gp inhib	CYP1A2 inhib	CYP3A4 inhib	CYP2B6 inhib	CYP2C9 inhib	CYP2C19 inhib	CYP2D6 inhib	T1/2
Lathyrine	115.1	0.06	0.36	0.01	0.02	0.01	0.05	0.02	0.027	0.018	−0.57
Scandenin	89.1	−5.67	0.54	0.59	0.54	0.06	0.70	0.66	0.377	0.08	−0.95
Dictyoquinazol C	79.3	−3.05	0.82	0.36	0.68	0.51	0.12	0.38	0.578	0.147	0.05
Chlorovulone III	63.6	−6.27	0.58	0.94	0.19	0.07	0.49	0.24	0.199	0.121	−0.18
Pyropheophorbide	106.6	−6.19	0.19	0.90	0.30	0.03	0.56	0.16	0.126	0.054	−2.02

**Table 4:** The ADMET parameters of all compounds.

Molecule property	Lathyrine	Scandenin	Dictyoquinazol C	Chlorovulone III	Pyropheophorbide
Molecular weight	182.08	434.17	342.12	380.18	534.26
Number of heteroatoms	6	6	7	5	7
Number of rotatable bonds	3	4	5	11	5
Number of rings	1	4	3	1	6
Number of HA	5	6	5	4	6
Number of HD	3	2	1	1	3
Log KOW	−0.99	5.57	1.78	4.78	6.71
Caco-2 permeability	−5.19	−5.16	−5.13	−5.17	−5.46
HIA	73.92	73.68	71.15	67.96	68.43
P-gp inhibition	28.52	52.25	45.36	41.52	49.95
Log D7.4	1.25	1.8	2.02	1.97	1.72
Aqueous solubility	−4.59	−4.18	−4.29	−4.84	−4.03
Oral bioavailability	52.31	40.43	45.16	36.07	42.61
BBB	34.2%	20.09%	34.68%	39.37%	29.54%
PPBR	43.75	47.84	52.7	60.09	60.3
VDss	2.45	3.71	3.45	3.51	3.58
CYP2C9 inhibition	44.81	65.2	62.39	48.8	43
CYP2D6 inhibition	85.25	95.99	93.84	88.89	108.75
CYP3A4 inhibition	39.43	36.86	39.82	35.6	34.92
Half-life	39.68	62.18	57.37	61.23	132.45
hERG blockers	32.81	41.21	42.08	37.52	43.01
Ames	43.76	49.6	42.28	42.98	43.81
DILI	41.33	51.22	49.28	42.04	58.2
LD50	1.64	2.38	2.34	1.75	3.03

## Data Availability

The data that support the findings of this study are available from the corresponding author upon reasonable request.

## Supporting Information

Supporting File 1: A file containing UHPLC-MS data containing details of identified compounds.
