# Supplementary material for: Erqember Mitigates Neurotoxic Effects of Aluminum Chloride in Mice: Phytochemical Insights With Neurobehavioral and In Silico Approaches
Source: J Toxicol. 2025 Apr 2;2025:3997995. doi: 10.1155/jt/3997995 (PMC11981706; doi:10.1155/jt/3997995)
Supplement: Supporting Information — Additional supporting information can be found online in the Supporting Information section. [file 3997995.f1.pdf]

Qualitative Compound Report

**Data File**	A2-Neg-25_30-1uL.d	**Sample Name**	A2-Neg
**Sample Type**	Sample	**Position**	P2-B6
**Instrument Name**	Instrument 1	**User Name**	
**Acq Method**	U-ESI-E-XDB-C18-NB-MS-20(30)-Neg-Sept2015.m	**Acquired Time**	18/1/2023 1:21:37 PM
**IRM Calibration Status**	Success	**DA Method**	Metabolomics-2019.m
**Comment**			

**Sample Group**		**Info.**	
**Stream Name**	LC 1	**Acquisition SW Version**	6200 series TOF/6500 series Q-TOF B.06.01 (B6172 SP1)

Compound Table

Compound Label	RT	Mass	Name	DB Formula	DB Diff (ppm)	Hits (DB)
Cpd 1: 0.651	0.651	850.3332				
Cpd 2: Tyr Tyr	0.653	344.138	Tyr Tyr	C18 H20 N2 O5	-2.35	2
Cpd 3: 0.653	0.653	218.0597				
Cpd 4: Lathyrine	0.664	182.08	Lathyrine	C7 H10 N4 O2	1.86	5
Cpd 5: 0.669	0.669	228.0876				
Cpd 6: 0.691	0.691	245.0772				
Cpd 7: 5.134	5.134	468.0115				
Cpd 8: 5.134	5.134	424.0207				
Cpd 9: 7.402	7.402	537.9847				
Cpd 10: Mitoxantrone	9.795	444.2022	Mitoxantrone	C22 H28 N4 O6	-2.87	1
Cpd 11: Clozapine-N-Oxide	9.795	342.1251	Clozapine-N-Oxide	C18 H19 Cl N4 O	-1.11	10
Cpd 12: Scandenin	9.796	434.1729	Scandenin	C26 H26 O6	0.16	7
Cpd 13: Idarubicin	9.796	497.1684	Idarubicin	C26 H27 N O9	0.39	7
Cpd 14: 9.889	9.889	152.0483				
Cpd 15: Dictyoquinazol C	10.488	342.1223	Dictyoquinazol C	C18 H18 N2 O5	-2.07	10
Cpd 16: Dictyoquinazol C	10.681	342.1229	Dictyoquinazol C	C18 H18 N2 O5	-3.74	10
Cpd 17: Chlorovulone III	10.681	348.1937	Chlorovulone III	C20 H28 O5	-0.08	10
Cpd 18: Carpelastofuran	10.682	522.2229	Carpelastofuran	C30 H34 O8	4.68	1
Cpd 19: 10.682	10.682	476.2174				
Cpd 20: 10.684	10.684	539.2131				
Cpd 21: Antimycin A (A1 Shown)	11.115	534.2576	Antimycin A (A1 Shown)	C27 H38 N2 O9	0.23	1
Cpd 22: 11.119	11.119	597.2534				
Cpd 23: Dictyoquinazol C	11.124	342.1231	Dictyoquinazol C	C18 H18 N2 O5	-4.41	10
Cpd 24: Ligulatin B	11.302	306.1472	Ligulatin B	C17 H22 O5	-1.4	10
Cpd 25: 11.304	11.304	580.267				
Cpd 26: bipindogenin	11.304	406.2369	bipindogenin	C23 H34 O6	-3.26	4
Cpd 27: Pyropheophorbide a	11.305	534.2614	Pyropheophorbide a	C33 H34 N4 O3	3.18	3
Cpd 28: 11.307	11.307	597.2571				
Cpd 29: 12.265	12.265	180.0797				
Cpd 30: 19.171	19.171	682.2947				

Compound Label	*m/z*	RT	Algorithm	Mass
Cpd 1: 0.651	849.3259	0.651	Find by Molecular Feature	850.3332

Compound Chromatograms

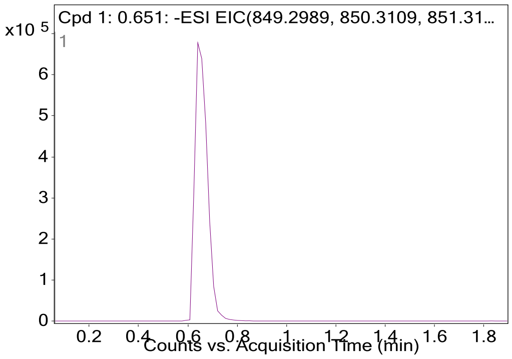

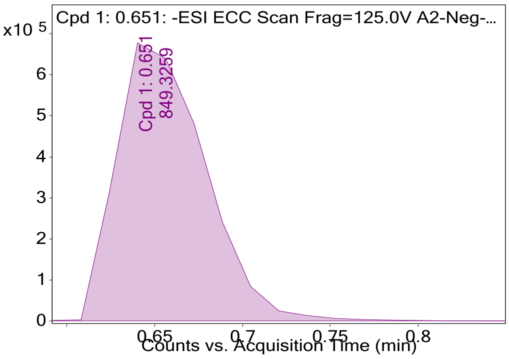

MFE MS Spectrum

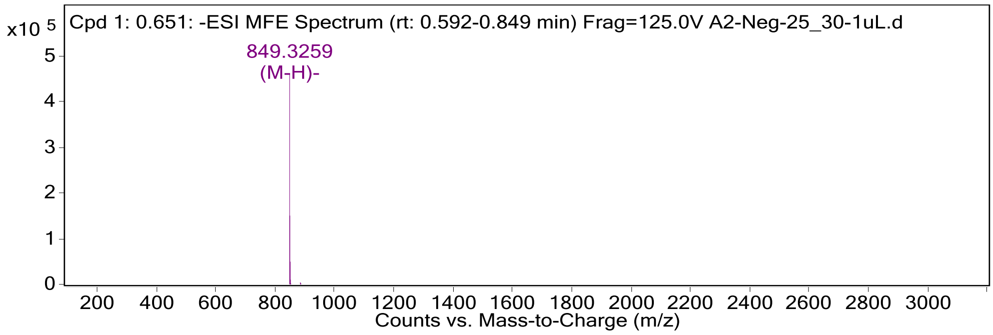

MFE MS Zoomed Spectrum

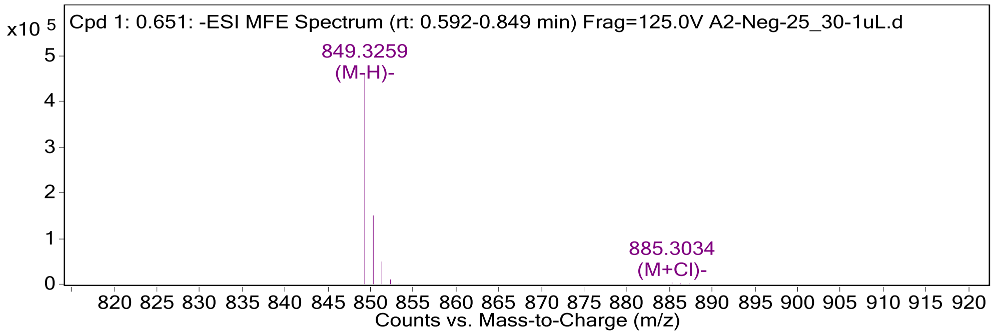

MS Spectrum Peak List

m/z	z	Abund	Ion
849.3259	-1	458352.09	(M-H)-
850.3279	-1	150293.75	(M-H)-
851.3299	-1	49514.97	(M-H)-
852.3318	-1	10264.75	(M-H)-
853.3337	-1	1936.08	(M-H)-
885.3034	-1	4380.09	(M+Cl)-
886.3112	-1	1478.2	(M+Cl)-
887.2984	-1	2518.4	(M+Cl)-

MS Spectrum

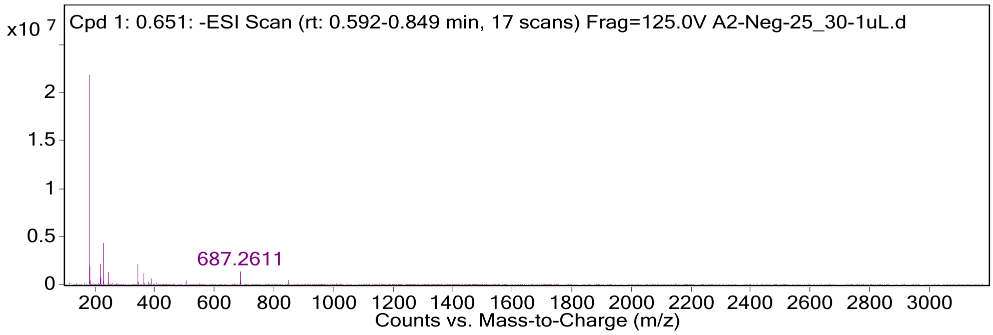

MS Zoomed Spectrum

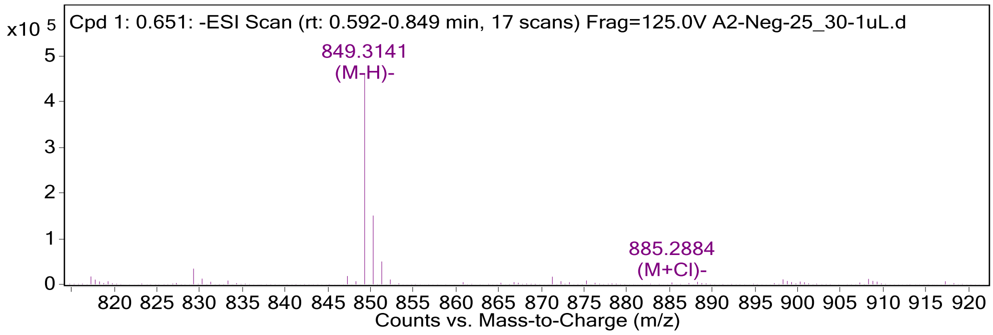

Compound Label	Name	*m/z*	RT	Algorithm	Mass
Cpd 2: Tyr Tyr	**Tyr Tyr**	343.1308	0.653	Find by Molecular Feature	344.138

Compound Chromatograms

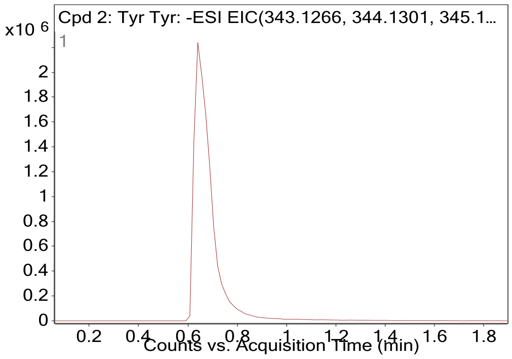

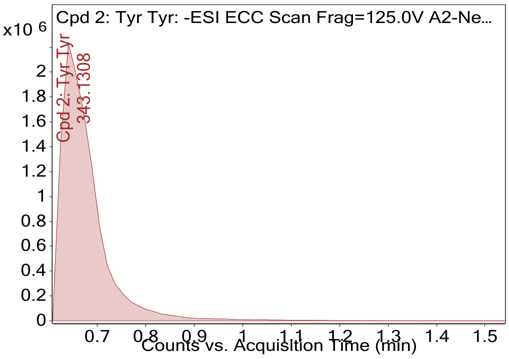

MFE MS Spectrum

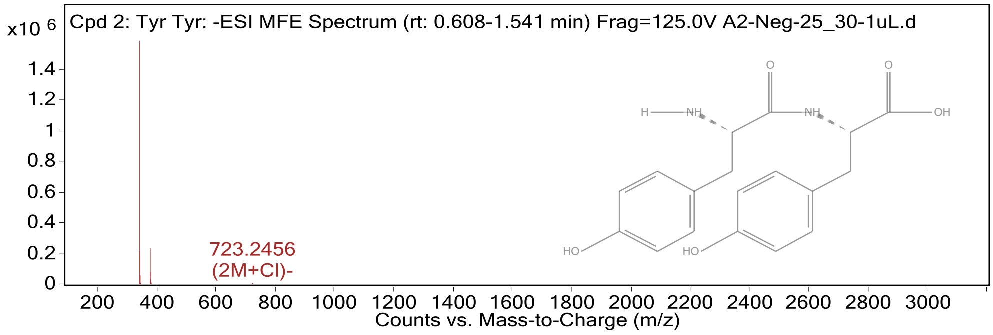

MFE MS Zoomed Spectrum

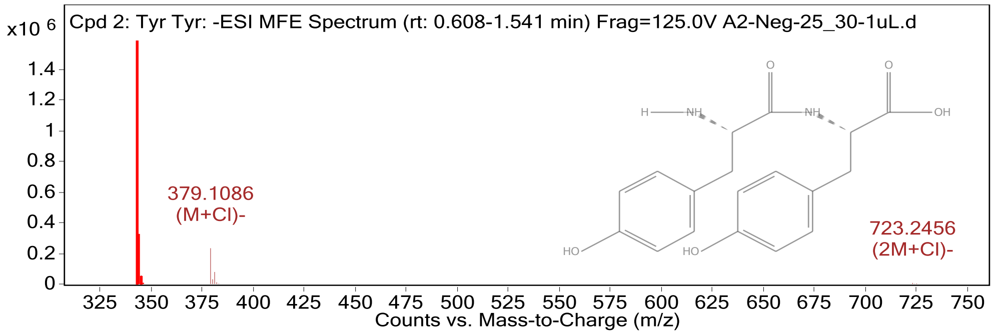

MS Spectrum Peak List

m/z	z	Abund	Formula	Ion
343.1308	-1	1585321.13	C18 H20 N2 O5	(M-H)-
344.134	-1	215453.66	C18 H20 N2 O5	(M-H)-

345.1366	-1	56610.97	C18 H20 N2 O5	(M-H)-
346.1365	-1	7004.13	C18 H20 N2 O5	(M-H)-
379.1086	-1	233532.05		(M+Cl)-
380.112	-1	31145.57		(M+Cl)-
381.1058	-1	78500.97		(M+Cl)-
382.109	-1	11349.24		(M+Cl)-
723.2456	-1	7142.67		(2M+Cl)-
725.2364	-1	3719.41		(2M+Cl)-

MS Spectrum

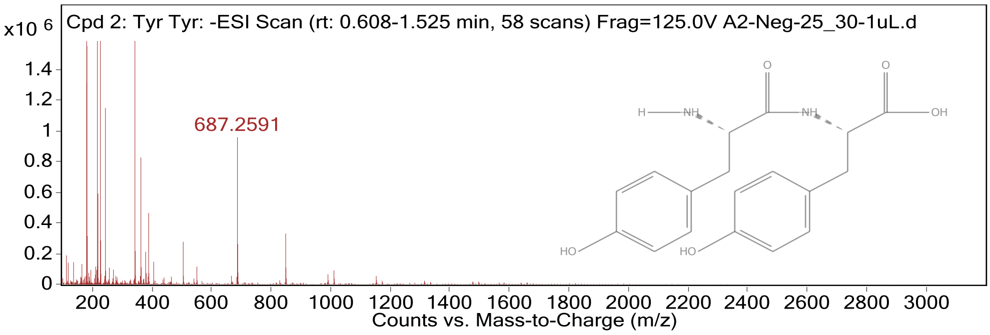

MS Zoomed Spectrum

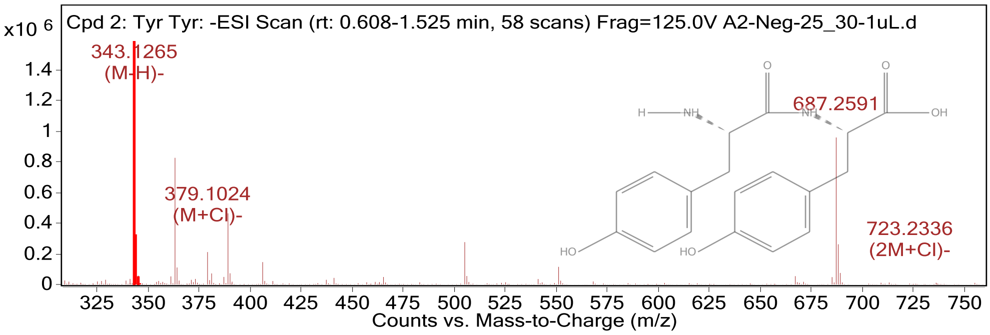

Compound Structure

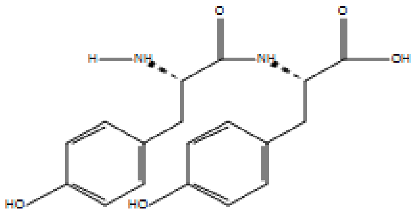

**Compound Label**	***m/z***	**RT**	**Algorithm**	**Mass**
Cpd 3: 0.653	217.0524	0.653	Find by Molecular Feature	218.0597

Compound Chromatograms

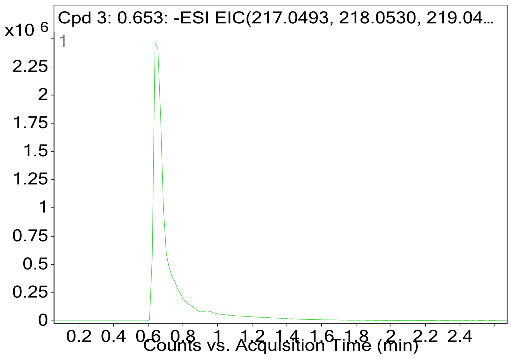

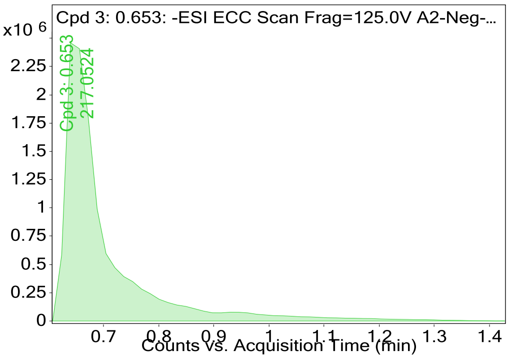

MFE MS Spectrum

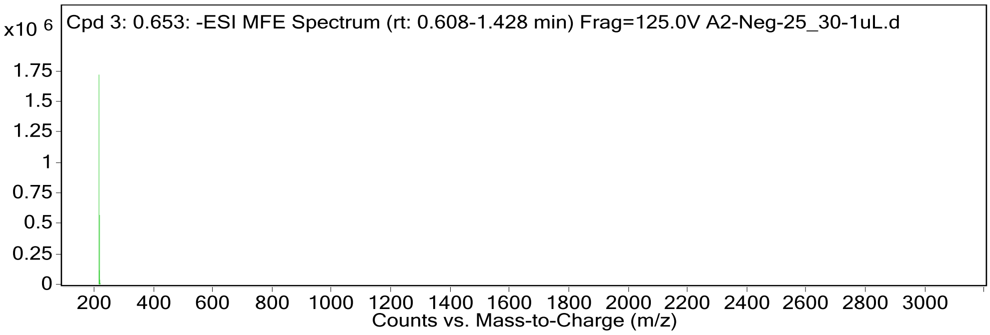

MFE MS Zoomed Spectrum

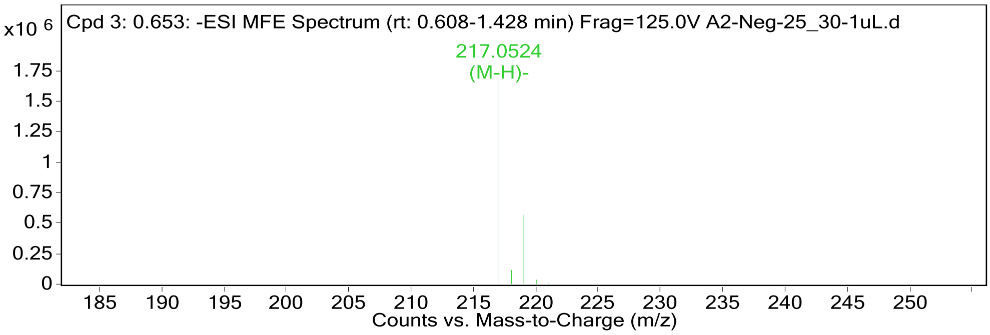

MS Spectrum Peak List

m/z	z	Abund	Ion
217.0524	-1	1717870.25	(M-H)-
218.0554	-1	115809.49	(M-H)-
219.0498	-1	568132.44	(M-H)-
220.0527	-1	37244.54	(M-H)-
221.0558	-1	10270.25	(M-H)-

MS Spectrum

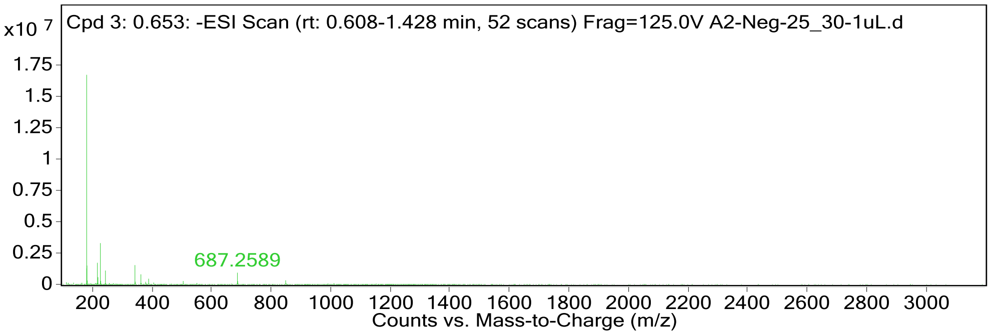

MS Zoomed Spectrum

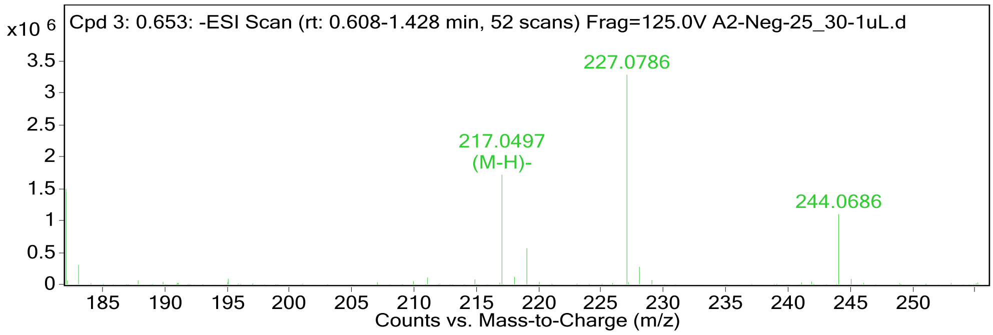

Compound Label	Name	*m/z*	RT	Algorithm	Mass
Cpd 4: Lathyrine	**Lathyrine**	181.0726	0.664	Find by Molecular Feature	182.08

Compound Chromatograms

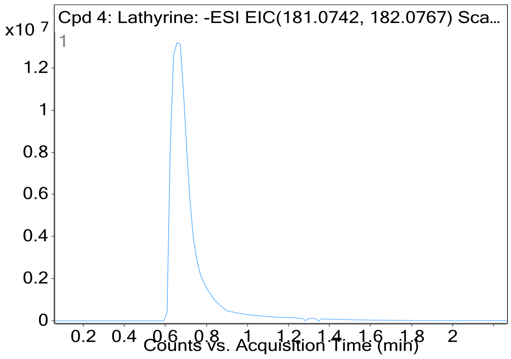

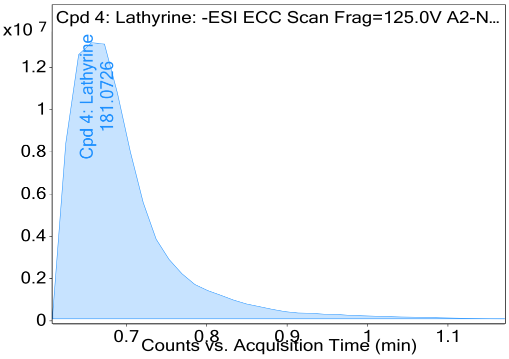

MFE MS Spectrum

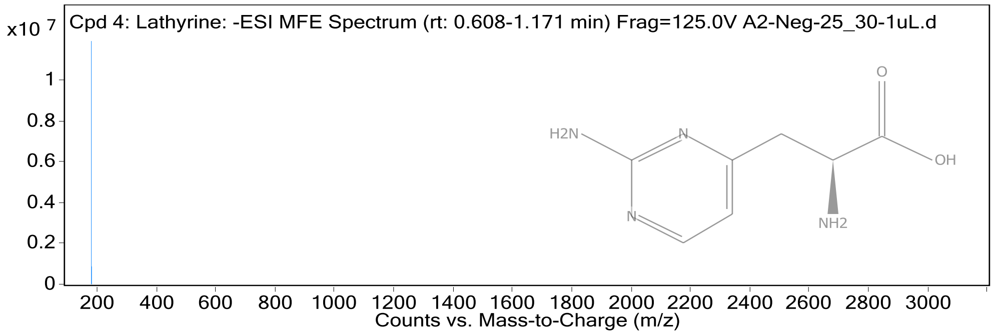

MFE MS Zoomed Spectrum

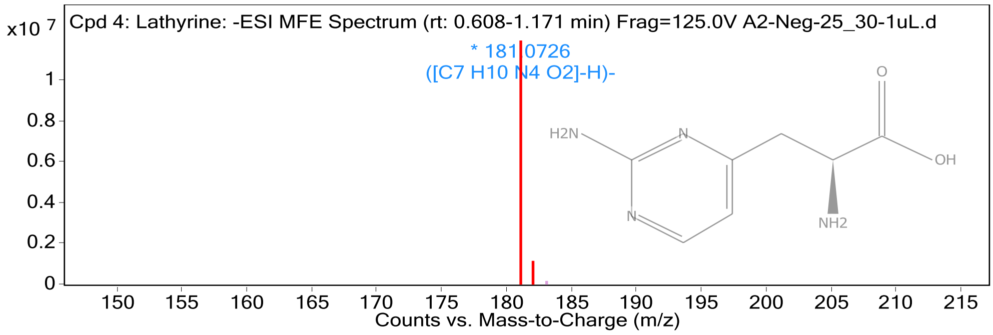

MS Spectrum Peak List

m/z	z	Abund	Formula	Ion
181.0726	-1	11920783	C7 H10 N4 O2	(M-H)-
182.078	-1	873298.18	C7 H10 N4 O2	(M-H)-

MS Spectrum

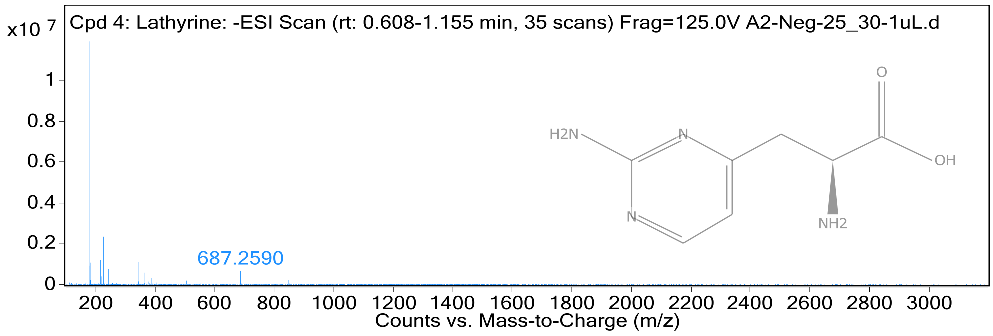

MS Zoomed Spectrum

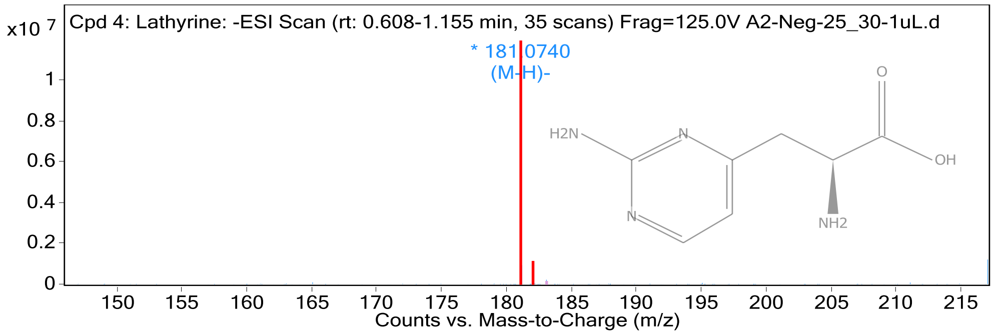

Compound Structure

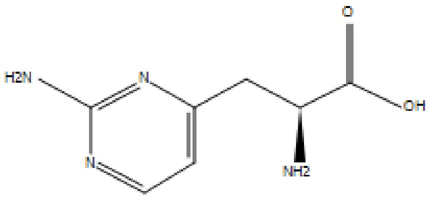

**Compound Label**	***m/z***	**RT**	**Algorithm**	**Mass**
Cpd 5: 0.669	227.0804	0.669	Find by Molecular Feature	228.0876

Compound Chromatograms

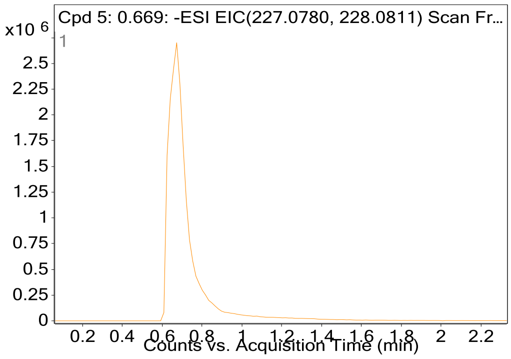

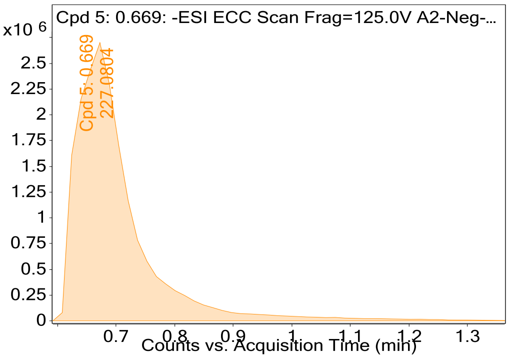

MFE MS Spectrum

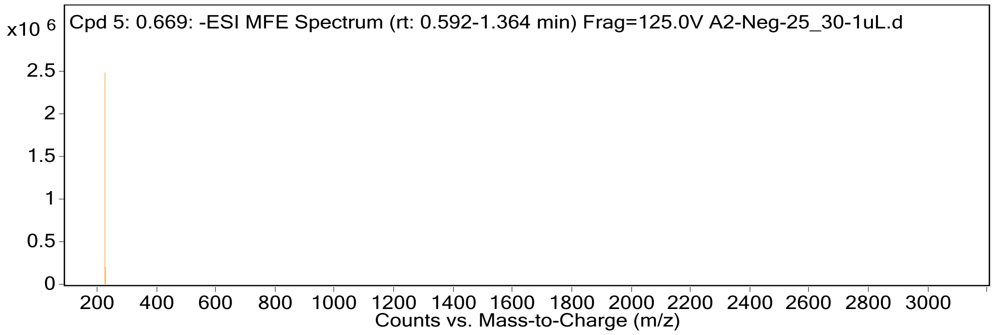

MFE MS Zoomed Spectrum

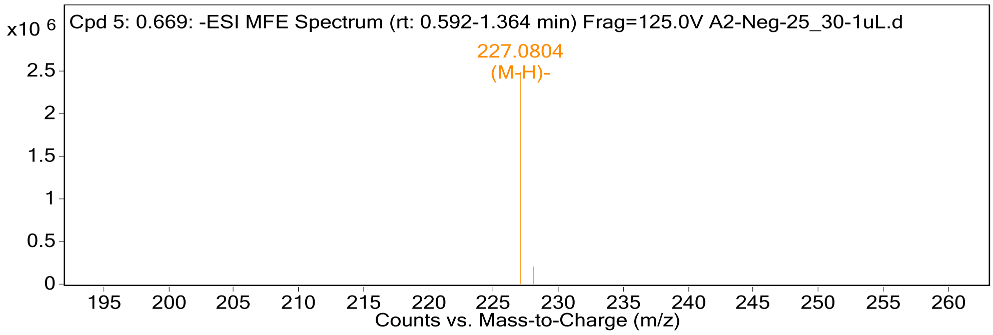

MS Spectrum Peak List

m/z	z	Abund	Ion
227.0804	-1	2488366.25	(M-H)-
228.0835	-1	206671.98	(M-H)-

MS Spectrum

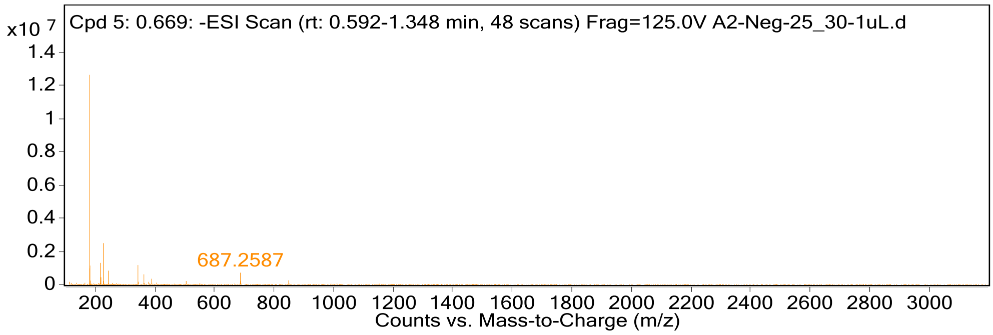

MS Zoomed Spectrum

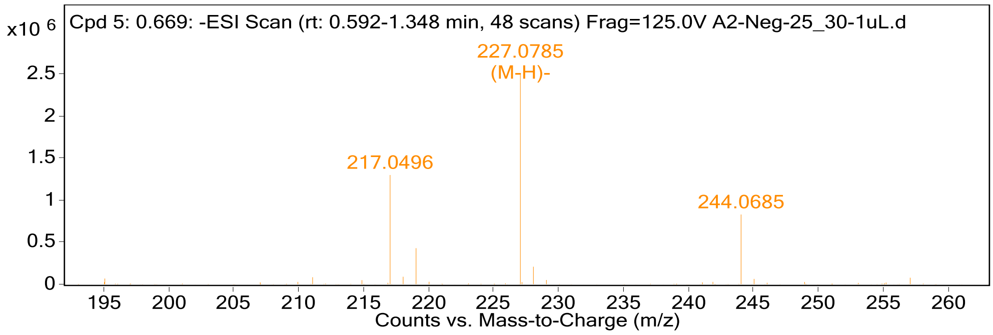

Compound Label	*m/z*	RT	Algorithm	Mass
Cpd 6: 0.691	244.0699	0.691	Find by Molecular Feature	245.0772

Compound Chromatograms

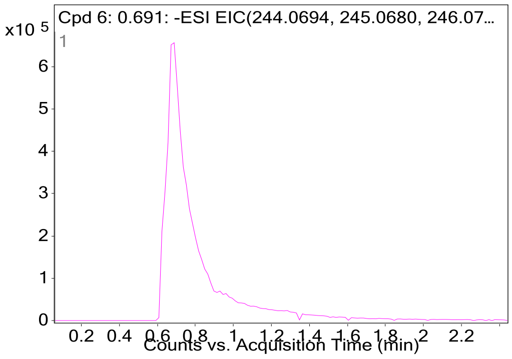

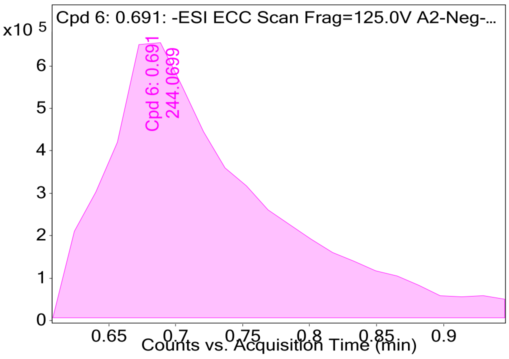

MFE MS Spectrum

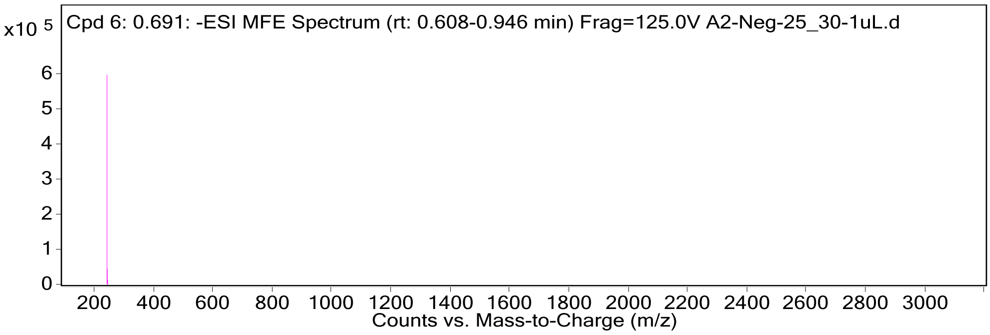

MFE MS Zoomed Spectrum

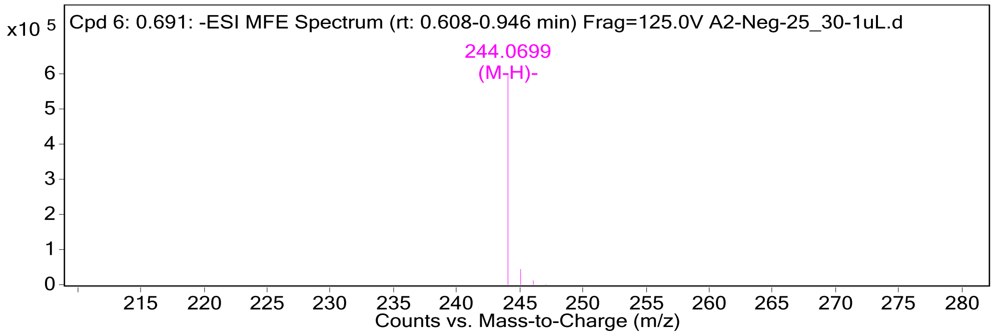

MS Spectrum Peak List

m/z	z	Abund	Ion
244.0699	-1	596980.13	(M-H)-
245.0725	-1	44394.95	(M-H)-
246.0745	-1	12113.58	(M-H)-
247.0707	-1	1552.01	(M-H)-

MS Spectrum

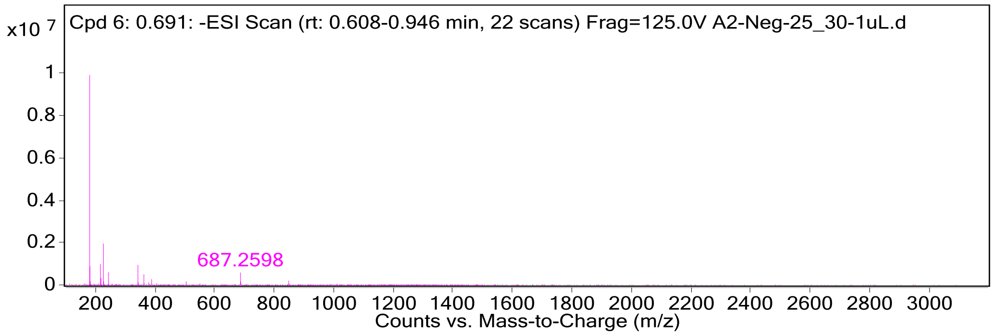

MS Zoomed Spectrum

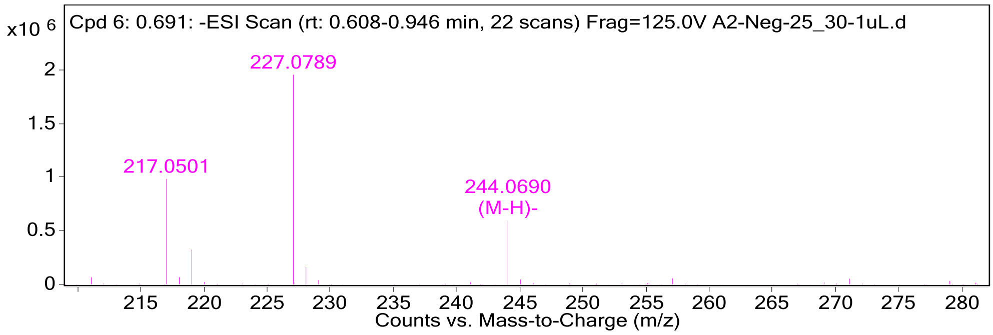

Compound Label	*m/z*	RT	Algorithm	Mass
Cpd 7: 5.134	232.9984	5.134	Find by Molecular Feature	468.0115

Compound Chromatograms

Qualitative Compound Report

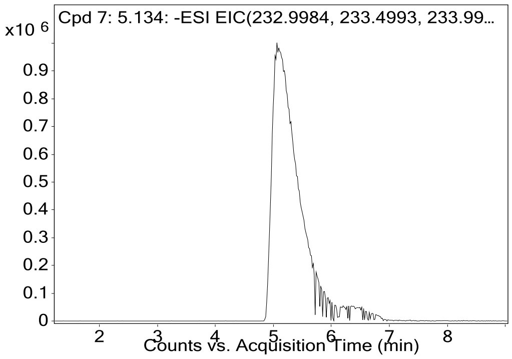

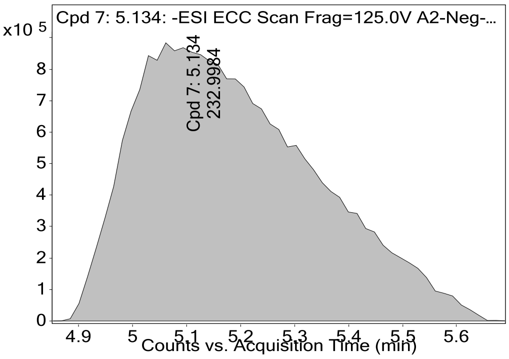

MFE MS Spectrum

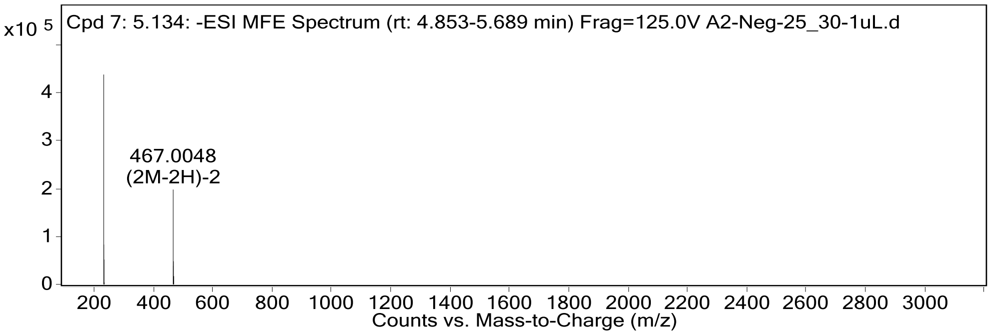

MFE MS Zoomed Spectrum

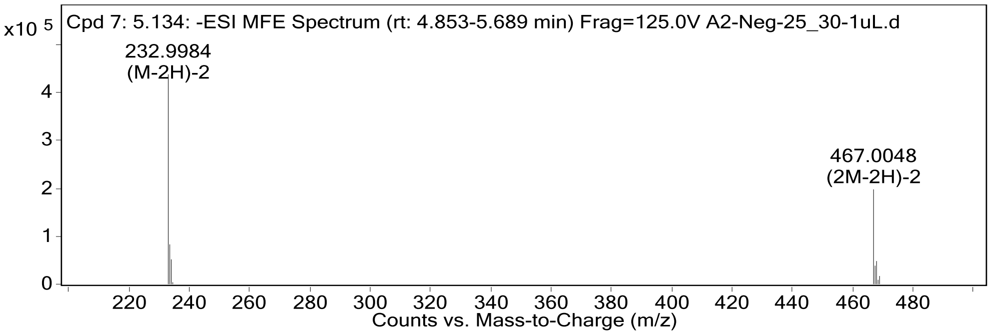

MS Spectrum Peak List

m/z	z	Abund	Ion
232.9984	-2	437986.88	(M-2H)-2
233.4995	-2	83103.67	(M-2H)-2
233.9976	-2	51795.85	(M-2H)-2
234.4985	-2	3999.18	(M-2H)-2
467.0048	-2	197875.3	(2M-2H)-2
467.5066	-2	38572.76	(2M-2H)-2
468.0057	-2	48236.26	(2M-2H)-2
468.5059	-2	9108.79	(2M-2H)-2
469.0029	-2	17053.56	(2M-2H)-2

MS Spectrum

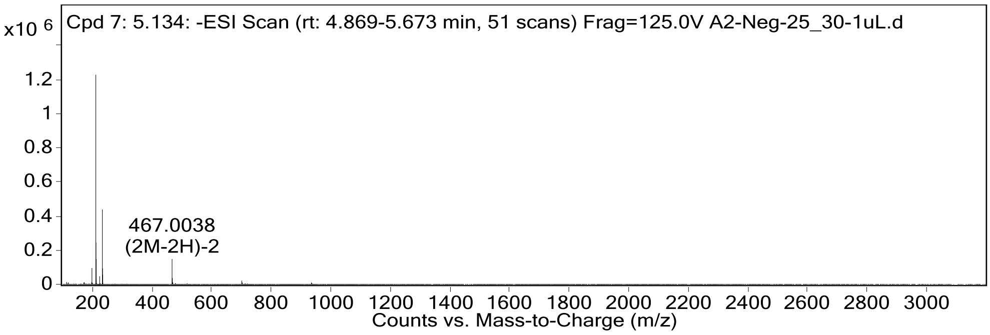

MS Zoomed Spectrum

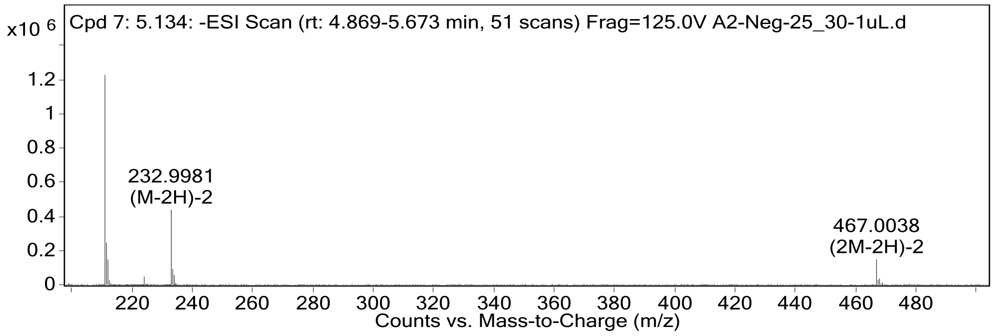

**Compound Label**	***m/z***	**RT**	**Algorithm**	**Mass**
Cpd 8: 5.134	211.0031	5.134	Find by Molecular Feature	424.0207

Compound Chromatograms

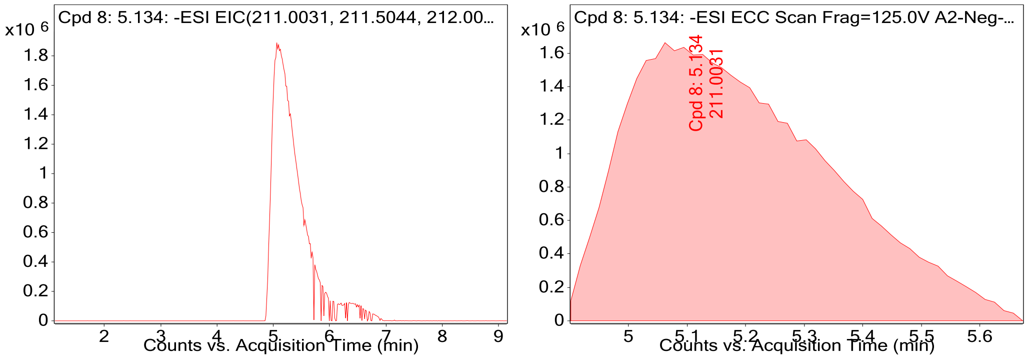

MFE MS Spectrum

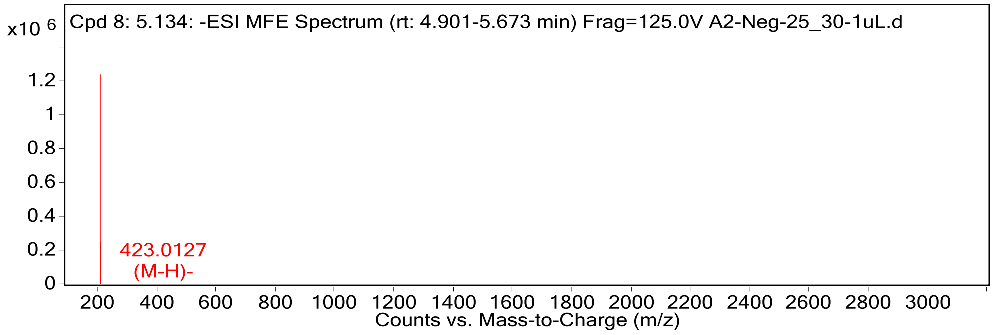

MFE MS Zoomed Spectrum

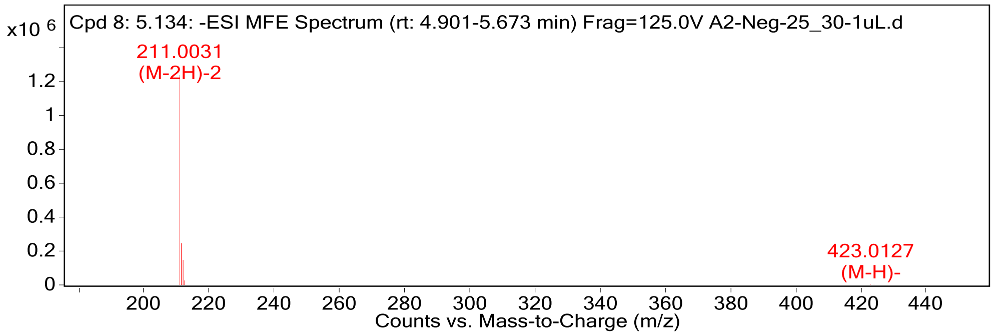

MS Spectrum Peak List

m/z	**z**	**Abund**	**Ion**
211.0031	-2	1239718.13	(M-2H)-2

211.5043	-2	246768.46	(M-2H)-2
212.0021	-2	147471.79	(M-2H)-2
212.5029	-2	26517.32	(M-2H)-2
423.0127	-1	1646.95	(M-H)-
424.0165	-1	410.89	(M-H)-

MS Spectrum

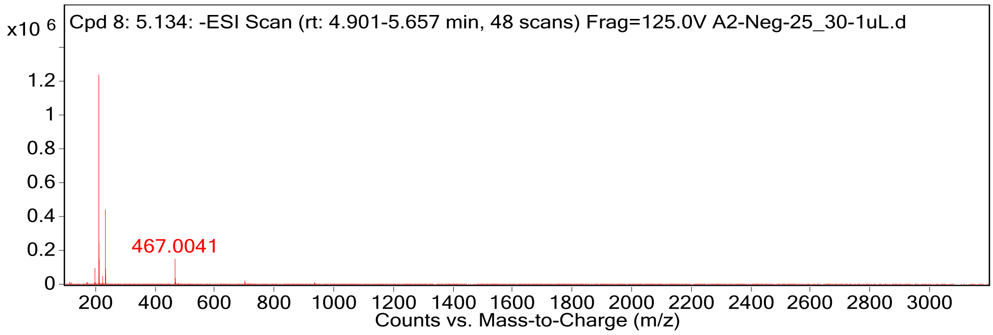

MS Zoomed Spectrum

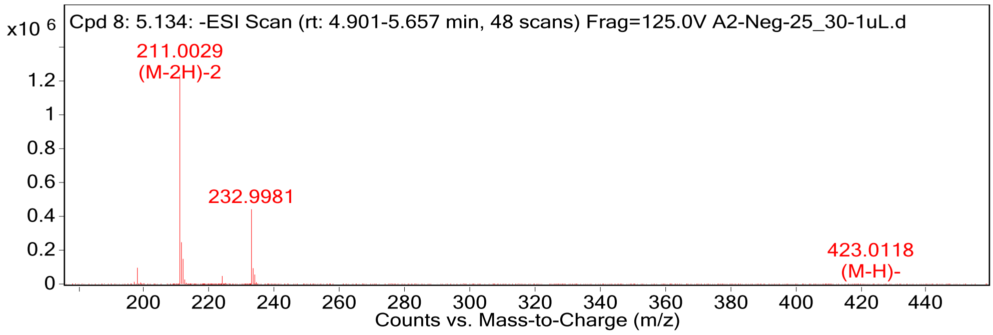

Compound Label	*m/z*	RT	Algorithm	Mass
Cpd 9: 7.402	267.9851	7.402	Find by Molecular Feature	537.9847

Compound Chromatograms

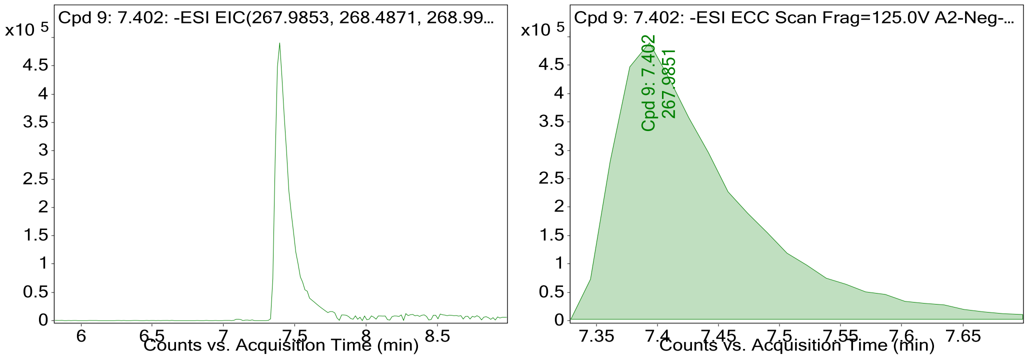

MFE MS Spectrum

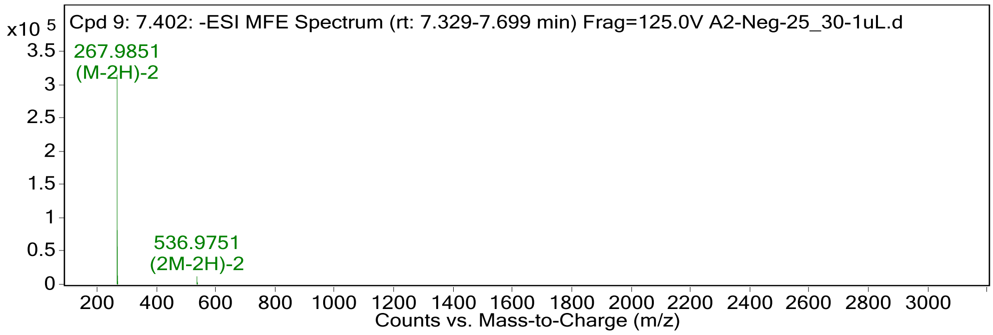

MFE MS Zoomed Spectrum

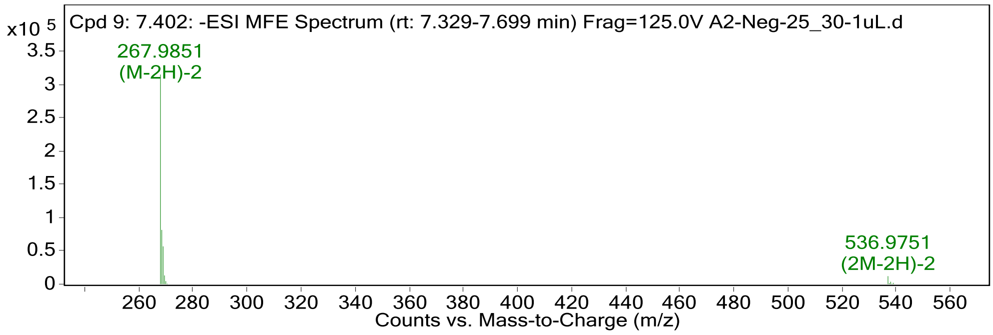

MS Spectrum Peak List

m/z	z	Abund	Ion
267.9851	-2	315314.59	(M-2H)-2
268.4865	-2	81559.39	(M-2H)-2
268.9843	-2	56830.16	(M-2H)-2
269.4853	-2	13136.47	(M-2H)-2
269.9847	-2	4439.95	(M-2H)-2
270.4847	-2	744.4	(M-2H)-2
536.9751	-2	12012.55	(2M-2H)-2
537.48	-2	1736.43	(2M-2H)-2
537.9779	-2	3579.46	(2M-2H)-2
538.9756	-2	1925.02	(2M-2H)-2

MS Spectrum

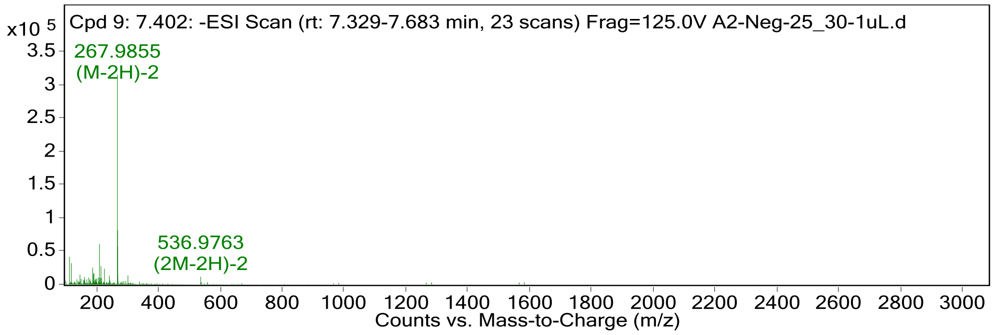

MS Zoomed Spectrum

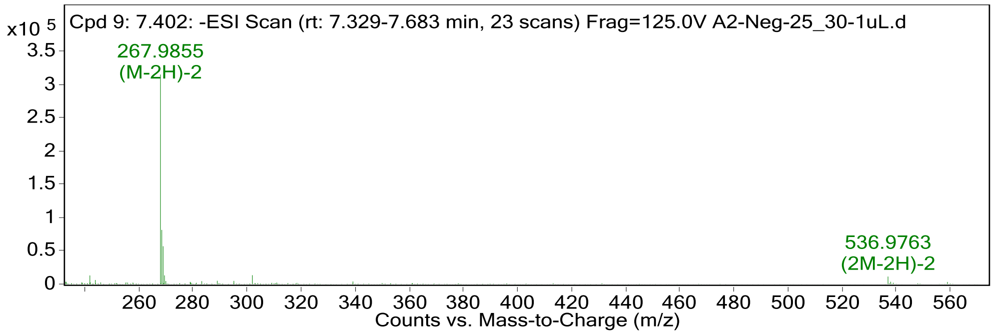

Qualitative Compound Report

Compound Label	Name	*m/z*	RT	Algorithm	Mass
Cpd 10: Mitoxantrone	**Mitoxantrone**	479.1714	9.795	Find by Molecular Feature	444.2022

Compound Chromatograms

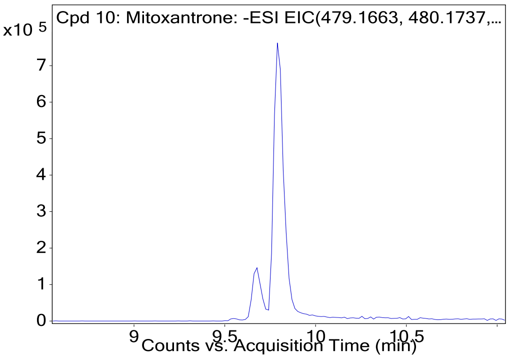

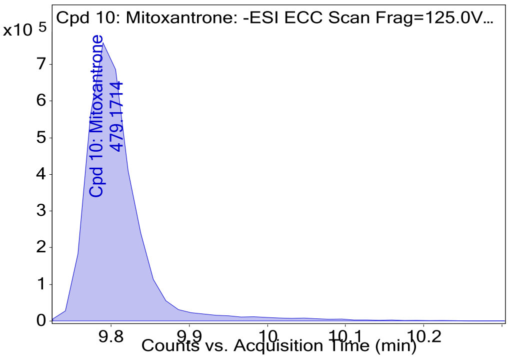

MFE MS Spectrum

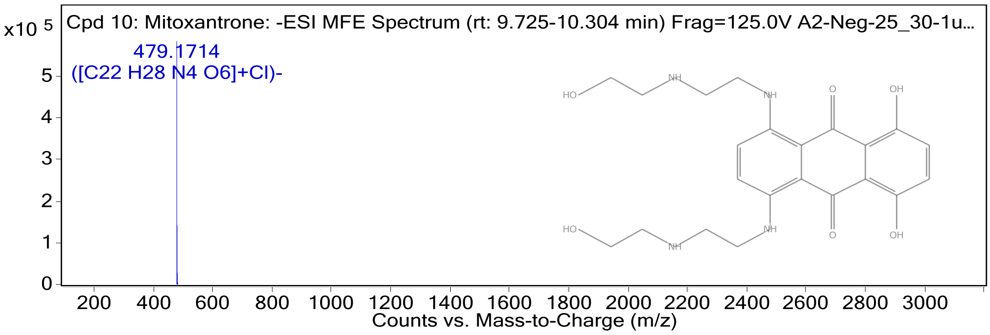

MFE MS Zoomed Spectrum

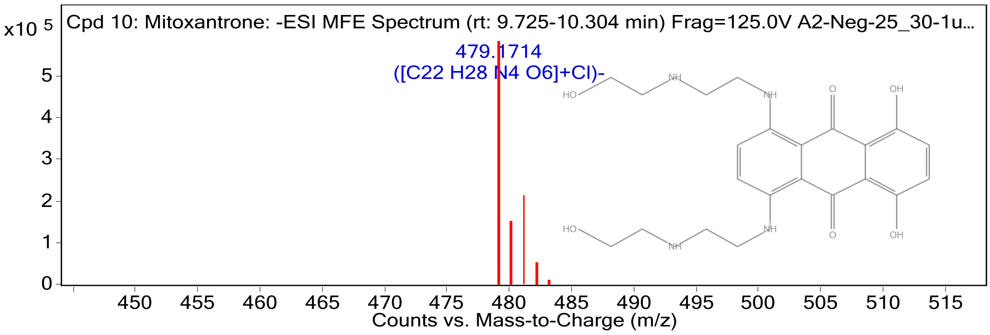

MS Spectrum Peak List

m/z	z	Abund	Formula	Ion
479.1714	-1	583523.63	C22 H28 N4 O6	(M+Cl)-
480.1741	-1	142340.48	C22 H28 N4 O6	(M+Cl)-
481.1758	-1	27783.24	C22 H28 N4 O6	(M+Cl)-
482.1764	-1	4428.75	C22 H28 N4 O6	(M+Cl)-
483.1766	-1	759.05	C22 H28 N4 O6	(M+Cl)-

MS Spectrum

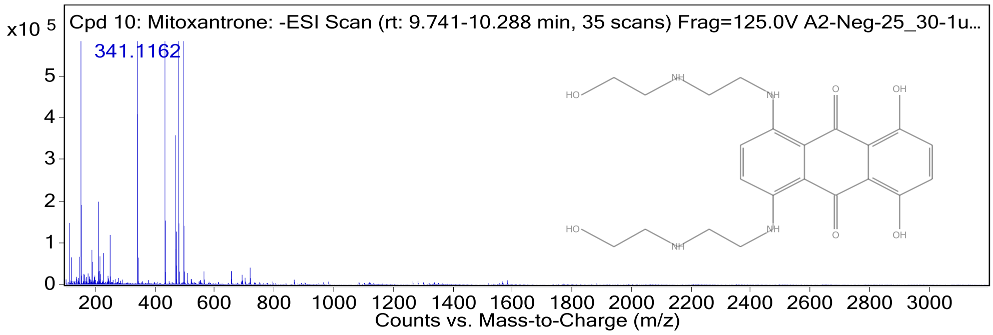

MS Zoomed Spectrum

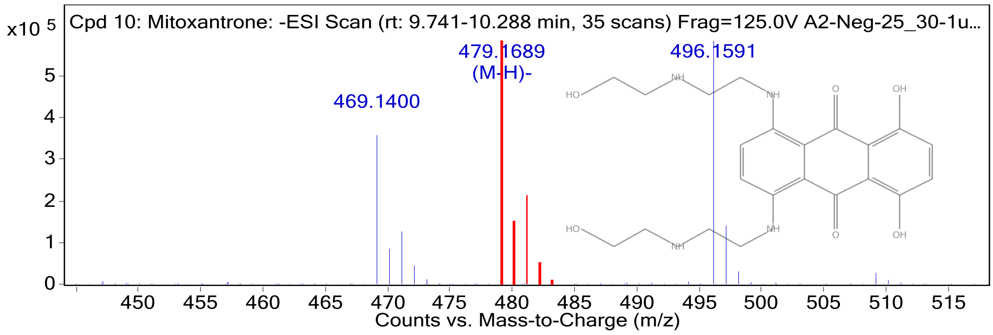

Compound Structure

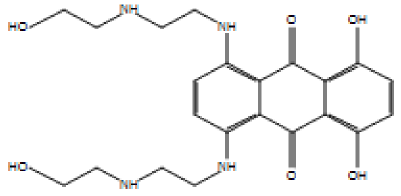

Compound Label	Name	*m/z*	RT	Algorithm	Mass
Cpd 11: Clozapine-N-Oxide	**Clozapine-N-Oxide**	341.1176	9.795	Find by Molecular Feature	342.1251

Compound Chromatograms

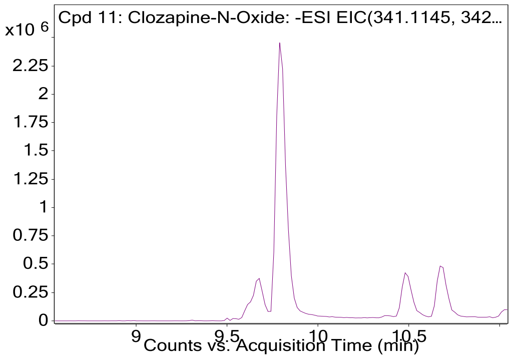

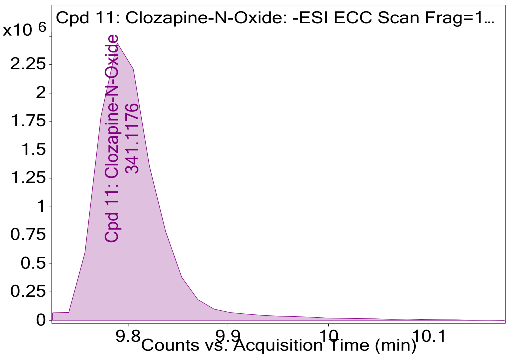

MFE MS Spectrum

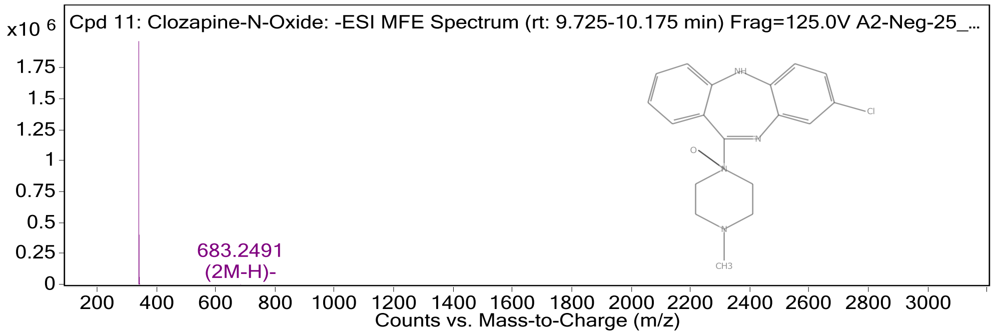

MFE MS Zoomed Spectrum

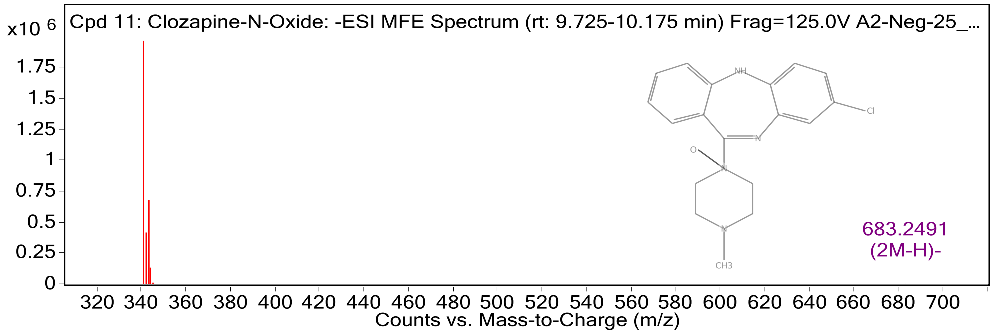

MS Spectrum Peak List

m/z	z	Abund	Formula	Ion
341.1176	-1	1964620.75	C18 H19 Cl N4 O	(M-H)-
342.1207	-1	401156.94	C18 H19 Cl N4 O	(M-H)-
343.1226	-1	59440.52	C18 H19 Cl N4 O	(M-H)-
344.1248	-1	6434.84	C18 H19 Cl N4 O	(M-H)-
345.1179	-1	622.97	C18 H19 Cl N4 O	(M-H)-
683.2491	-1	1188.33		(2M-H)-
684.2491	-1	777.94		(2M-H)-
685.2344	-1	707.71		(2M-H)-

MS Spectrum

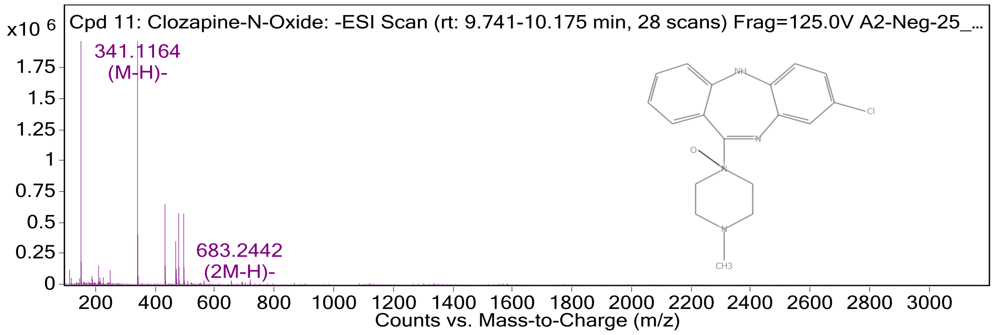

MS Zoomed Spectrum

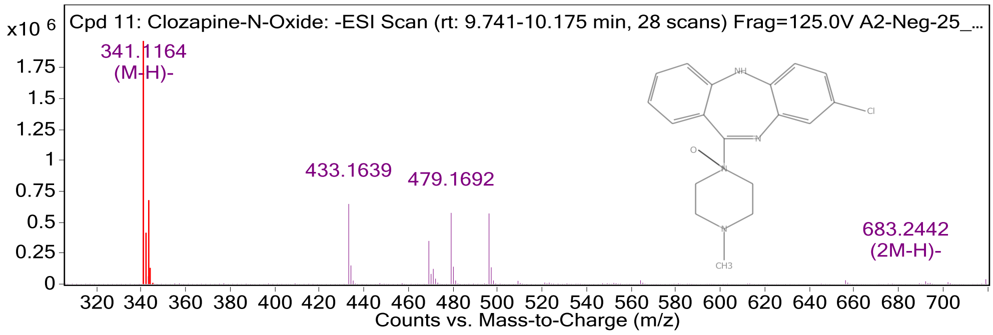

Compound Structure

Qualitative Compound Report

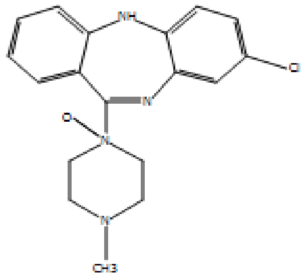

Compound Label	Name	*m/z*	RT	Algorithm	Mass
Cpd 12: Scandenin	**Scandenin**	433.1658	9.796	Find by Molecular Feature	434.1729

Compound Chromatograms

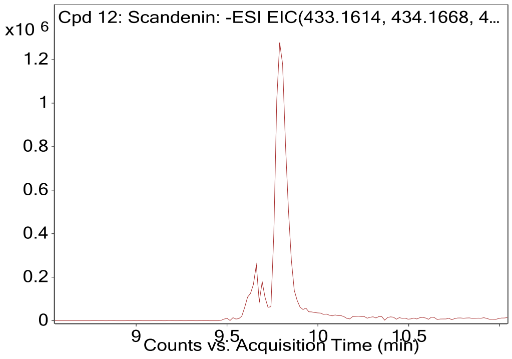

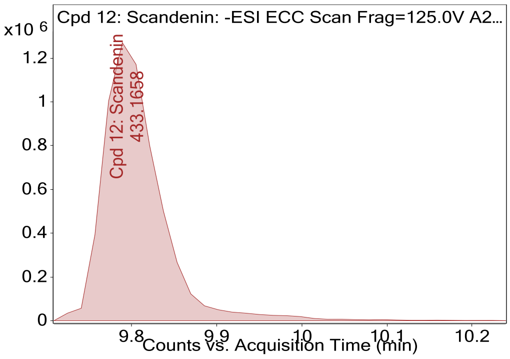

MFE MS Spectrum

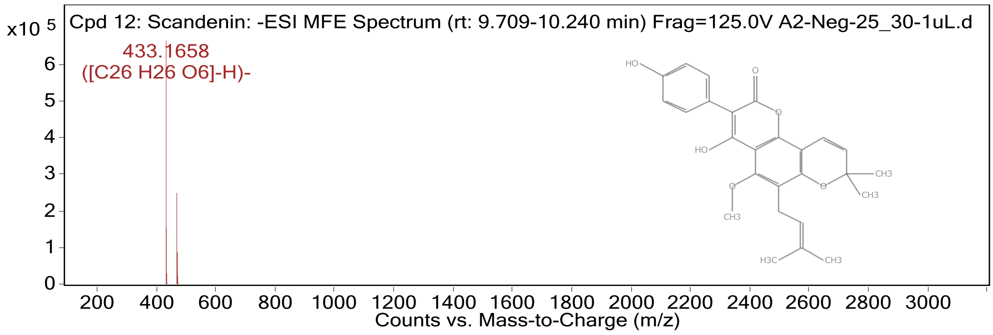

MFE MS Zoomed Spectrum

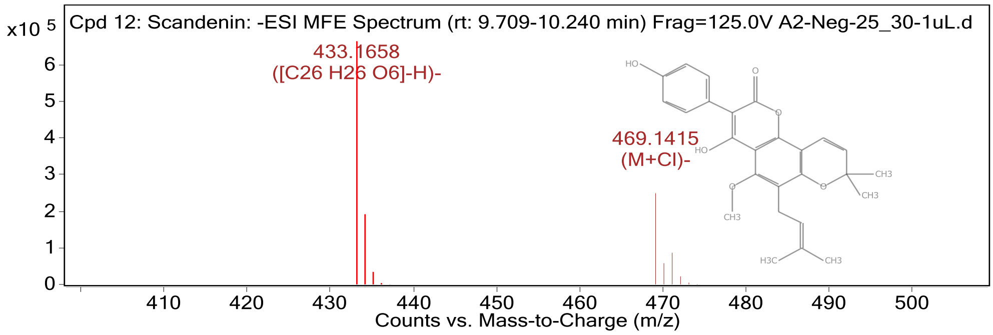

MS Spectrum Peak List

m/z	z	Abund	Formula	Ion
433.1658	-1	663987.19	C26 H26 O6	(M-H)-
434.1684	-1	153409.22	C26 H26 O6	(M-H)-
435.1705	-1	28480.6	C26 H26 O6	(M-H)-
436.1718	-1	3954.18	C26 H26 O6	(M-H)-

469.1415	-1	248983.44		(M+Cl)-
470.1441	-1	58002.31		(M+Cl)-
471.1391	-1	86436.54		(M+Cl)-
472.1441	-1	21853.23		(M+Cl)-
473.1478	-1	4920.6		(M+Cl)-
474.1513	-1	973.42		(M+Cl)-

MS Spectrum

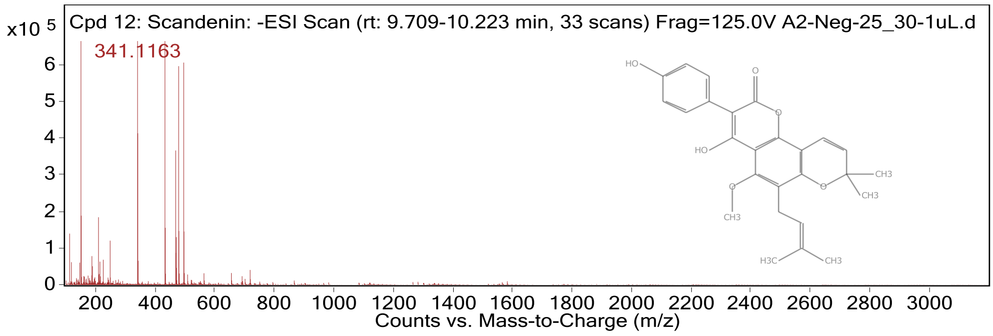

MS Zoomed Spectrum

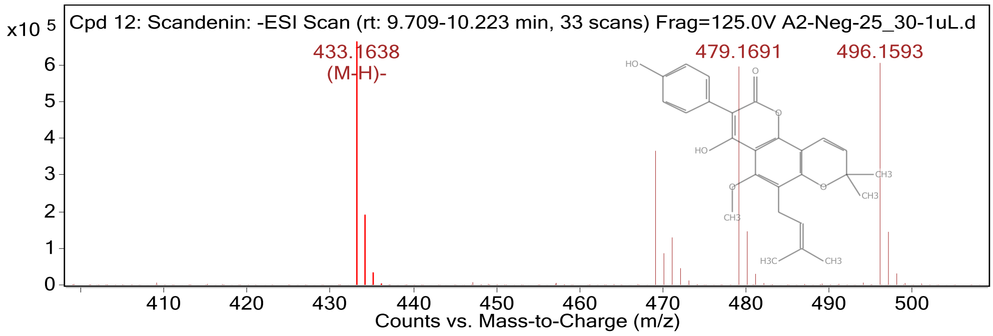

Compound Structure

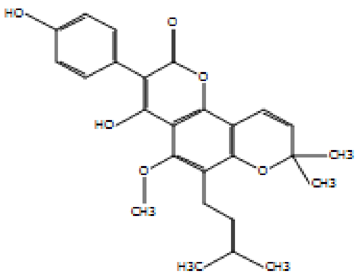

**Compound Label**	**Name**	***m/z***	**RT**	**Algorithm**	**Mass**
Cpd 13: Idarubicin	**Idarubicin**	496.1613	9.796	Find by Molecular Feature	497.1684

Compound Chromatograms

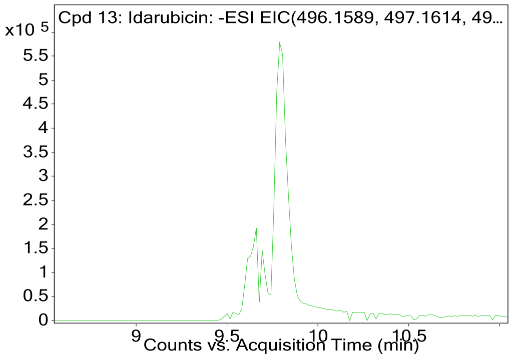

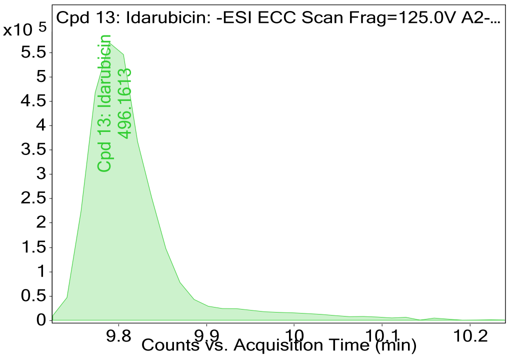

MFE MS Spectrum

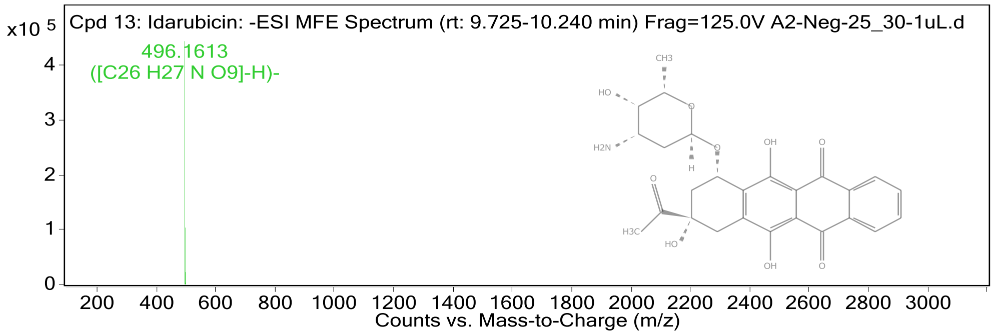

MFE MS Zoomed Spectrum

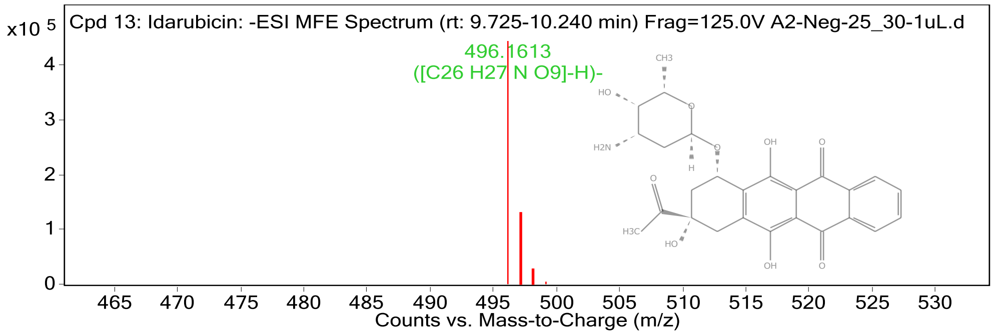

MS Spectrum Peak List

m/z	z	Abund	Formula	Ion
496.1613	-1	444615.06	C26 H27 N O9	(M-H)-
497.1639	-1	103756.49	C26 H27 N O9	(M-H)-
498.1652	-1	22402.65	C26 H27 N O9	(M-H)-
499.1672	-1	3245.47	C26 H27 N O9	(M-H)-

MS Spectrum

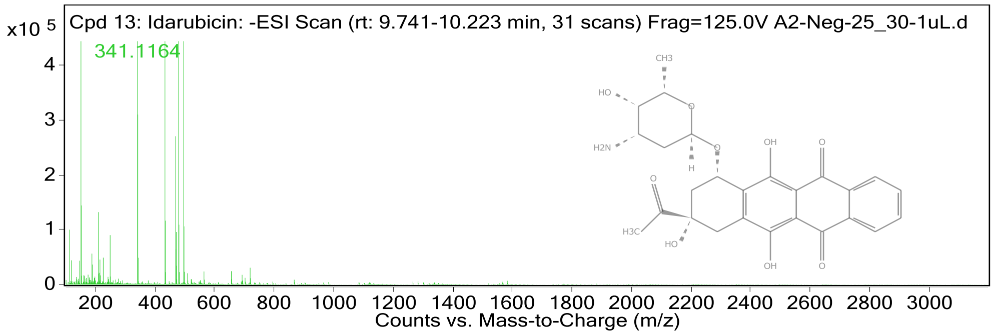

MS Zoomed Spectrum

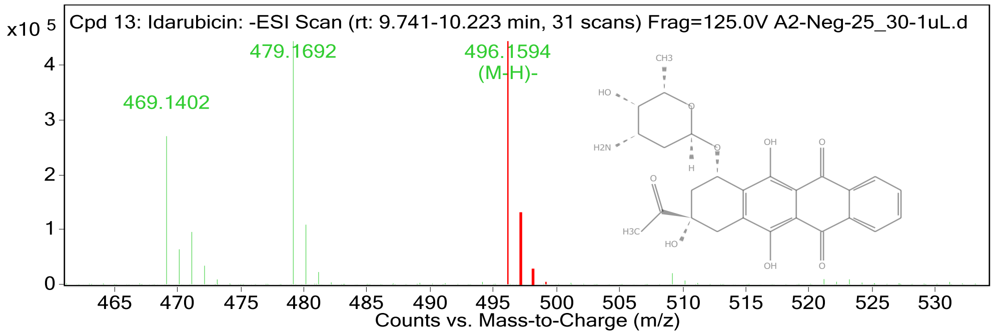

Compound Structure

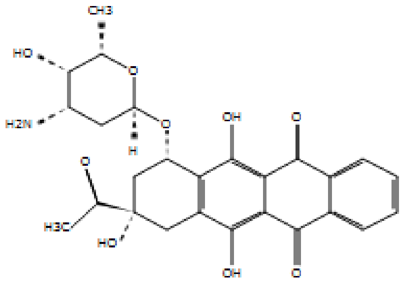

Compound Label	*m/z*	RT	Algorithm	Mass
Cpd 14: 9.889	151.041	9.889	Find by Molecular Feature	152.0483

Compound Chromatograms

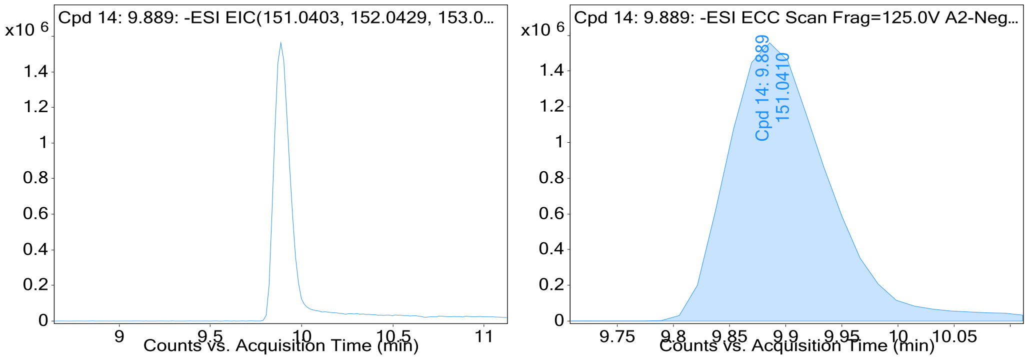

MFE MS Spectrum

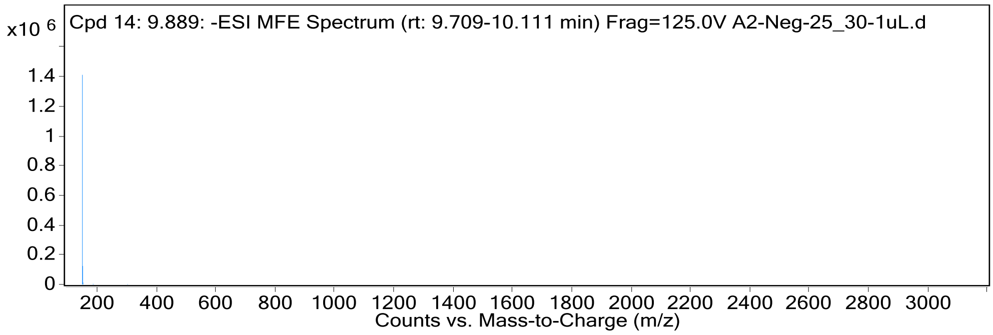

MFE MS Zoomed Spectrum

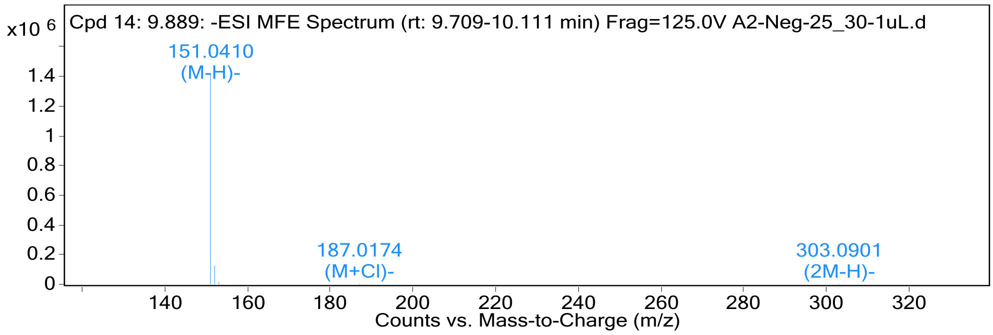

MS Spectrum Peak List

m/z	z	Abund	Ion
151.041	-1	1411415.5	(M-H)-
152.0442	-1	123953.3	(M-H)-
153.0459	-1	13907.71	(M-H)-
154.0414	-1	1262.22	(M-H)-
187.0174	-1	3554.78	(M+Cl)-
188.0191	-1	532.41	(M+Cl)-
189.0136	-1	1351	(M+Cl)-
303.0901	-1	2477.01	(2M-H)-
304.0891	-1	541.92	(2M-H)-

MS Spectrum

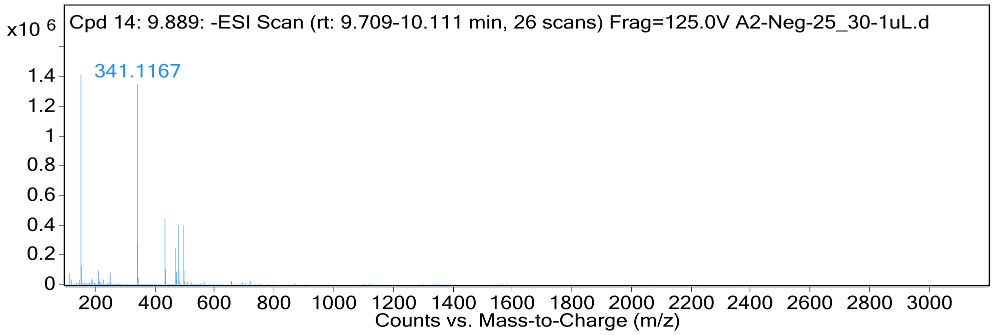

MS Zoomed Spectrum

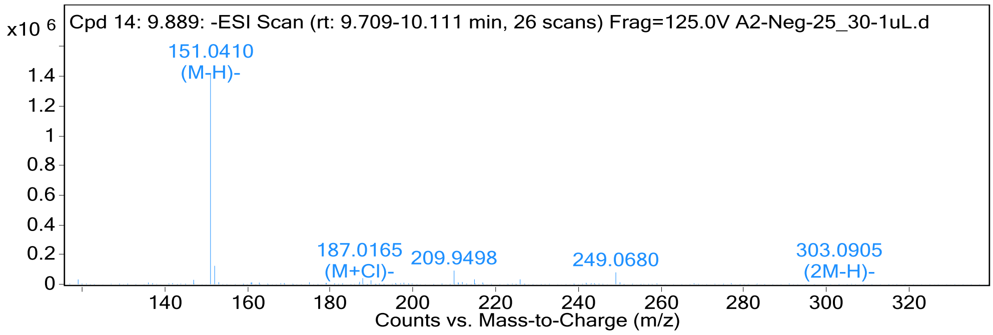

**Compound Label**	**Name**	***m/z***	**RT**	**Algorithm**	**Mass**
Cpd 15: Dictyoquinazol C	**Dictyoquinazol C**	341.1151	10.488	Find by Molecular Feature	342.1223

Compound Chromatograms

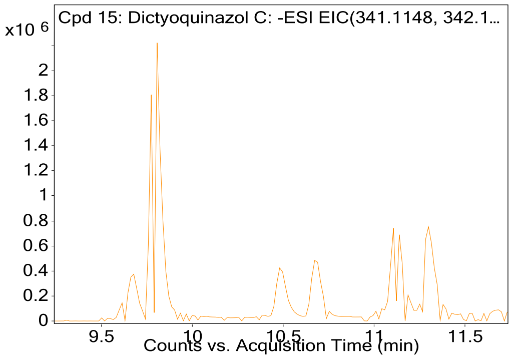

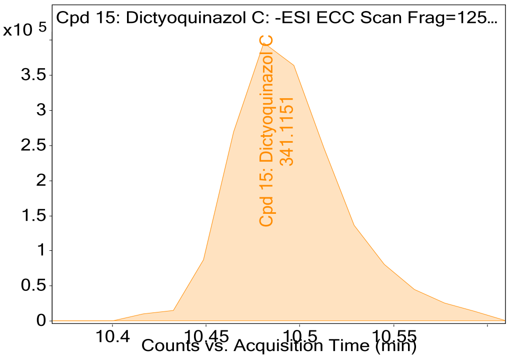

MFE MS Spectrum

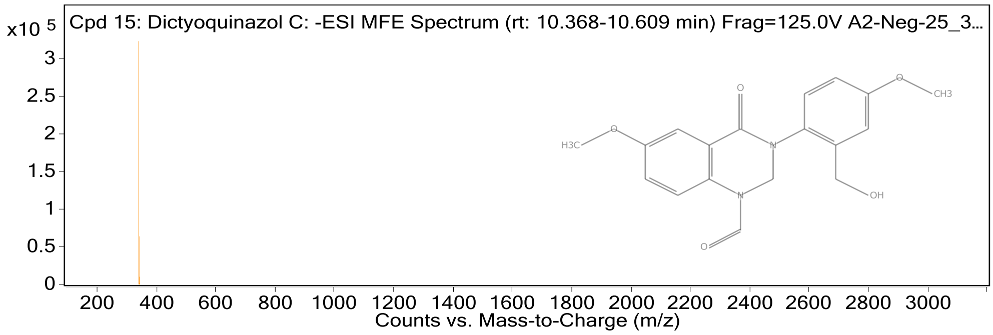

MFE MS Zoomed Spectrum

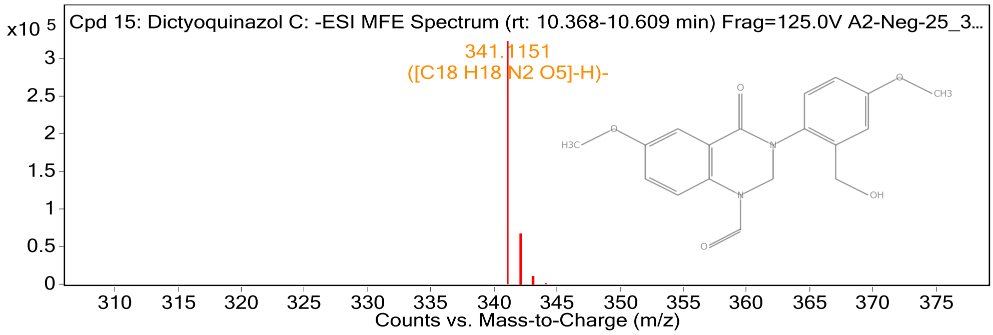

MS Spectrum Peak List

m/z	z	Abund	Formula	Ion
341.1151	-1	323473.66	C18 H18 N2 O5	(M-H)-
342.1178	-1	63927.06	C18 H18 N2 O5	(M-H)-
343.1209	-1	10110.96	C18 H18 N2 O5	(M-H)-
344.1226	-1	1134.17	C18 H18 N2 O5	(M-H)-

MS Spectrum

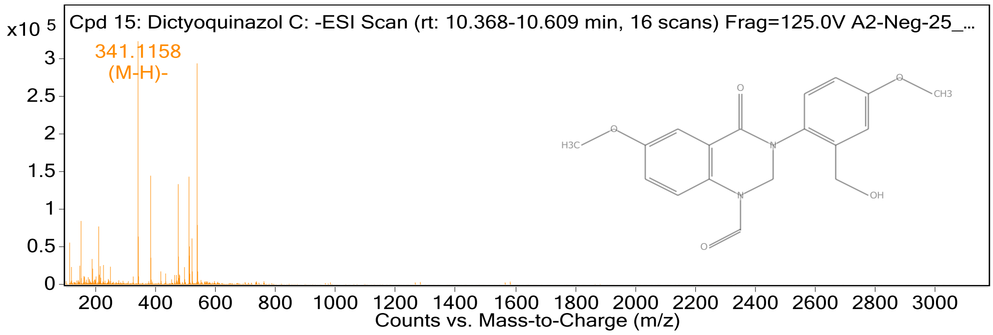

MS Zoomed Spectrum

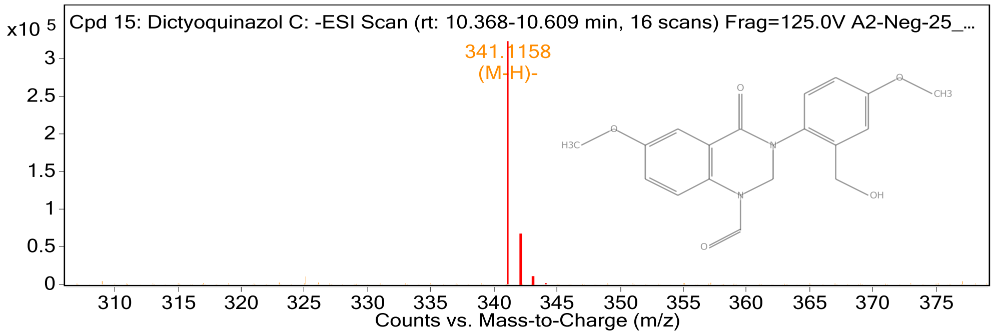

Compound Structure

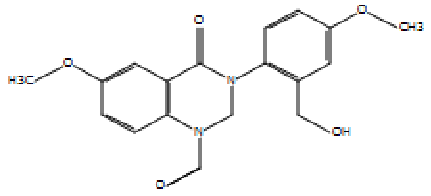

**Compound Label**	**Name**	***m/z***	**RT**	**Algorithm**	**Mass**
Cpd 16: Dictyoquinazol C	**Dictyoquinazol C**	341.1157	10.681	Find by Molecular Feature	342.1229

Compound Chromatograms

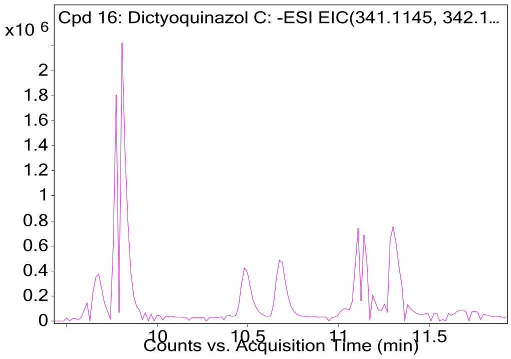

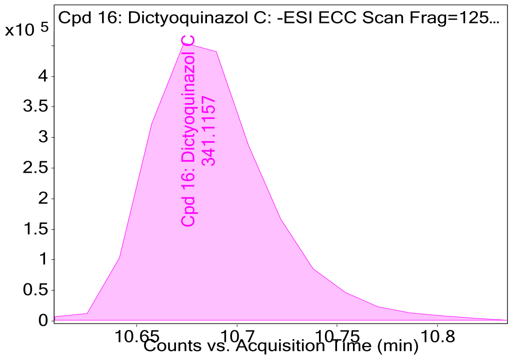

MFE MS Spectrum

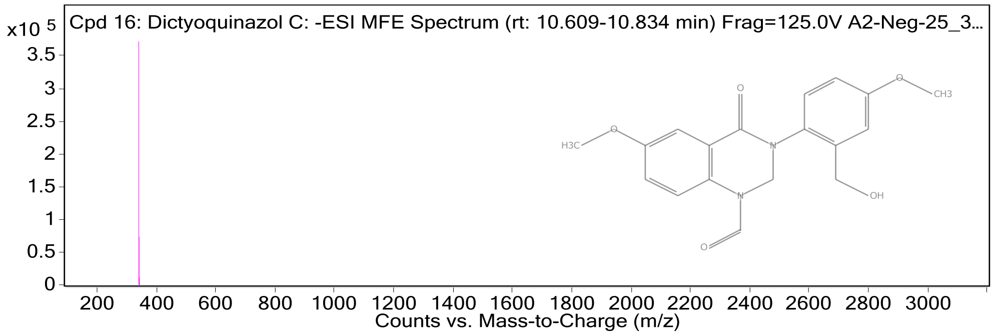

MFE MS Zoomed Spectrum

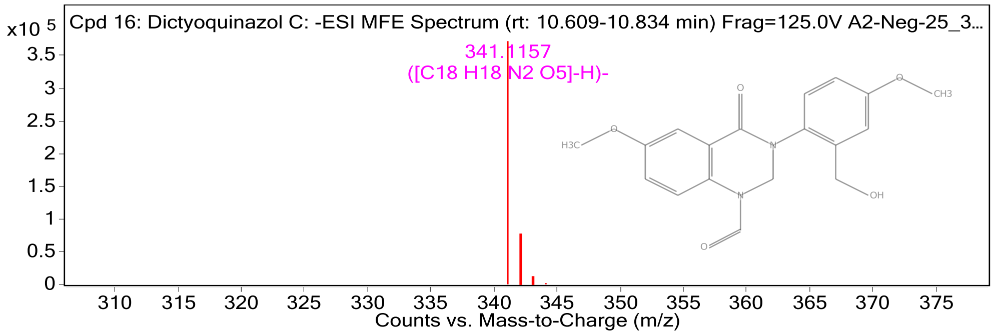

MS Spectrum Peak List

m/z	z	Abund	Formula	Ion
341.1157	-1	371951.34	C18 H18 N2 O5	(M-H)-
342.1183	-1	73350.96	C18 H18 N2 O5	(M-H)-
343.1198	-1	11169.54	C18 H18 N2 O5	(M-H)-
344.1228	-1	1317.07	C18 H18 N2 O5	(M-H)-

MS Spectrum

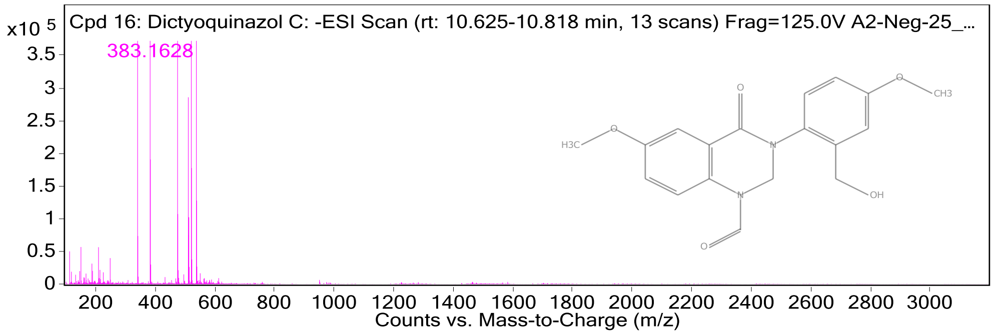

MS Zoomed Spectrum

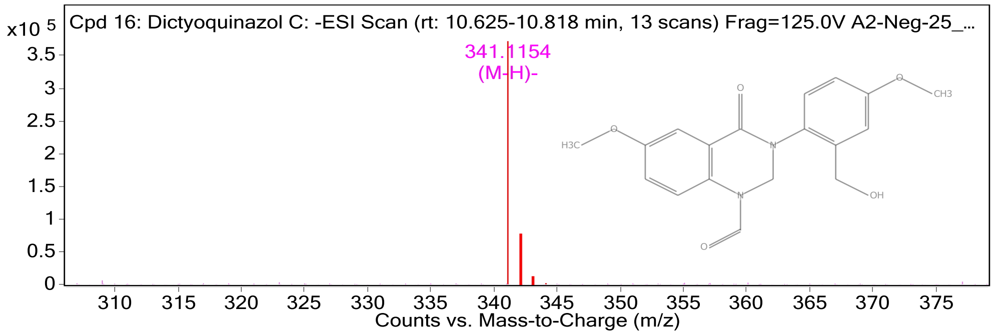

Compound Structure

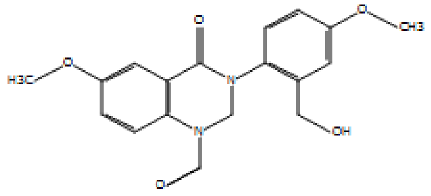

Compound Label	Name	*m/z*	RT	Algorithm	Mass
Cpd 17: Chlorovulone III	**Chlorovulone III**	383.163	10.681	Find by Molecular Feature	348.1937

Compound Chromatograms

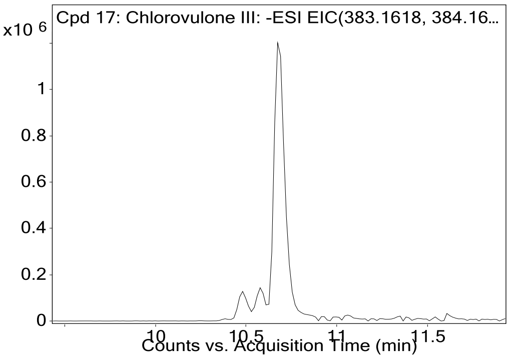

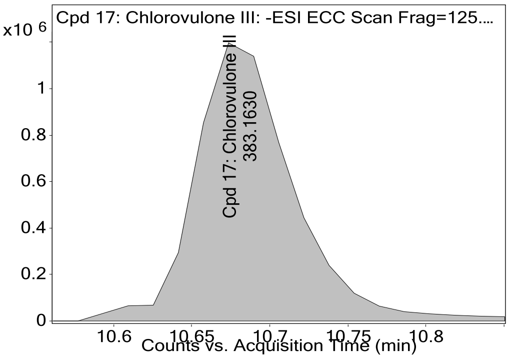

MFE MS Spectrum

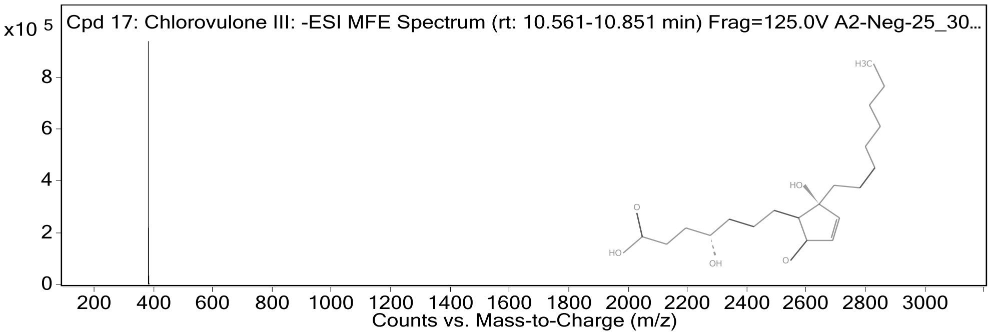

MFE MS Zoomed Spectrum

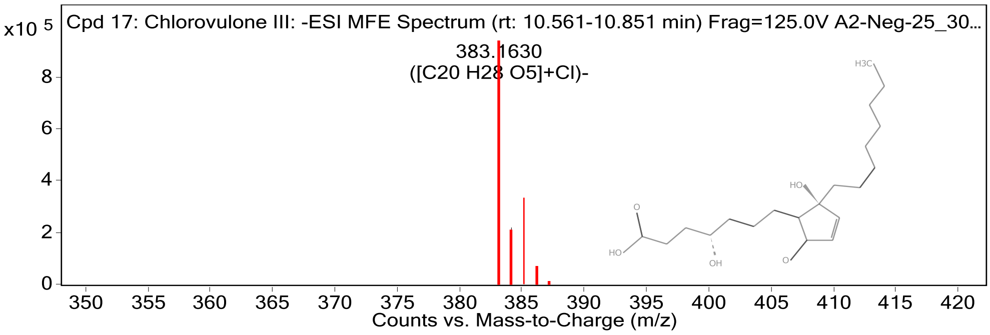

MS Spectrum Peak List

m/z	z	Abund	Formula	Ion
383.163	-1	937917.31	C20 H28 O5	(M+Cl)-
384.1657	-1	218670.64	C20 H28 O5	(M+Cl)-
385.1678	-1	33240.29	C20 H28 O5	(M+Cl)-
386.1712	-1	3985.63	C20 H28 O5	(M+Cl)-
387.1674	-1	158.58	C20 H28 O5	(M+Cl)-

MS Spectrum

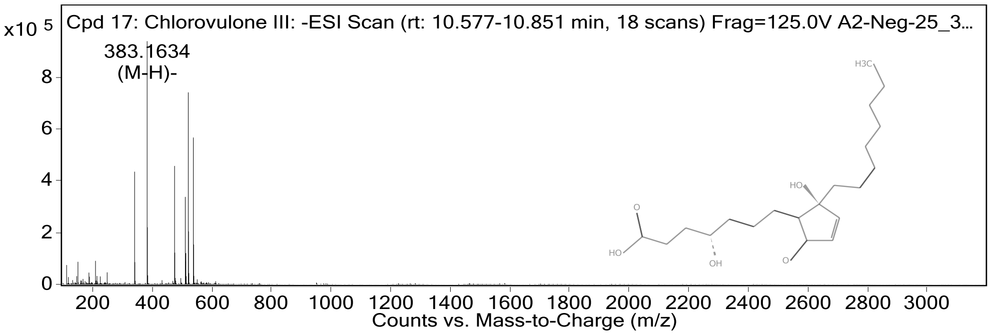

MS Zoomed Spectrum

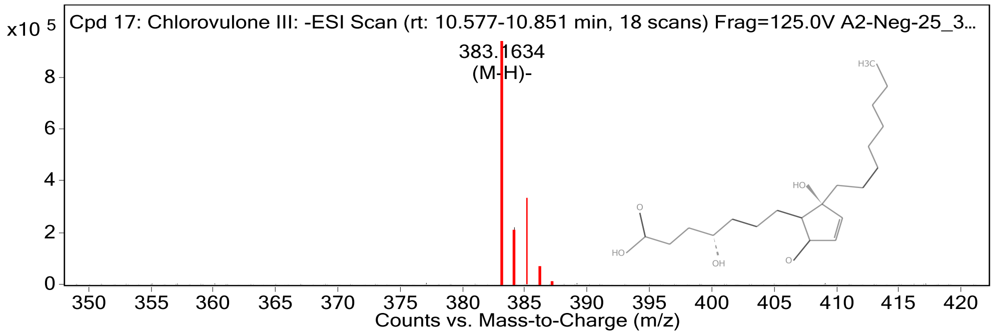

Compound Structure

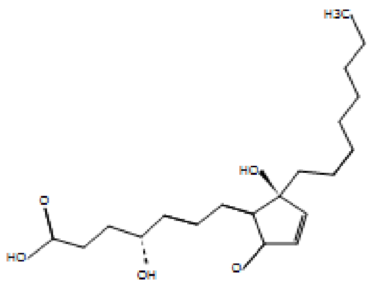

Compound Label	Name	*m/z*	RT	Algorithm	Mass
Cpd 18: Carpelastofuran	**Carpelastofuran**	521.2159	10.682	Find by Molecular Feature	522.2229

Compound Chromatograms

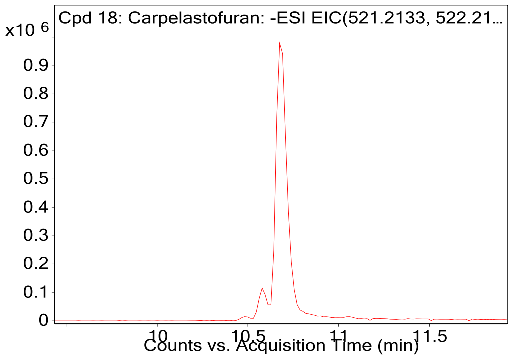

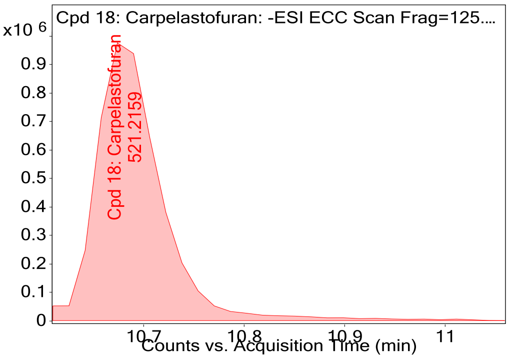

MFE MS Spectrum

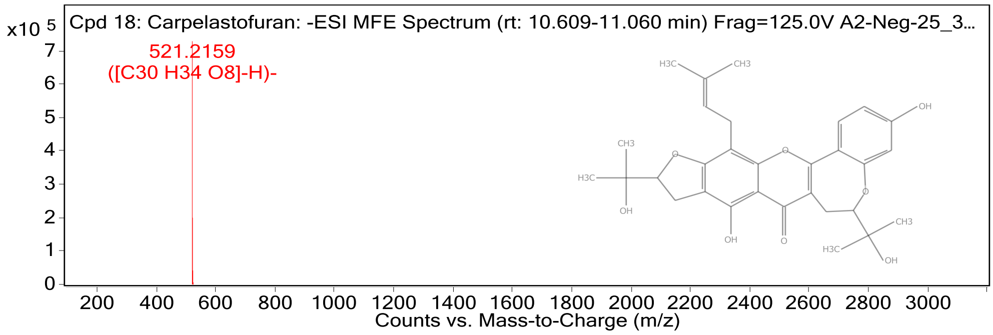

MFE MS Zoomed Spectrum

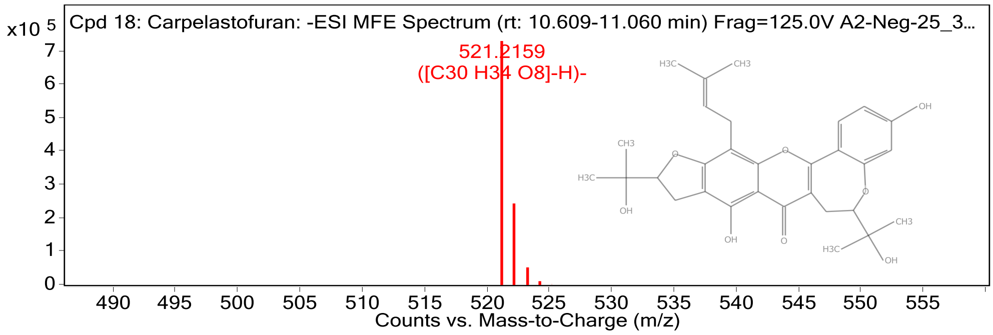

MS Spectrum Peak List

m/z	z	Abund	Formula	Ion
521.2159	-1	728566	C30 H34 O8	(M-H)-
522.2186	-1	199846.09	C30 H34 O8	(M-H)-
523.2201	-1	41696.03	C30 H34 O8	(M-H)-
524.2238	-1	6379.81	C30 H34 O8	(M-H)-
525.2133	-1	1094.72	C30 H34 O8	(M-H)-

MS Spectrum

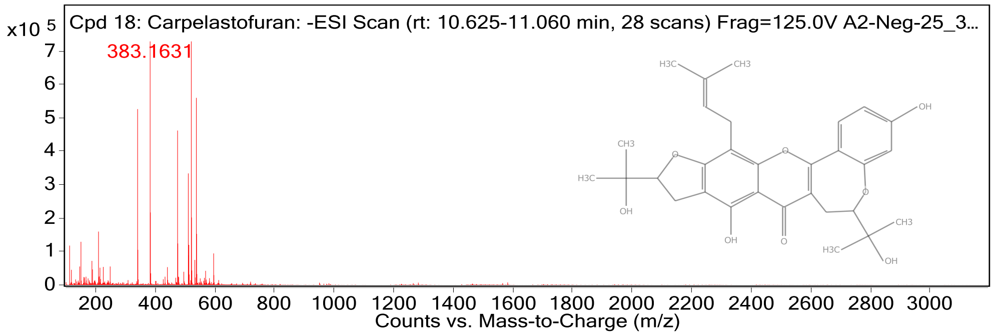

MS Zoomed Spectrum

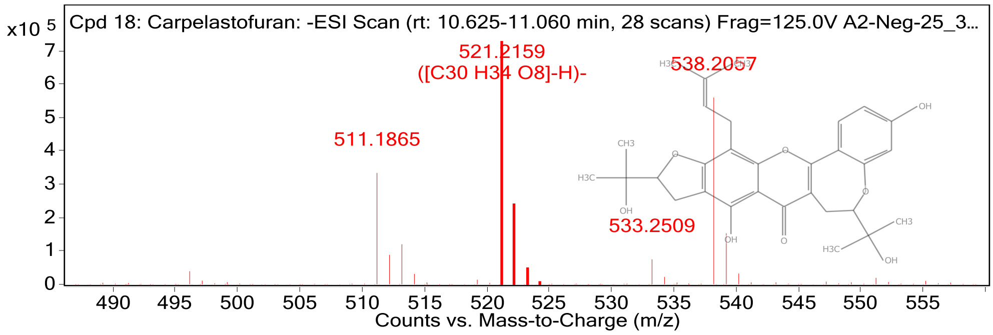

Compound Structure

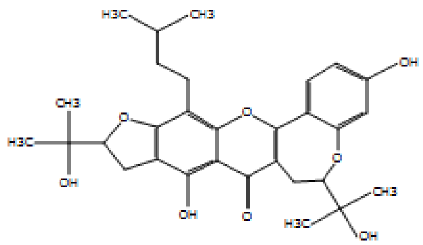

Compound Label	*m/z*	RT	Algorithm	Mass
Cpd 19: 10.682	475.2103	10.682	Find by Molecular Feature	476.2174

Qualitative Compound Report

Compound Chromatograms

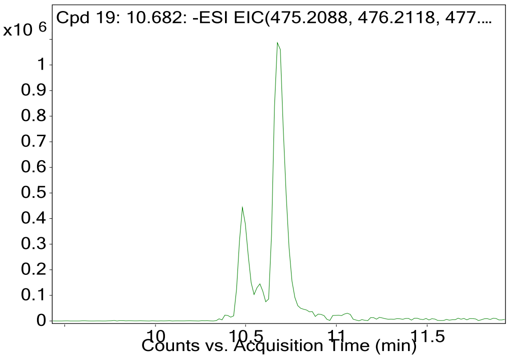

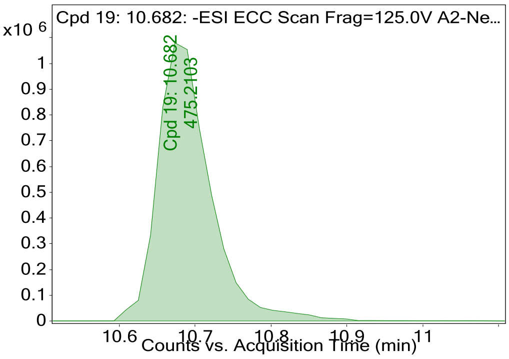

MFE MS Spectrum

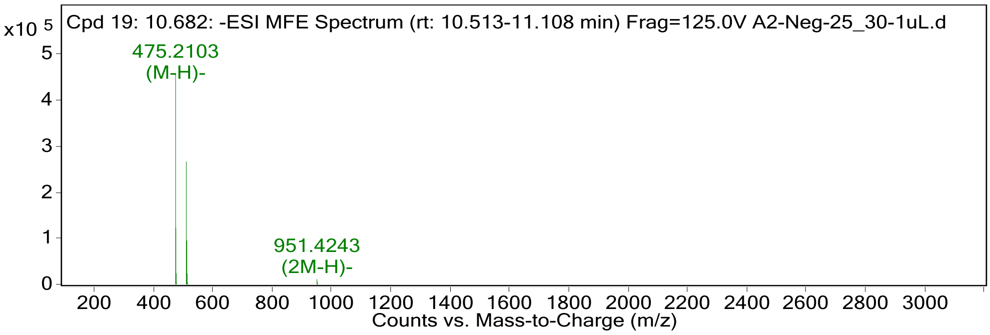

MFE MS Zoomed Spectrum

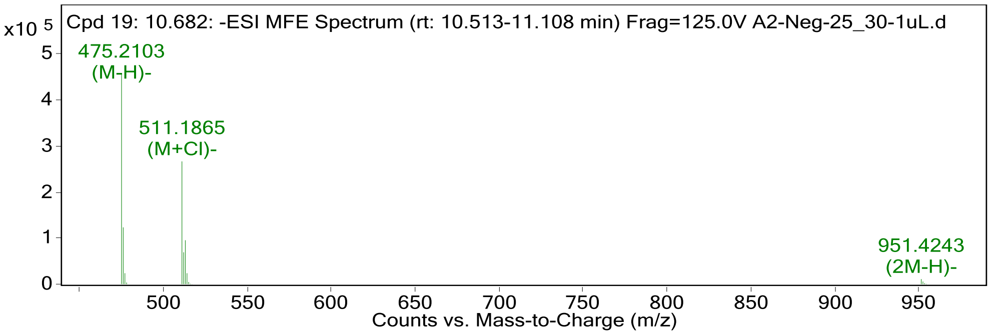

MS Spectrum Peak List

m/z	z	Abund	Ion
475.2103	-1	455488.84	(M-H)-
476.2128	-1	123441.19	(M-H)-
477.2151	-1	23822.25	(M-H)-
511.1865	-1	266830.97	(M+Cl)-
512.1894	-1	69271.08	(M+Cl)-
513.1842	-1	95497.98	(M+Cl)-
514.1864	-1	23813.01	(M+Cl)-
515.1916	-1	4700.44	(M+Cl)-
951.4243	-1	10951.1	(2M-H)-
952.4266	-1	5858.47	(2M-H)-

MS Spectrum

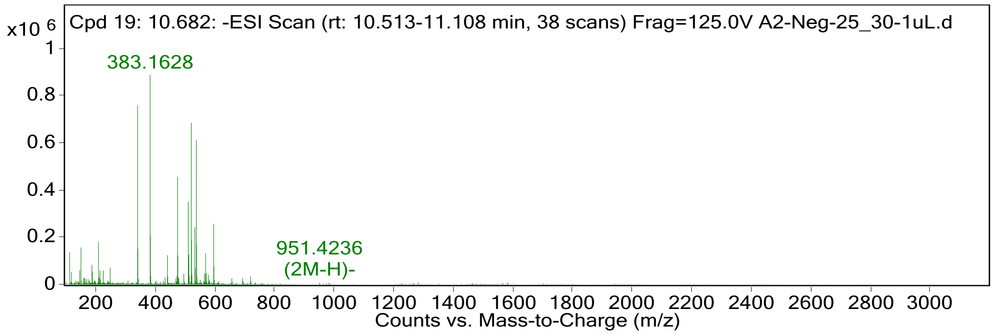

MS Zoomed Spectrum

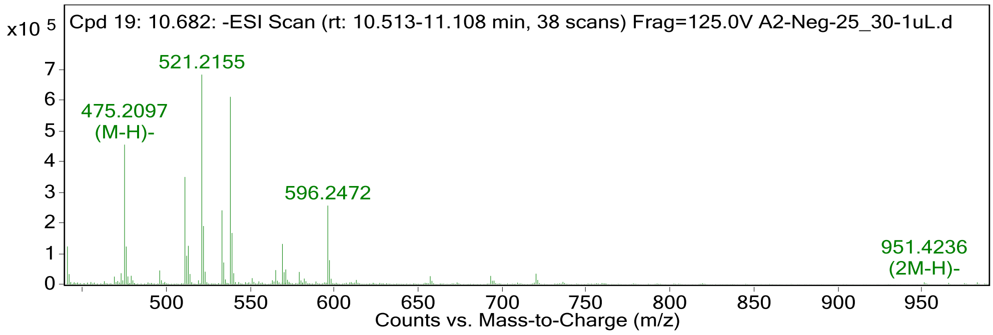

**Compound Label**	***m/z***	**RT**	**Algorithm**	**Mass**
Cpd 20: 10.684	538.2058	10.684	Find by Molecular Feature	539.2131

Compound Chromatograms

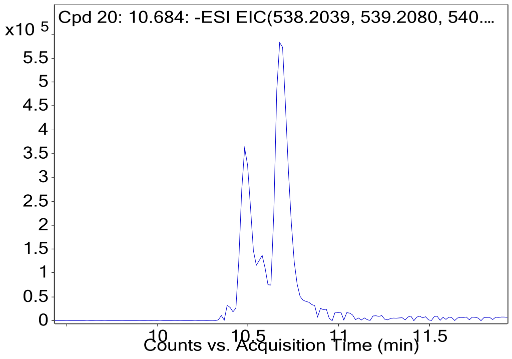

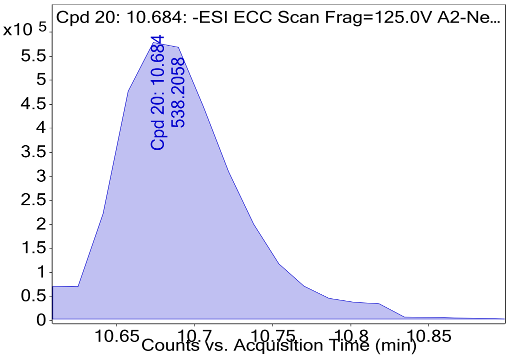

MFE MS Spectrum

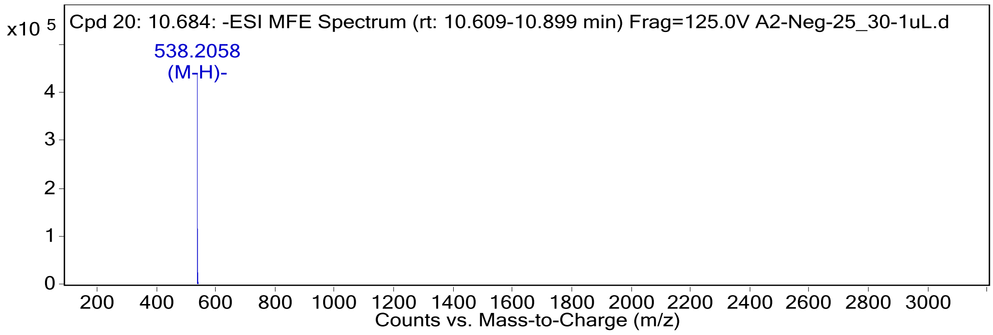

MFE MS Zoomed Spectrum

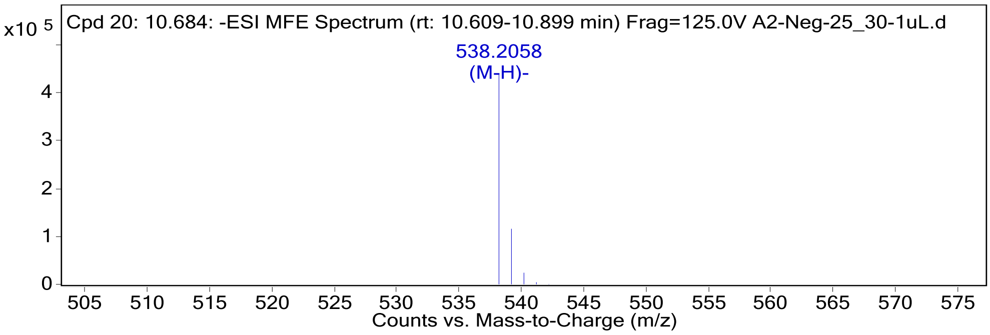

MS Spectrum Peak List

m/z	z	Abund	Ion
538.2058	-1	437324.25	(M-H)-
539.2083	-1	115880.56	(M-H)-
540.21	-1	24223.25	(M-H)-
541.2112	-1	4358.46	(M-H)-
542.2118	-1	722.46	(M-H)-

MS Spectrum

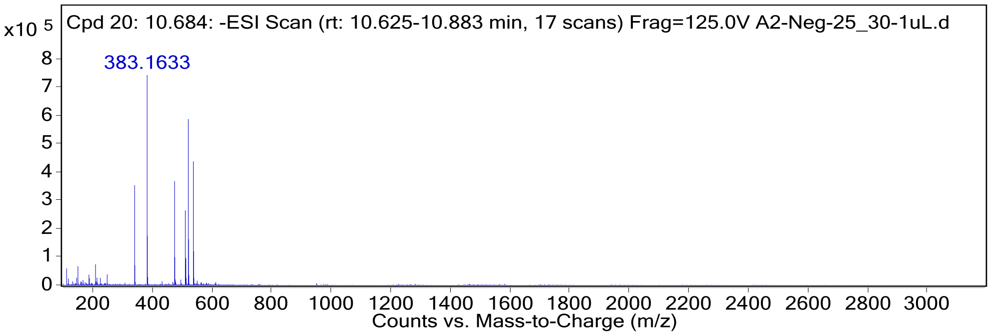

MS Zoomed Spectrum

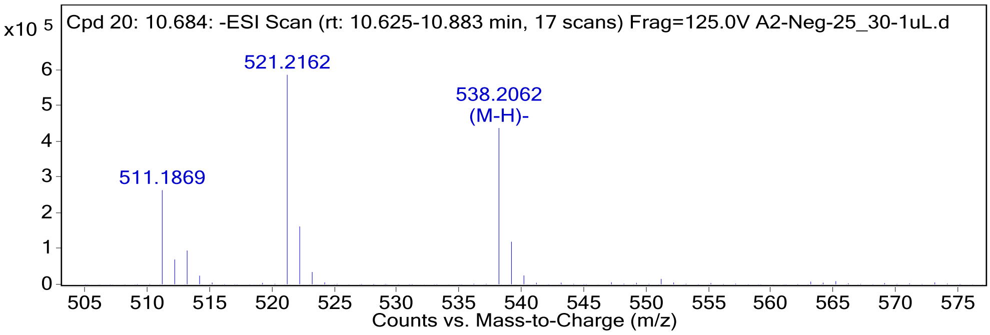

Compound Label	Name	*m/z*	RT	Algorithm	Mass
Cpd 21: Antimycin A (A1 Shown)	**Antimycin A (A1 Shown)**	533.2505	11.115	Find by Molecular Feature	534.2576

Compound Chromatograms

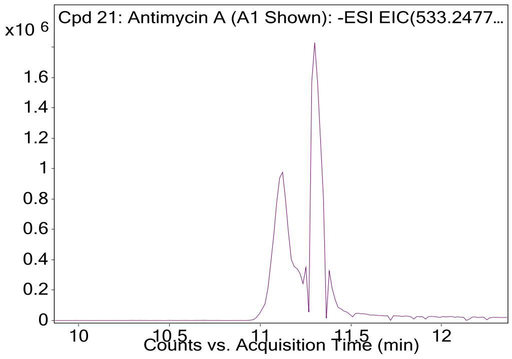

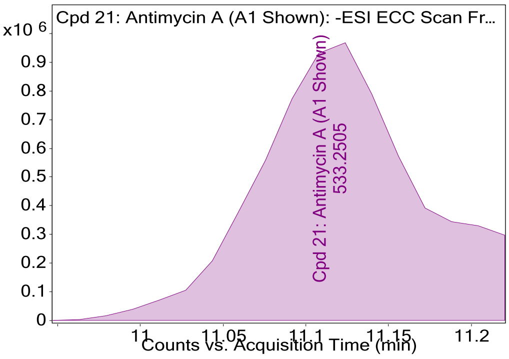

MFE MS Spectrum

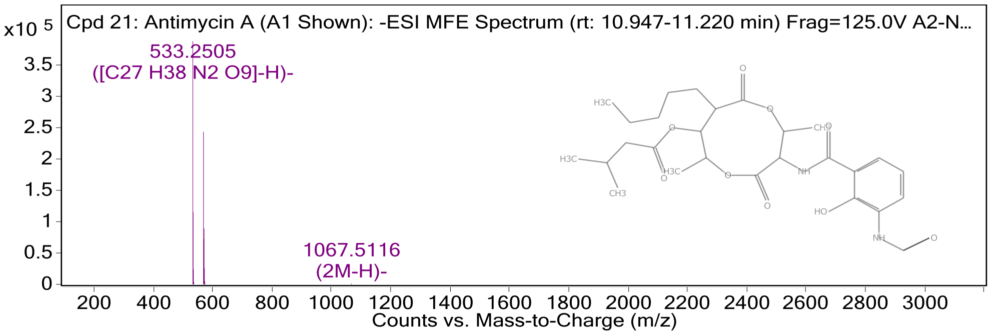

MFE MS Zoomed Spectrum

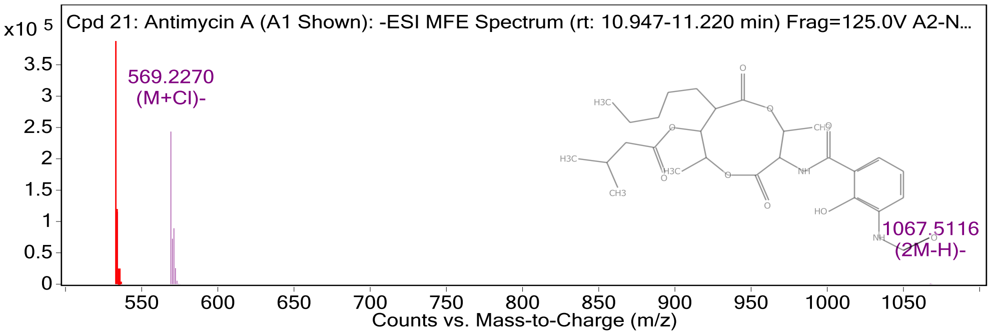

MS Spectrum Peak List

m/z	z	Abund	Formula	Ion
533.2505	-1	388180.56	C27 H38 N2 O9	(M-H)-
534.2533	-1	115421.8	C27 H38 N2 O9	(M-H)-
535.2551	-1	23715.19	C27 H38 N2 O9	(M-H)-
536.2564	-1	3884.67	C27 H38 N2 O9	(M-H)-
569.227	-1	243629.23		(M+Cl)-
570.2296	-1	72648.6		(M+Cl)-
571.2246	-1	89268.82		(M+Cl)-
572.2271	-1	25614.74		(M+Cl)-
573.2302	-1	5473.4		(M+Cl)-
1067.5116	-1	1109.78		(2M-H)-

MS Spectrum

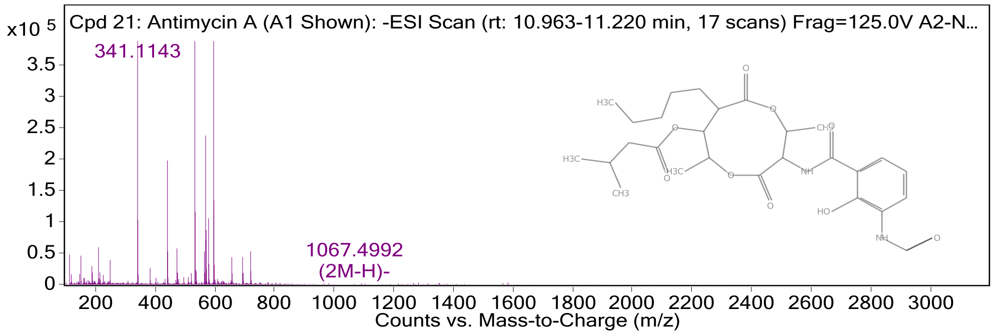

MS Zoomed Spectrum

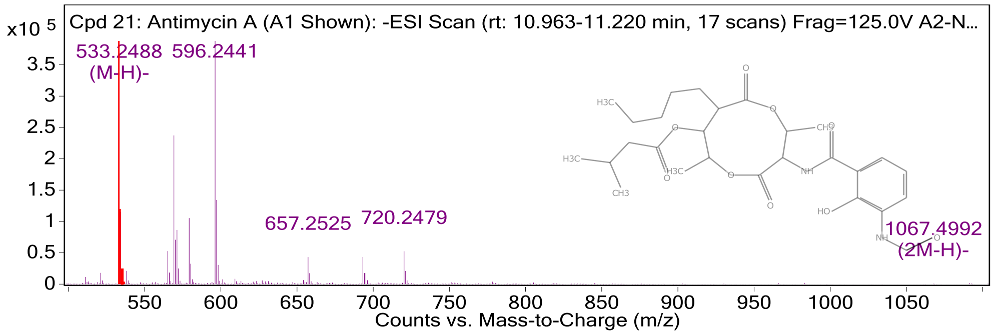

Compound Structure

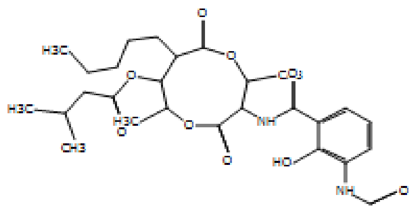

Compound Label	*m/z*	RT	Algorithm	Mass
Cpd 22: 11.119	596.2462	11.119	Find by Molecular Feature	597.2534

Compound Chromatograms

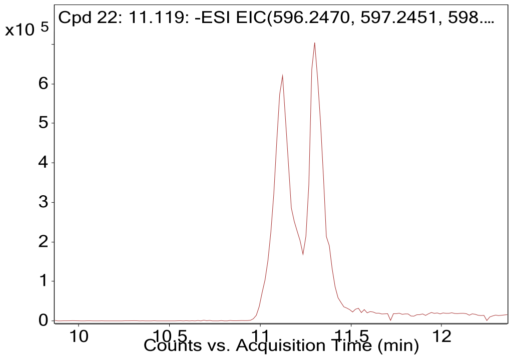

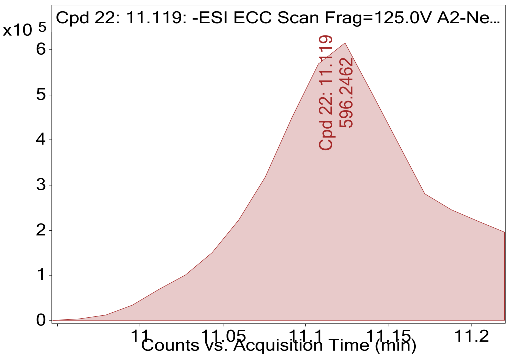

MFE MS Spectrum

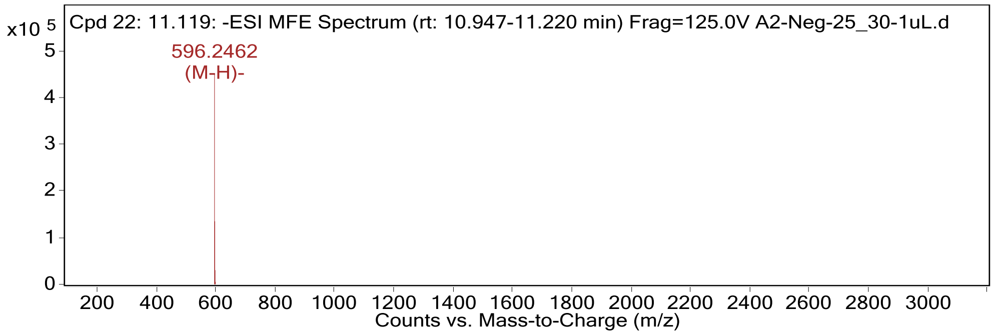

MFE MS Zoomed Spectrum

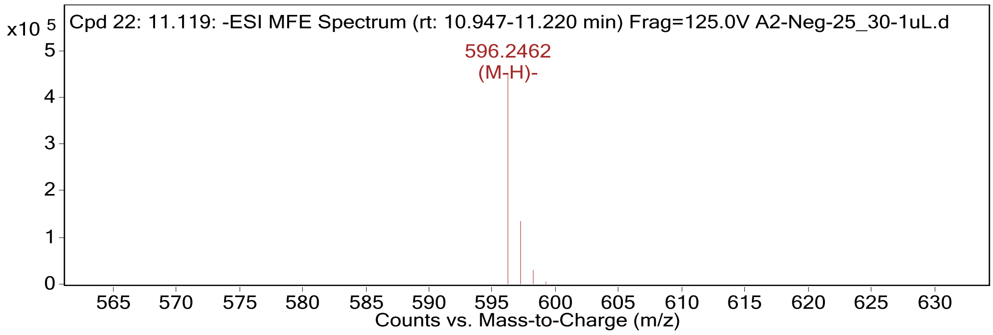

MS Spectrum Peak List

m/z	z	Abund	Ion
596.2462	-1	449068.19	(M-H)-
597.2482	-1	134897.12	(M-H)-
598.2503	-1	30284.01	(M-H)-
599.2523	-1	5232.23	(M-H)-

MS Spectrum

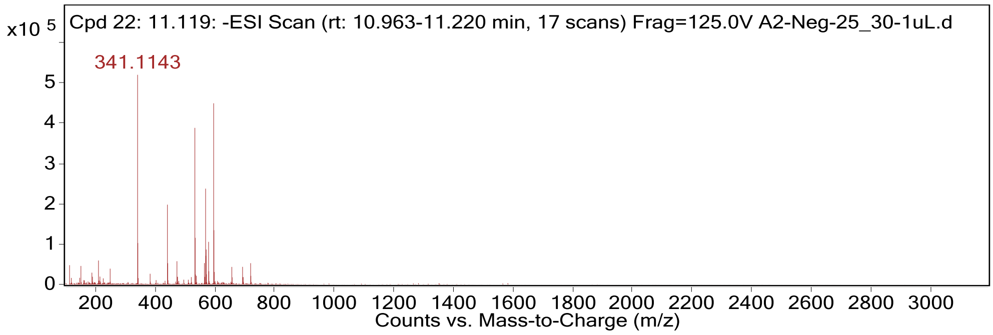

MS Zoomed Spectrum

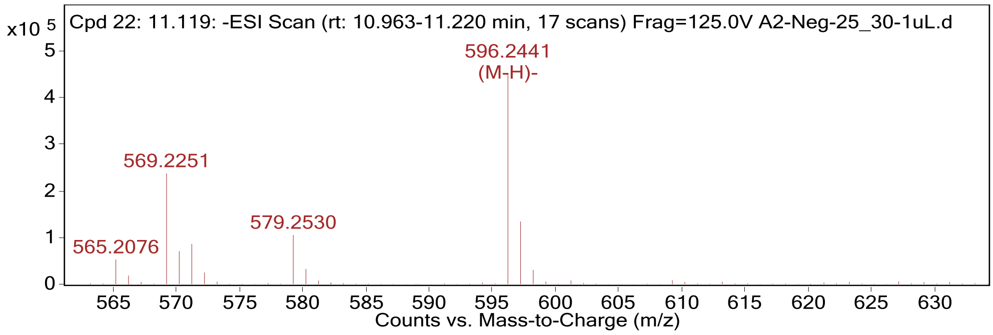

Compound Label	Name	*m/z*	RT	Algorithm	Mass
Cpd 23: Dictyoquinazol C	**Dictyoquinazol C**	341.1159	11.124	Find by Molecular Feature	342.1231

Compound Chromatograms

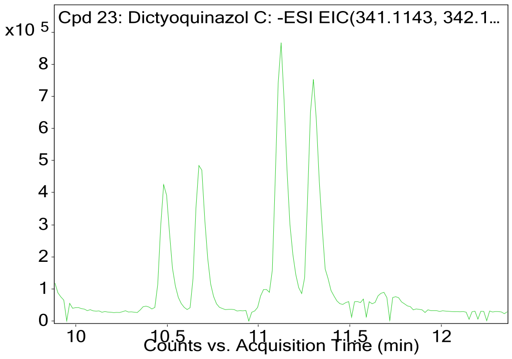

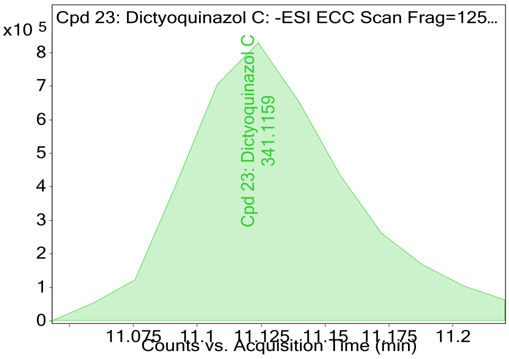

MFE MS Spectrum

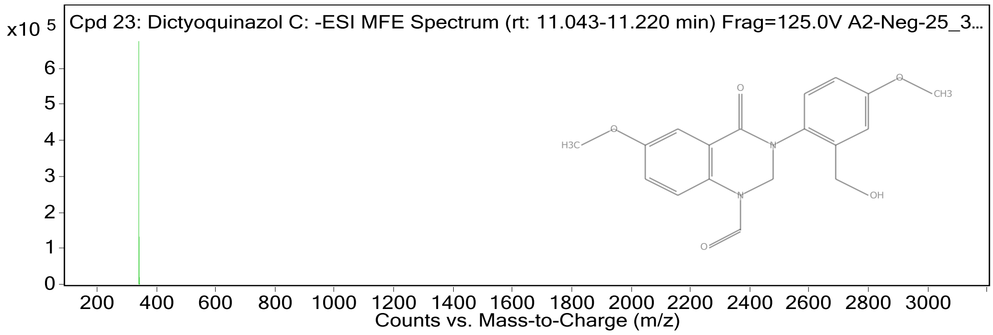

MFE MS Zoomed Spectrum

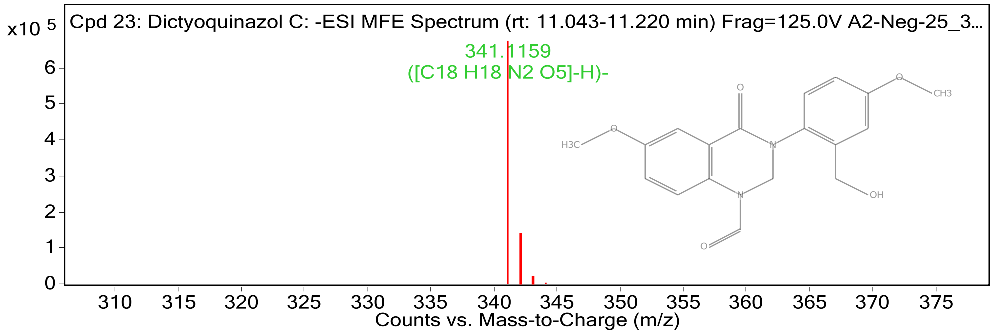

MS Spectrum Peak List

m/z	z	Abund	Formula	Ion
341.1159	-1	675526.81	C18 H18 N2 O5	(M-H)-
342.1185	-1	132269.06	C18 H18 N2 O5	(M-H)-
343.1205	-1	19775.47	C18 H18 N2 O5	(M-H)-
344.1236	-1	2004.28	C18 H18 N2 O5	(M-H)-

MS Spectrum

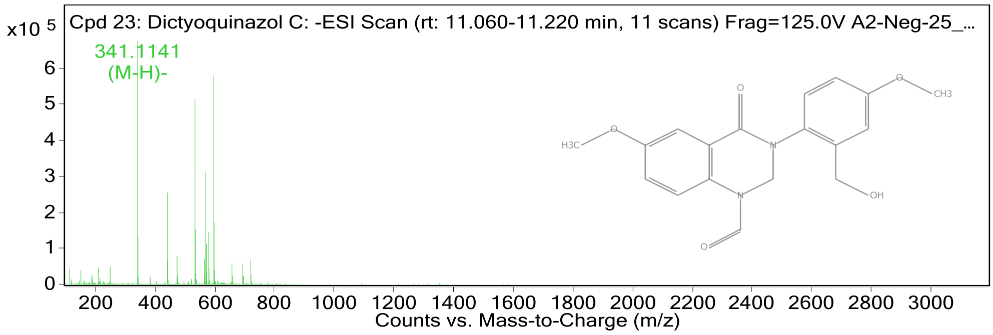

MS Zoomed Spectrum

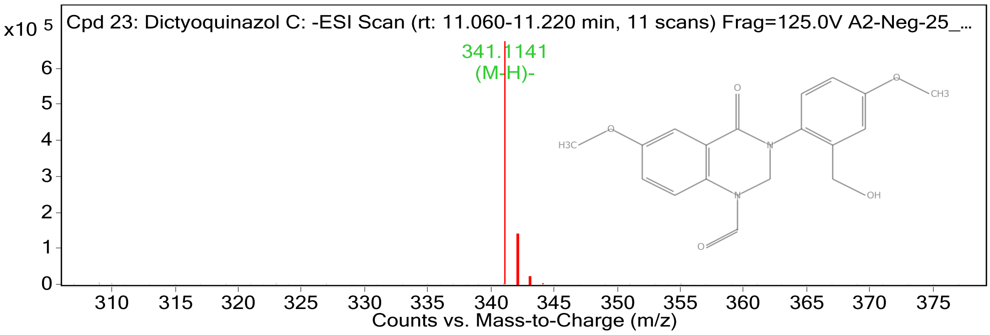
x10 5
Cpd 23: Dictyoquinazol C: -ESI Scan (rt: 11.060-11.220 min, 11 scans) Frag=125.0V A2-Neg-25_...
341.1141
(M-H)-
6
5
4
3
2
1
0
310
315
320
325
330
335
340
345
350
355
360
365
370
375
Counts vs. Mass-to-Charge (m/z)
O
N
H3C
O
CH3
OH

Qualitative Compound Report

Compound Structure

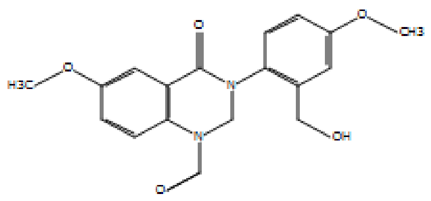

**Compound Label**	**Name**	***m/z***	**RT**	**Algorithm**	**Mass**
Cpd 24: Ligulatin B	**Ligulatin B**	341.1165	11.302	Find by Molecular Feature	306.1472

Compound Chromatograms

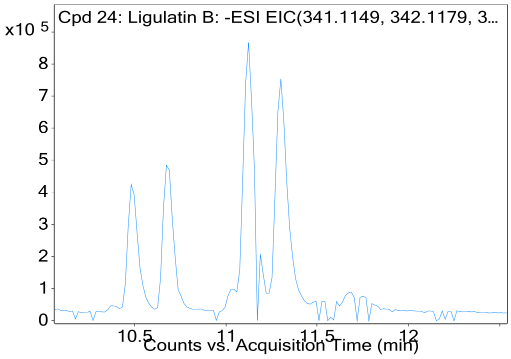

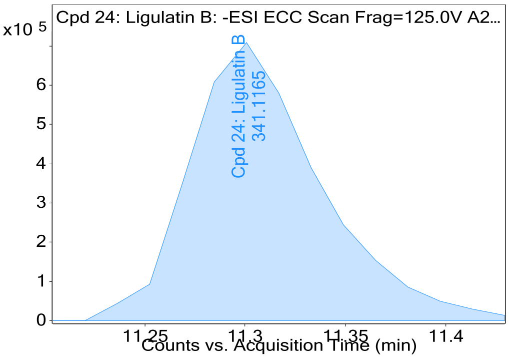

MFE MS Spectrum

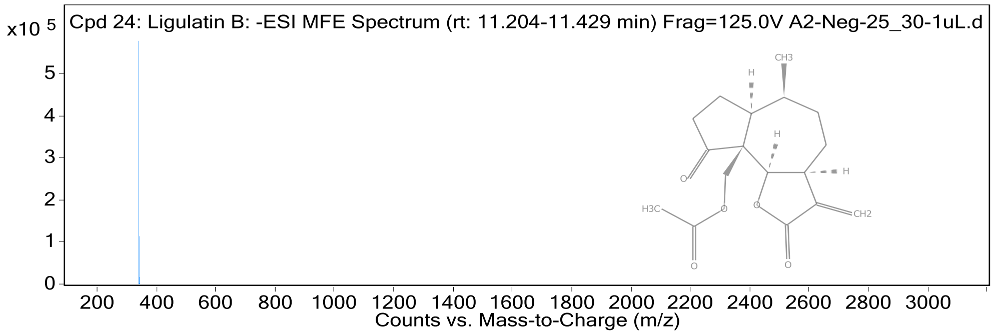

MFE MS Zoomed Spectrum

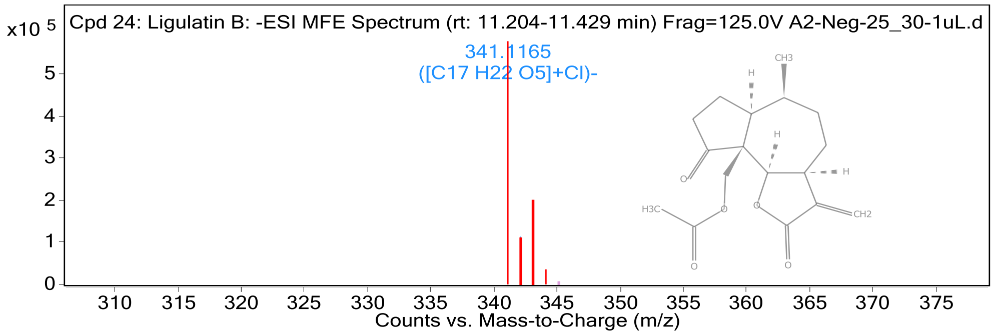

MS Spectrum Peak List

m/z	**z**	**Abund**	**Formula**	**Ion**
341.1165	-1	578040.5	C17 H22 O5	(M+Cl)-
342.1192	-1	113823.44	C17 H22 O5	(M+Cl)-
343.1211	-1	16863.87	C17 H22 O5	(M+Cl)-

344.1224	-1	2024.9	C17 H22 O5	(M+Cl)-

MS Spectrum

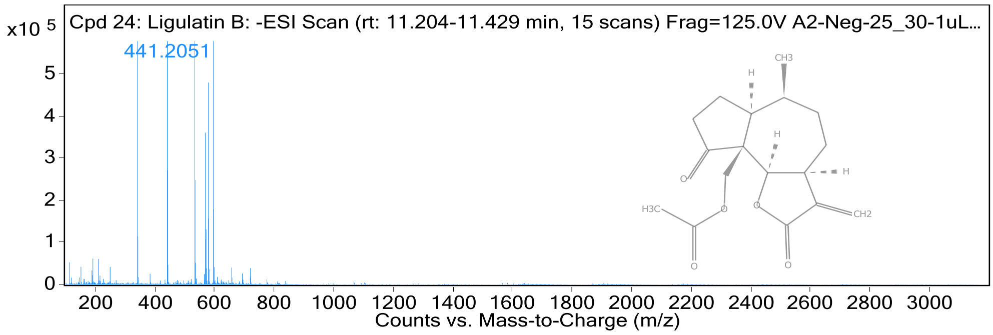

MS Zoomed Spectrum

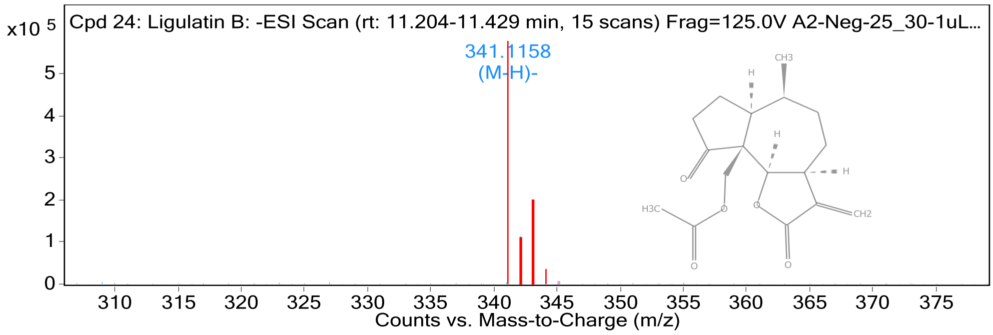

Compound Structure

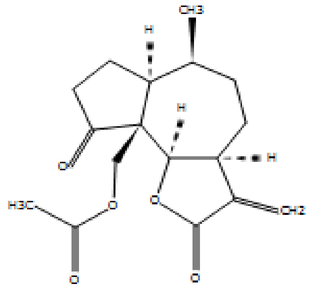

Compound Label	*m/z*	RT	Algorithm	Mass
Cpd 25: 11.304	579.2597	11.304	Find by Molecular Feature	580.267

Compound Chromatograms

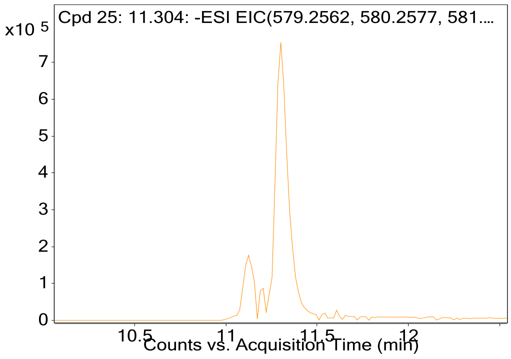

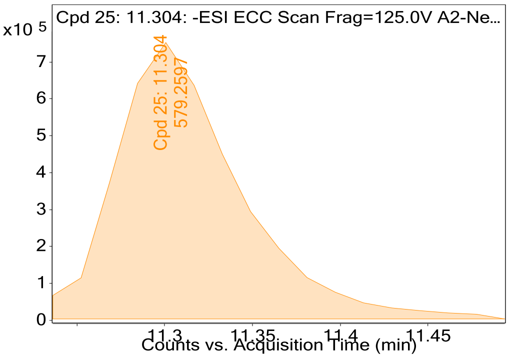

MFE MS Spectrum

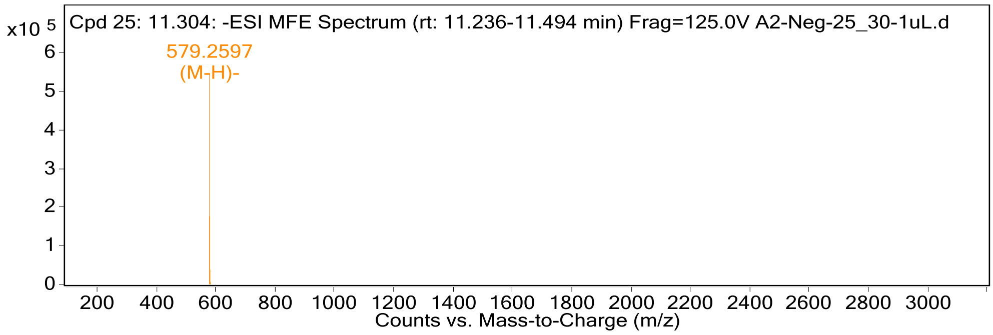

MFE MS Zoomed Spectrum

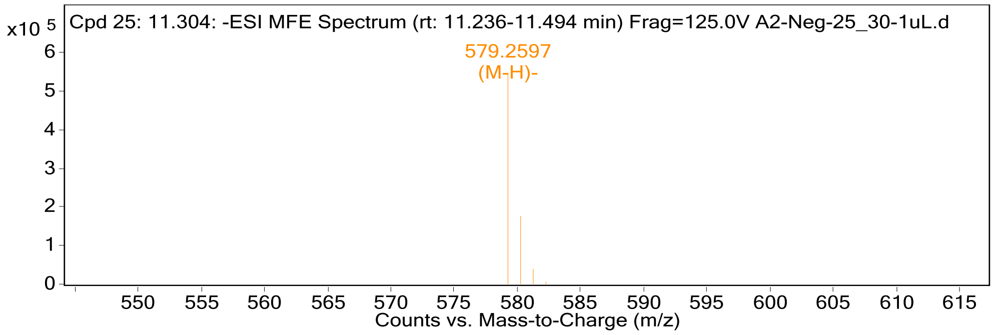

MS Spectrum Peak List

m/z	z	Abund	Ion
579.2597	-1	543521.88	(M-H)-
580.2622	-1	176592.1	(M-H)-
581.2638	-1	38996.37	(M-H)-
582.2649	-1	5822.3	(M-H)-

MS Spectrum

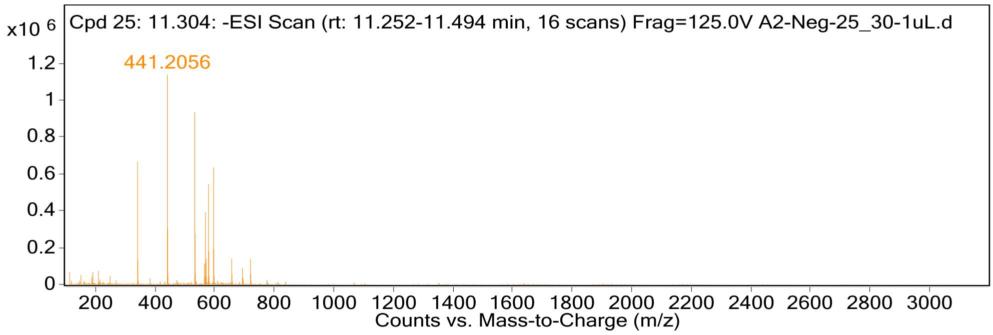

MS Zoomed Spectrum

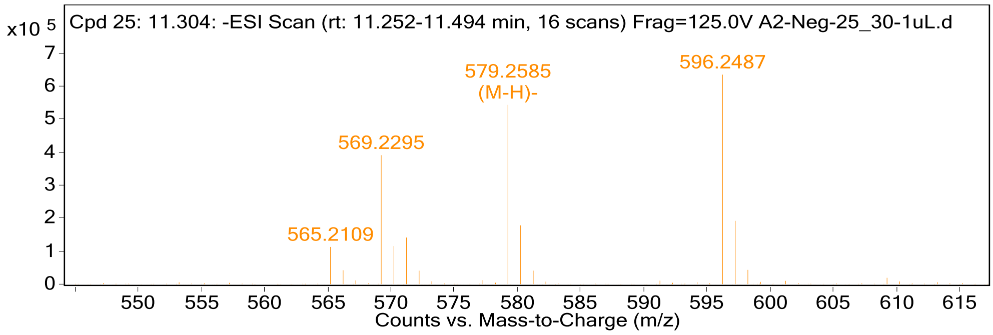

Compound Label	Name	*m/z*	RT	Algorithm	Mass
Cpd 26: bipindogenin	**bipindogenin**	441.2061	11.304	Find by Molecular Feature	406.2369

Compound Chromatograms

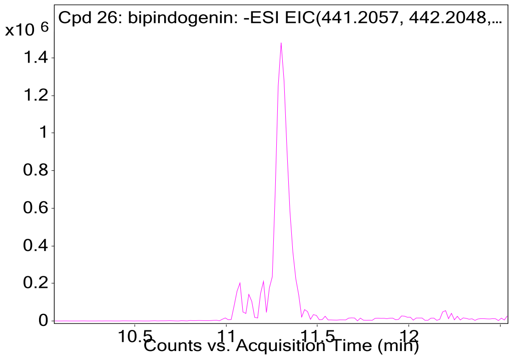

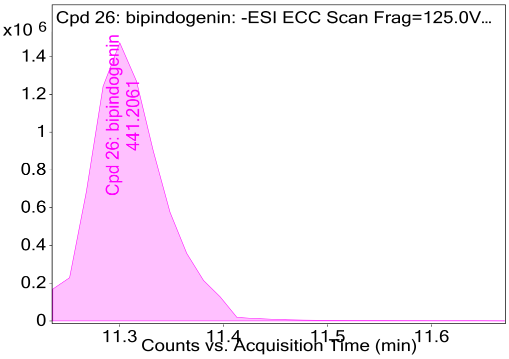

MFE MS Spectrum

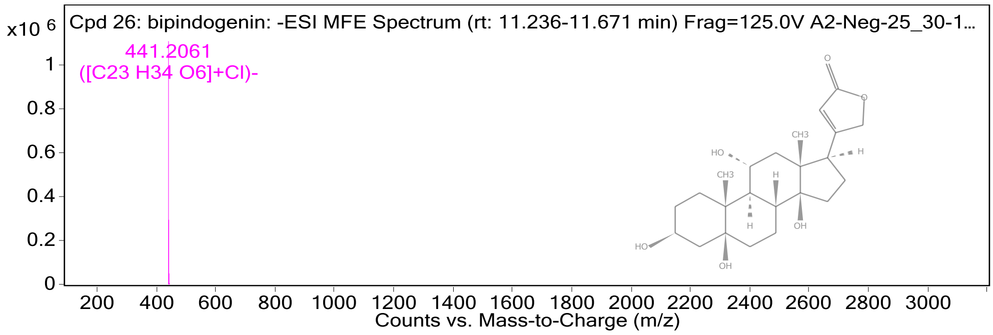

MFE MS Zoomed Spectrum

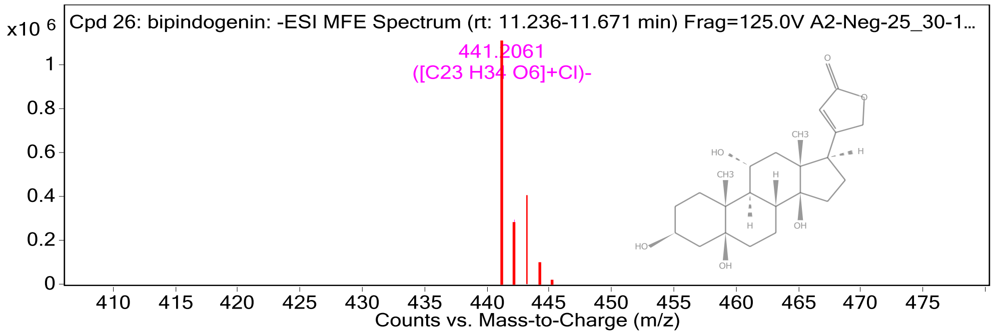

MS Spectrum Peak List

m/z	z	Abund	Formula	Ion
441.2061	-1	1111661.5	C23 H34 O6	(M+Cl)-
442.2089	-1	294983.07	C23 H34 O6	(M+Cl)-
443.2112	-1	49867.67	C23 H34 O6	(M+Cl)-
444.2136	-1	6106.92	C23 H34 O6	(M+Cl)-
445.2103	-1	1125.56	C23 H34 O6	(M+Cl)-

MS Spectrum

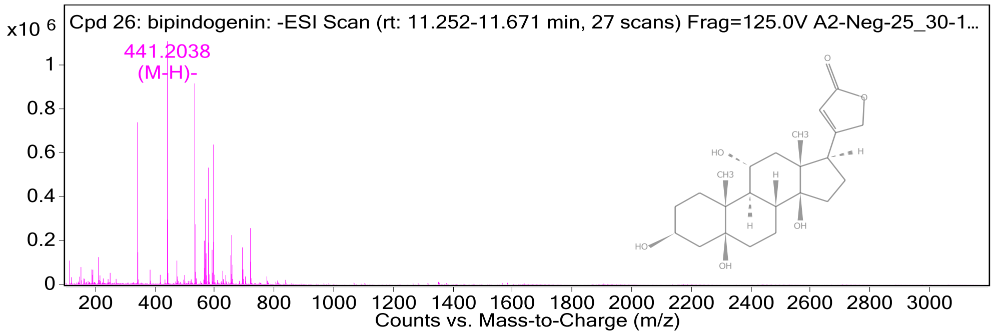

MS Zoomed Spectrum

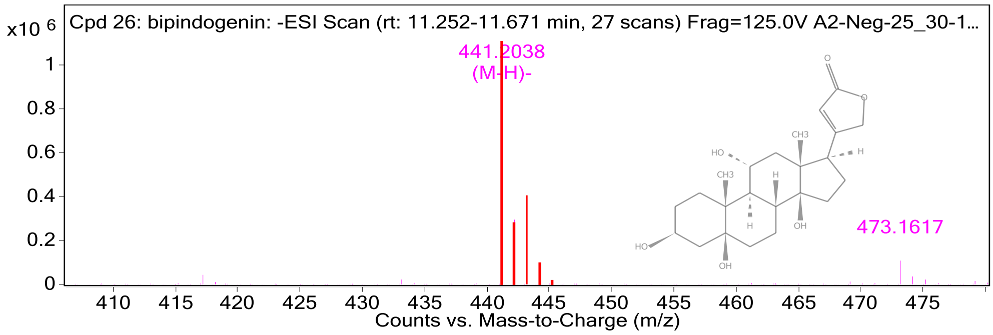

Compound Structure

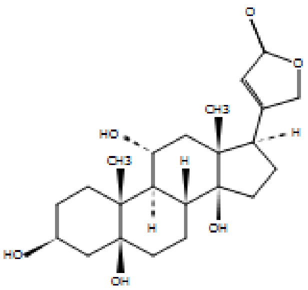

Compound Label	Name	*m/z*	RT	Algorithm	Mass
Cpd 27: Pyropheophorbide a	**Pyropheophorbide a**	533.2543	11.305	Find by Molecular Feature	534.2614

Compound Chromatograms

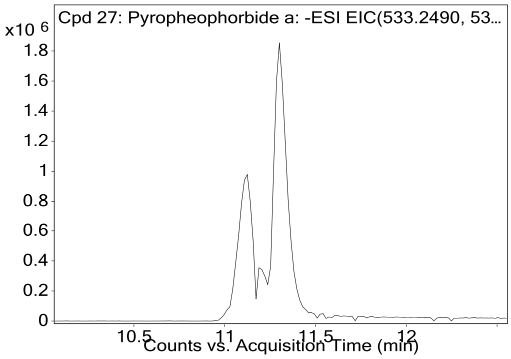

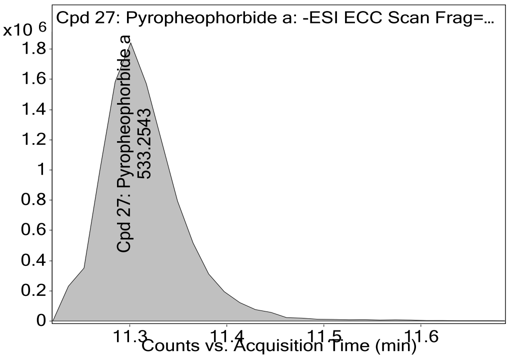

MFE MS Spectrum

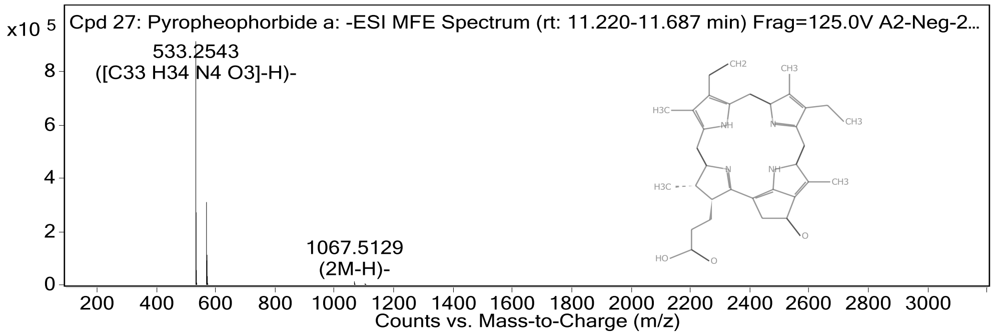

MFE MS Zoomed Spectrum

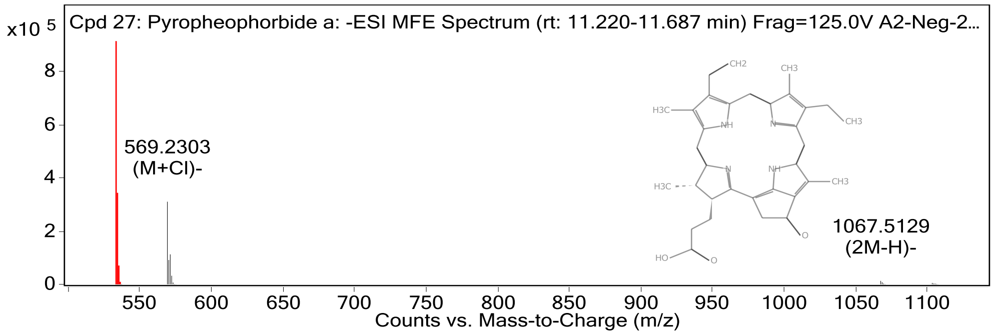

MS Spectrum Peak List

m/z	z	Abund	Formula	Ion
533.2543	-1	914622.44	C33 H34 N4 O3	(M-H)-
534.257	-1	272144.92	C33 H34 N4 O3	(M-H)-
535.2583	-1	55123.65	C33 H34 N4 O3	(M-H)-
536.2614	-1	7800.51	C33 H34 N4 O3	(M-H)-
569.2303	-1	310678.06		(M+Cl)-
570.2329	-1	91532.32		(M+Cl)-
571.2279	-1	112692.01		(M+Cl)-
572.2303	-1	32330.79		(M+Cl)-
1067.5129	-1	12918		(2M-H)-
1068.5163	-1	7808.36		(2M-H)-

MS Spectrum

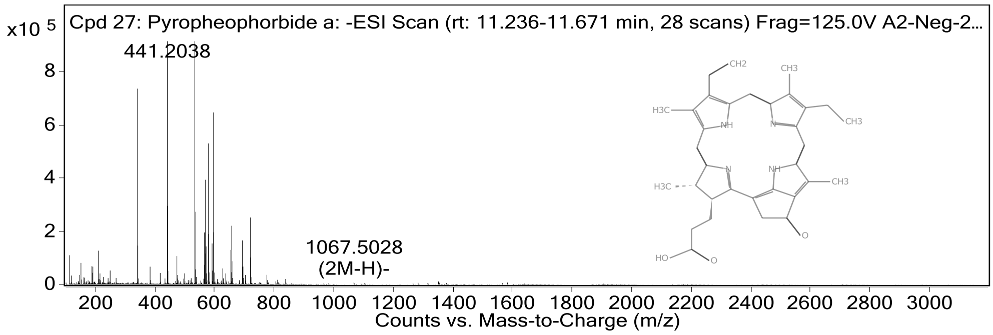

MS Zoomed Spectrum

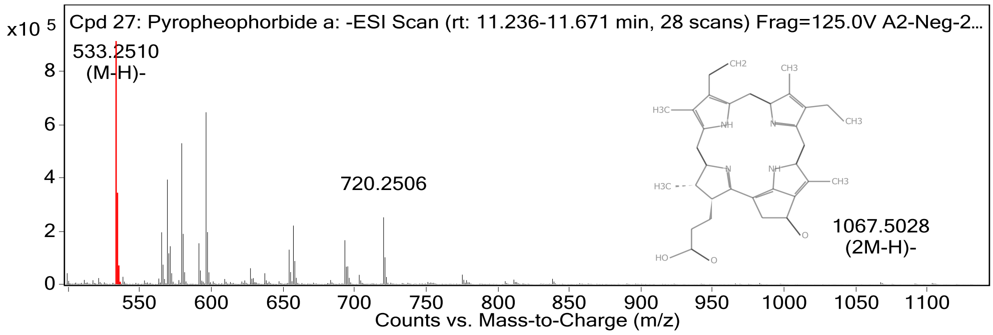

Compound Structure

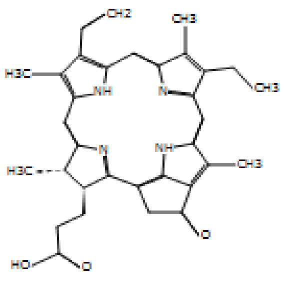

Qualitative Compound Report

Compound Label	*m/z*	RT	Algorithm	Mass
Cpd 28: 11.307	596.2498	11.307	Find by Molecular Feature	597.2571

Compound Chromatograms

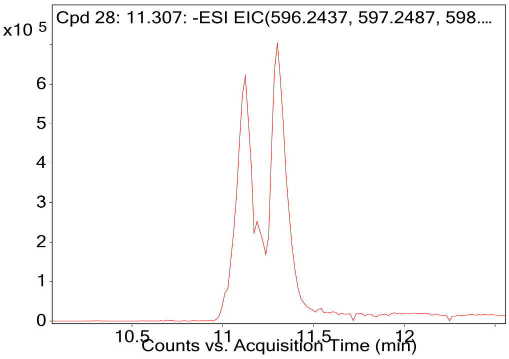

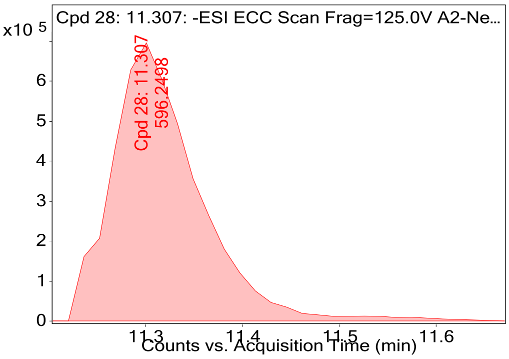

MFE MS Spectrum

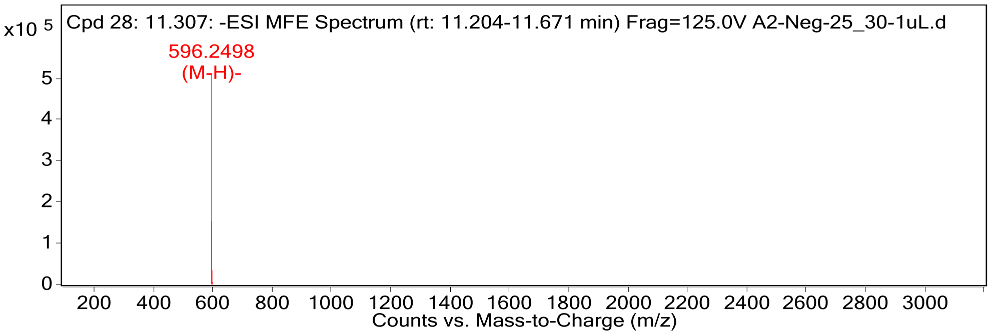

MFE MS Zoomed Spectrum

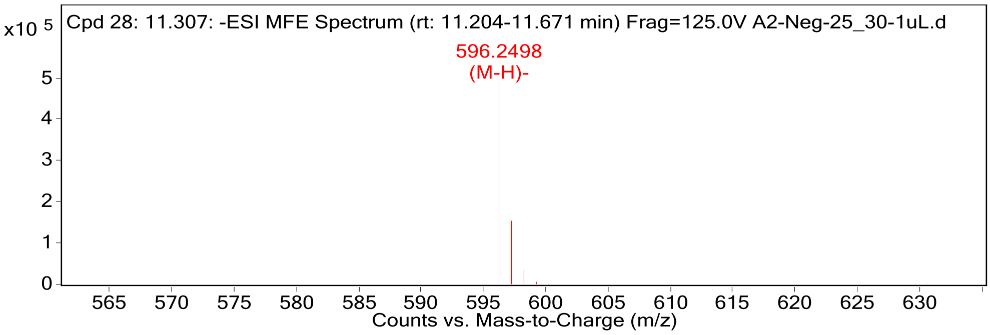

MS Spectrum Peak List

m/z	z	Abund	Ion
596.2498	-1	508292.25	(M-H)-
597.252	-1	153808.61	(M-H)-
598.2538	-1	34481.39	(M-H)-
599.2552	-1	5863.37	(M-H)-
600.2485	-1	773.34	(M-H)-

MS Spectrum

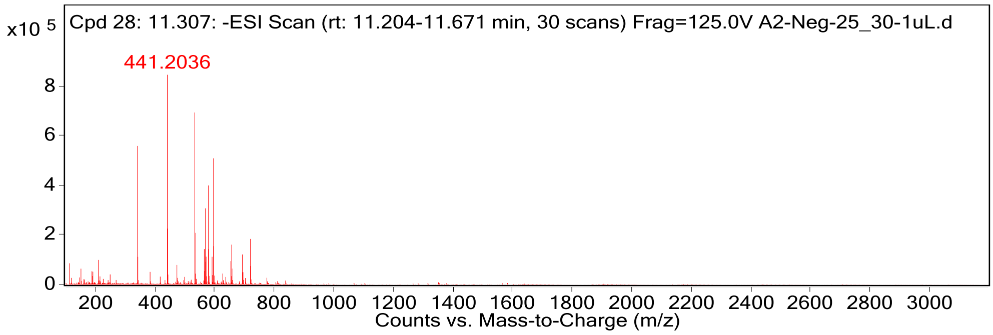

MS Zoomed Spectrum

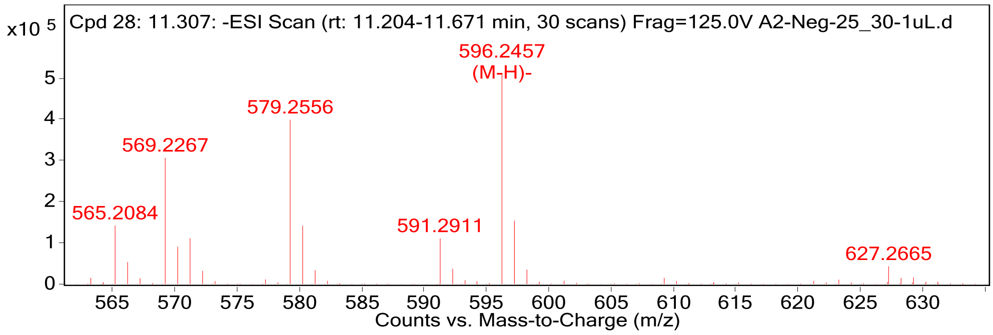

Compound Label	*m/z*	RT	Algorithm	Mass
Cpd 29: 12.265	179.0724	12.265	Find by Molecular Feature	180.0797

Compound Chromatograms

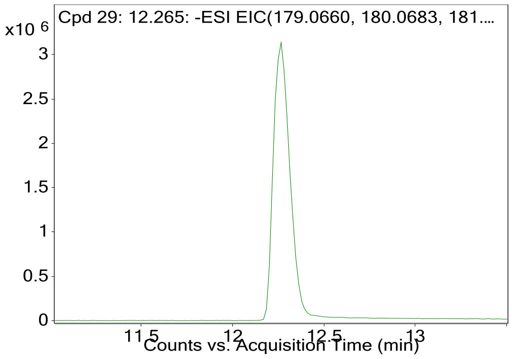

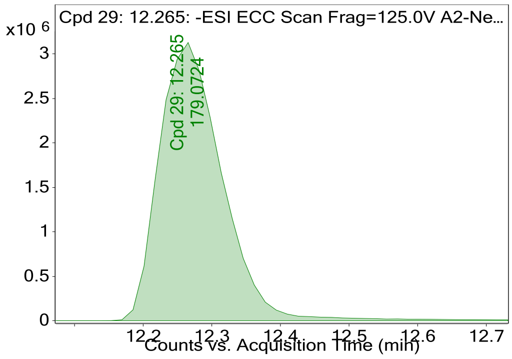

MFE MS Spectrum

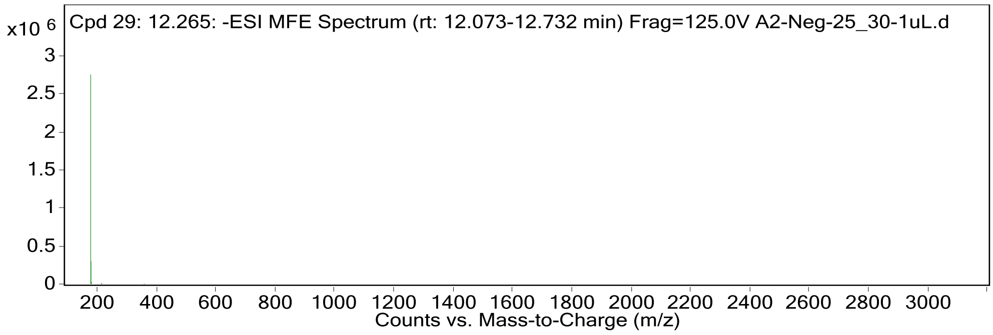

MFE MS Zoomed Spectrum

Qualitative Compound Report

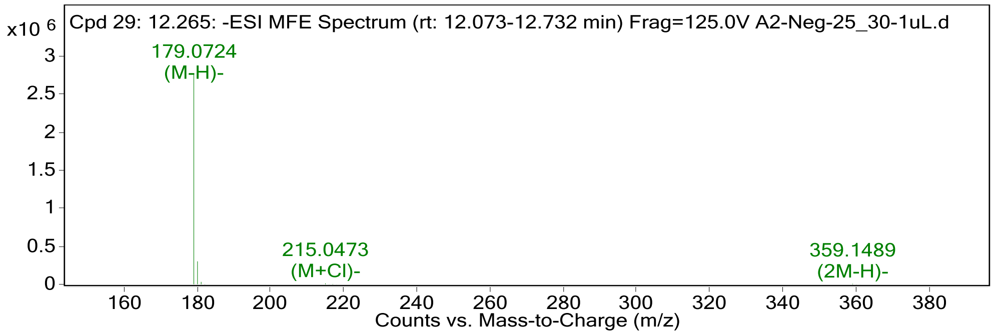

MS Spectrum Peak List

m/z	z	Abund	Ion
179.0724	-1	2756777	(M-H)-
180.0757	-1	302203.45	(M-H)-
181.0772	-1	31954.11	(M-H)-
182.0778	-1	2345.03	(M-H)-
215.0473	-1	12949.31	(M+Cl)-
216.0505	-1	1770.5	(M+Cl)-
217.0452	-1	4697.95	(M+Cl)-
218.0471	-1	702.92	(M+Cl)-
359.1489	-1	7445.11	(2M-H)-
360.1526	-1	1663.78	(2M-H)-

MS Spectrum

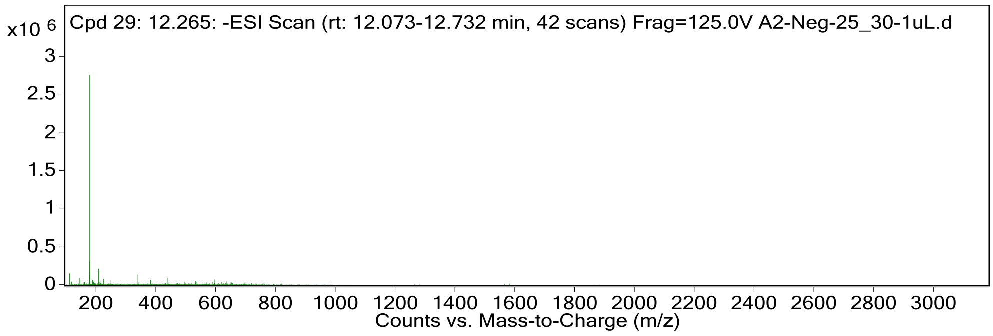

MS Zoomed Spectrum

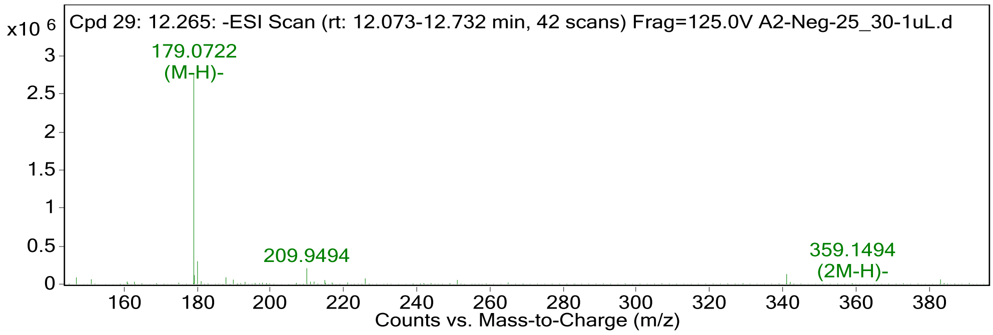

Compound Label	*m/z*	RT	Algorithm	Mass
Cpd 30: 19.171	681.2874	19.171	Find by Molecular Feature	682.2947

Compound Chromatograms

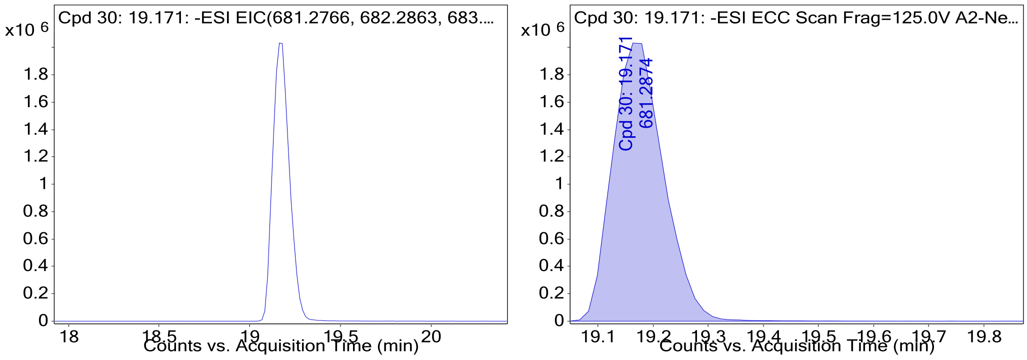

MFE MS Spectrum

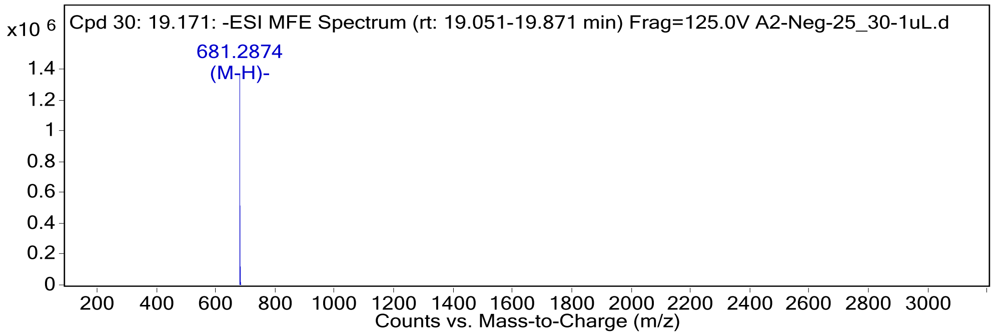

MFE MS Zoomed Spectrum

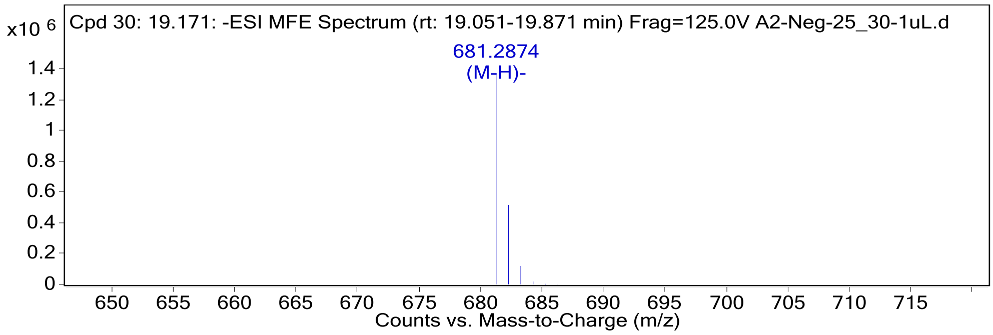

MS Spectrum Peak List

m/z	z	Abund	Ion
681.2874	-1	1362731.38	(M-H)-
682.2907	-1	515550.73	(M-H)-
683.2923	-1	119482.72	(M-H)-
684.2934	-1	18544.23	(M-H)-
685.2956	-1	2730.8	(M-H)-
686.2942	-1	424.63	(M-H)-

MS Spectrum

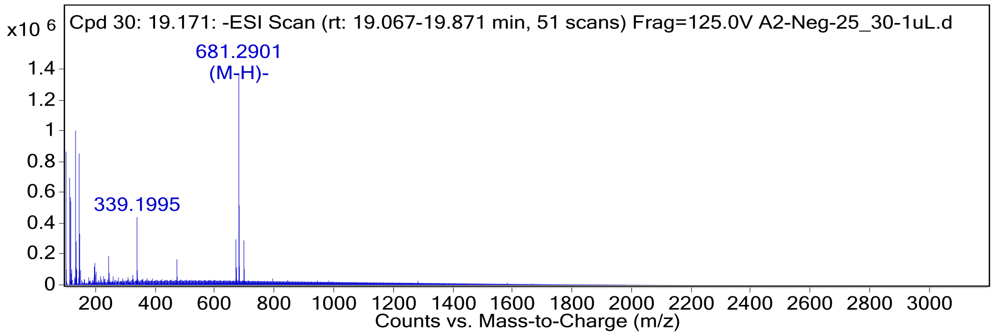

MS Zoomed Spectrum

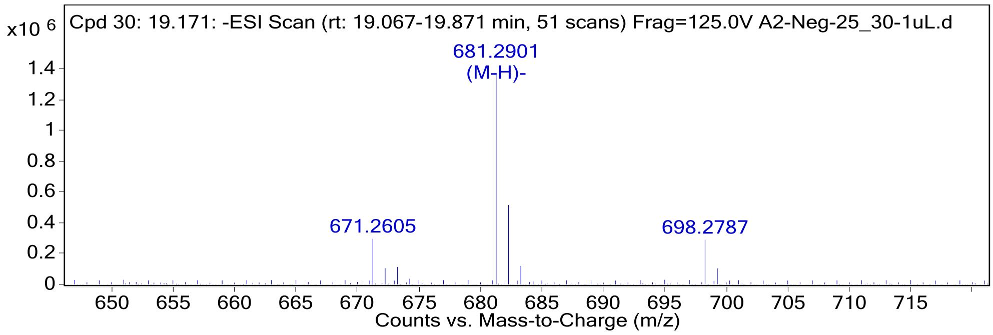

--- End Of Report ---
